# Continuous-time digital twin with analog memristive neural ordinary differential equation solver

**DOI:** 10.1126/sciadv.adr7571

**Published:** 2025-05-28

**Authors:** Hegan Chen, Jichang Yang, Jia Chen, Songqi Wang, Shaocong Wang, Dingchen Wang, Xinyu Tian, Yifei Yu, Xi Chen, Yinan Lin, Qifan Zhu, Yangu He, Xiaoshan Wu, Yi Li, Xinyuan Zhang, Ning Lin, Meng Xu, Yi Li, Xumeng Zhang, Xiaojuan Qi, Zhongrui Wang, Han Wang, Dashan Shang, Qi Liu, Kwang-Ting Cheng, Ming Liu

**Affiliations:** ^1^ Department of Electrical and Electronic Engineering, the University of Hong Kong, Hong Kong, China.; ^2^ School of Microelectronics, Southern University of Science and Technology, Shenzhen 518055, China.; ^3^ Center for Advanced Semiconductors and Integrated Circuits, the University of Hong Kong, Hong Kong, China.; ^4^ Department of Electronic and Computer Engineering, the Hong Kong University of Science and Technology, Hong Kong, China.; ^5^ State Key Lab of Fabrication Technologies for Integrated Circuits and Key Laboratory of Microelectronic Devices & Integrated Technology, Institute of Microelectronics, Chinese Academy of Sciences, Beijing 100029, China.; ^6^ University of Chinese Academy of Sciences, Beijing 100049, China.; ^7^ School of Integrated Circuits, Hubei Key Laboratory for Advanced Memories, Huazhong University of Science and Technology, Wuhan 430074, China.; ^8^ State Key Laboratory of Integrated Chips and Systems, Frontier Institute of Chip and System, Fudan University, Shanghai 200433, China.

## Abstract

Digital twins, which replicate real-world entities through computational models, are transforming manufacturing and automation. While recent advances in machine learning have enabled data-driven digital twin development using discrete-time data and finite-depth models on digital hardware, these approaches face significant limitations. They struggle to capture continuous-time dynamics and model complex systems, and suffer from substantial time and energy overheads due to physically separated storage and processing as well as frequent analog-digital (A/D) conversions. Here, we propose a memristive neural ordinary differential equation (ODE) solver for digital twins. Our approach is intrinsically time-continuous using infinite-depth neural networks to model complex dynamics. Fully analog memristor arrays collocate storage and computation, addressing the von Neumann bottleneck and reducing A/D conversion requirements. We experimentally validate our solver on digital twins of HP variable-resistor model and Lorenz96 dynamics, demonstrating a 166.5-fold/369.3-fold speedup and a 499.0-fold/673.9-fold improvement in energy efficiency, respectively. This work paves the way to future digital twins for Industry 4.0.

## Teaser

Memristive neural ODE solver enables energy-efficient, continuous-time digital twins, accelerating solutions for Industry 4.0.

## INTRODUCTION

Digital twins are computational models that dynamically evolve to deliver precise representations of the structure, behavior, and context of specific physical assets, including components, systems, and processes. As the cyberspace counterpart of physical entities, digital twins are fundamental to Industry 4.0. Recent advances, exemplified by the NVIDIA Omniverse (*1*, *2*), incorporate specialized hardware to streamline operations, reduce operational costs, and boost productivity (*3*, *4*), revolutionizing fields such as manufacturing management and industrial automation (*5*–*12*).

Despite considerable advances, the development and deployment of larger and more sophisticated digital twins encounter significant challenges relating to data, model, and architecture (*13*–*16*). Data-wise, most of the digital twins use discrete-time numerical methods to approximate time-continuous dynamics observed in the real world. While this approach is well suited for digital computers, it inherently introduces truncation errors and information loss due to the sampling of continuous signals (*17*–*19*). Model-wise, the ability of AI-powered digital twins to represent complex dynamics scales with the depth of machine learning models. However, efforts to enhance these capabilities by increasing model depth lead to substantial growth in parameter population and training cost (*20*–*22*). Architecture-wise, traditional digital twins face significant challenges: Frequent analog-digital conversions introduce substantial latency and energy cost (*23*); the von Neumann bottleneck (*24*–*26*) incurs significant time and energy overheads due to the separation of processing unit and memory unit; and the miniaturization of complementary metal-oxide-semiconductor (CMOS) is approaching the limits of Moore’s Law, making further scaling cost-ineffective (*27*, *28*).

To address the aforementioned challenges, we propose an innovative solution: a continuous-time and in-memory neural ordinary differential equation (ODE) solver for infinite-depth digital twins. Our approach offers several advantages over conventional digital twins. Data-wise, our digital twin handles signals from the physical world in a time-continuous manner, effectively eliminating temporal information loss and truncation errors typically associated with discrete time models (*29*–*31*). Model-wise, to address the limitations of finite-depth networks (*32*, *33*), we use neural ODEs to parameterize the derivative of the hidden state in continuous time (*34*). This framework offers an infinite-depth approximation to residual neural networks (ResNets), outperforming recurrent neural networks with the same parameter population and streamlining the training process (*35*–*38*). Architecture-wise, our system leverages emerging memristors (*39*, *40*) as a potential solution to the slowdown of Moore’s Law, enabling in-memory analog vector-matrix multiplications and overcoming the von Neumann bottleneck. In addition, the fully analog system avoids frequent analog-digital conversions, further reducing the extra energy and delay (*41*–*44*).

Here, we validated our approach using 180-nm integrated analog memristor arrays for two different digital twins: the Hewlett-Packard (HP)–coupled variable-resistor model with external inputs and Lorenz96 dynamics as an autonomously evolving system. Compared to conventional digital twins on digital hardware, for HP coupled variable-resistor modeling, our digital twin achieves a remarkable 166.5-fold increase in speed and a substantial 499.0-fold enhancement in energy efficiency while maintaining comparable absolute errors. Moreover, our digital twin demonstrates exceptional scalability in simulated atmospheric pressure extrapolation based on the Lorenz96 dynamics (*19*, *45*), exhibiting a 369.3-fold improvement in speed and a 673.9-fold increase in energy efficiency. Notably, our system also shows superior robustness, demonstrating enhanced resistance to the inherent noise of analog memristor arrays. Our time-continuous, infinite-depth, and in-memory analog system lays the foundation for the next-generation digital twins.

## RESULTS

### Digital twin using memristive neural ODE solver

**Fig. 1.**
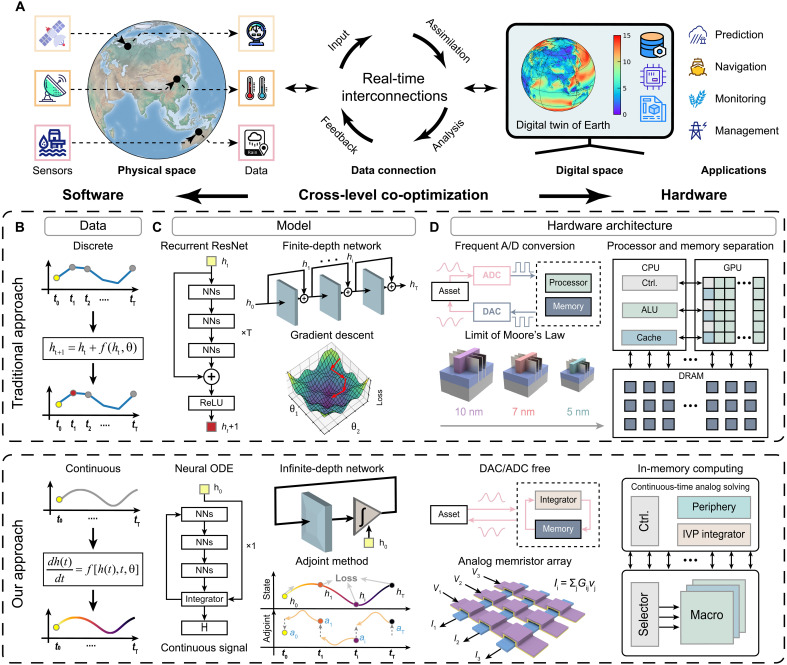
A digital twin using a memristive neural ODE solver compared with a recurrent ResNet-based digital twin on conventional digital hardware in terms of data, model, and architecture. (**A**) Background: The digital twin is a virtual counterpart of a physical asset. For example, environmental sensors of digital twin capture data on atmospheric pressure, temperature, and precipitation. The digital twin models the dynamics of the physical system (e.g., atmospheric physics in this example). (**B**) Data: Traditional approaches discretize continuous data, while our digital twin processes data in its continuous form. (**C**) Model: Recurrent ResNet stacks neural network blocks as a finite-depth network to parameterize a discrete time transition. In contrast, we use a neural ODE, equivalent to an infinite-depth network. NNS, neural networks. (**D**) Architecture: Conventional digital systems frequently perform analog-digital (A/D) conversion, constrained by the von Neumann bottleneck and Moore’s Law slowdown. In contrast, the in-memory neural ODE solver with a memristor array enables fully analog computing without frequent data transfer or ad conversion. CPU, central processing unit; GPU, graphics processing unit; ALU, arithmetic logic unit; DAC, digital-to-analog converter; ADC, analog-to-digital converter; ReLU, rectified linear unit; DRAM, Dynamic Random-Access Memory.

In Fig. 1, we illustrate the implementation of a digital twin using a memristive neural ODE solver and compare it with a recurrent ResNet on digital hardware in terms of data, model, and hardware architecture. Figure 1A illustrates the interconnections between a physical asset and its digital twin, both of which are dynamic systems evolving within their respective state spaces. The digital twin acquires sensory data, such as those from satellites (*8*), and this data stream updates the state of the digital twin, ensuring an accurate representation of the evolving dynamics of the corresponding physical system. Moreover, the digital twin plays a pivotal role in various fields, such as meteorology, where it provides valuable insights for natural disaster prediction, maritime route navigation (*5*), agriculture monitoring (*6*), and city power management (*22*). So far, digital twins have primarily operated on digital computers by repeatedly sampling continuous signals and using finite-depth machine learning models, such as ResNet (*46*), to model a single discrete time transition. However, they face substantial challenges related to data, model, and hardware architecture. These issues can be effectively addressed by our proposed digital twin using a memristive neural ODE solver.

As for data representation, real-world signals are inherently continuous; however, these signals are typically acquired by sensors and subsequently digitized for processing (top data panel of Fig. 1B). Direct processing of the analog waveforms, however, offers the potential to mitigate the energy consumption, latency, and information loss inherent in digitization. In addition, our system, incorporating continuous-time dynamics, faithfully represents the continuous nature of these signals (bottom data panel of Fig. 1B).

As for the model, recurrent ResNets (top model panel of Fig. 1C) are frequently used to parameterize the difference state evolution equation: $h_{t+1}=h_{t}+f\left(h_{t},\theta \right)$, where difference is parameterized by a ResNet *f* with parameters θ, such as a ResNet comprising multiple neural network blocks. While increasing network depth through the addition of neural network blocks enhances the capacity to capture complex features, it also introduces computational burdens, including increased memory requirements and computational cost model.

To augment representation capability and mitigate the training overhead of traditional artificial intelligence (AI)–powered digital twins, we introduce neural ODEs. As shown in the bottom model panel of Fig. 1C, neural ODE integrates multiple neural network blocks mirroring the ResNet, followed by a differential operator. Notably, a recurrent ResNet composed of an infinite number of identical ResNets can be conceptualized as the solution to a neural ODE that characterizes the continuous dynamics of hidden states within the digital twin, as described by (*34*)

$$\frac{dh\left(t\right)}{dt}=f\left[h\left(t\right),t,\theta \right]$$ (1)

The solution for the hidden state *h*(*t*) at any given time can be computed using a black box differential equation solver (see Methods). This approach outperforms finite depth models (e.g., recurrent ResNet) with the same parameter population. It also enables the training of the continuous-time digital twin using the low-cost adjoint method.

As for hardware architecture, traditional digital systems face significant challenges that profoundly impact their overall efficiency (top architecture panel of Fig. 1D). One prominent limitation is the need for frequent analog-to-digital conversions to maintain real-time connectivity with assets. There conversions, performed by power-intensive and sequential ADCs and DACs, increase power consumption and latency, particularly when demanding higher precision and faster sampling rates. Moreover, CMOS technology, currently at 3-nm node, is approaching its physical limits. Quantum tunneling effects result in substantial leakage currents, exacerbating heat dissipation (*47*) and escalating fabrication complexity (*48*). These factors hinder further transistor scaling, posing a significant challenge to Moore’s Law (*49*, *50*). Furthermore, the von Neumann bottleneck, characterized by the separation of processing and memory units, restricts data bandwidth and increases energy consumption. This limitation is particularly acute in data-intensive applications such as AI, where the continuous transfer of massive volume of data between memory and processing units compromises both speed and power efficiency (*51*).

To overcome the aforementioned challenges, our system (bottom architecture panel of Fig. 1D) leverage high-density memristor array, which potentially mitigate the slowdown of Moore’s Law. In addition, memristor arrays perform in-memory vector-matrix multiplications in analog domain, leveraging Ohm’s Law for multiplication and Kirchhoff’s Current Law for summation. This offers a highly parallel and energy-efficient solution to address the von Neumann bottleneck (*52*, *53*). Moreover, this fully analog system support continuous-time computing without digital conversion, enabled by inherent analog computing capabilities of memristors. This approach circumvents the additional energy and delay typically associated with ad conversions.

### Memristive neural ODE solver

**Fig. 2.**
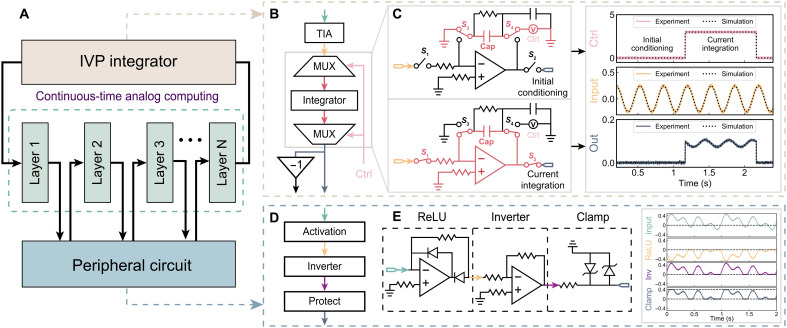
Hardware implementation of memristive neural ODE solver. (**A**) System diagram, consisting of an IVP integrator, peripheral circuit, and analog memristor arrays. (**B**) IVP integrator consists of a transimpedance amplifier (TIA), an integrator with analog multiplexers, and a voltage inverter. It integrates output from the analog memristive neural network and feeds back to the input of the analog memristive neural network, equivalent to the differential operator of a neural ODE. MUX, multiplexer. (**C**) IVP integrator’s dual modes: Initial conditioning and current integration, with oscilloscope-acquired waveforms. (**D**) Peripheral circuit provides analog activation, current-to-voltage conversion, voltage inversion, and protection. (**E**) ReLU and inverter in the periphery circuit convert currents to voltages for the next layer’s inputs, protected by a clamp circuit. The right panel shows oscilloscope-acquired waveform.

**Fig. 3.**
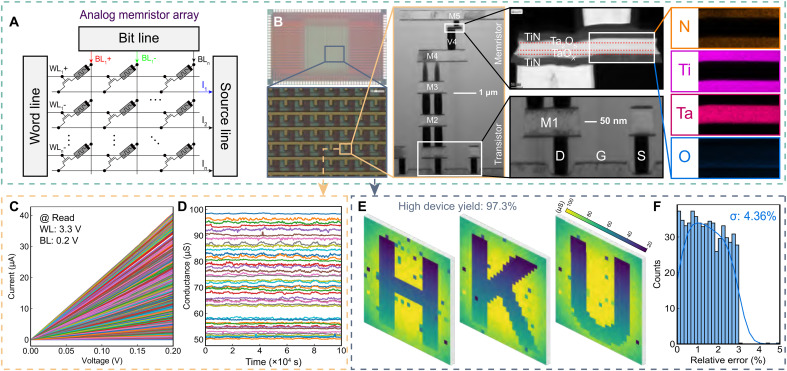
Characteristics of the analog memristor array. (**A**) Analog memristor array uses differential pairs for weight mapping to represent both positive and negative weights. (**B**) Optical photo of the 180-nm 32 by 32 analog memristor array and magnified crossbar, as well as cross-sectional transmission electron microscope (TEM) of an analog memristor, fabricated between the metal 4 and metal 5 layers. (**C**) Analog programming of individual memristors shows more than 64 states. (**D**) Retention exceeding 10^5^ s for different analog conductance. (**E**) Conductance map for the letters H, K, and U with a high device yield of 97.2%. (**F**) Corresponding relative programming error distribution, with a variance of 4.36%.

Figures 2 and 3 illustrate the memristive neural ODE solver and the characteristics of the analog memristor array, respectively.

In Fig. 2A, the memristive neural ODE solver is a time-continuous and analog computing system (see the Materials and Methods for details and fig. S1 for the system photo). This system comprises three building blocks: the initial value problem integrator, the peripheral circuit, and the analog memristor array.

As illustrated in Fig. 2B, the initial value problem (IVP) integrator physically implements the integration operator, which is equivalent to the differential operator of a neural ODE (see the Materials and Methods for the definition of the neural ODE). The IVP integrator operates in two modes: initial conditioning and current integration. The switching between these two modes is facilitated by analog multiplexers. To set the required initial condition for the IVP integrator, the integrating capacitor is precharged, as shown in Fig. 2C. Opening switches *S*_1_ and *S*_2_ isolates the IVP integrator from external inputs, while closing switches *S*_3_ and *S*_4_ connects the capacitor to the power supply, charging it to a preset initial voltage to represent the initial condition of the neural ODE. Transitioning into the current integration mode involves toggling the states of all analog multiplexers, which connects the IVP integrator to the analog memristor arrays to solve the neural ODE as an IVP. Figure 2C also presents an example waveform captured by an oscilloscope, including the control signal (red), input signal (green) through the tia, and integrator output (blue). Experimental integration results closely match those from the circuit simulation, with minimal error (see table S1 for summary of simulation and experimental results).

As shown in Fig. 2D, the peripheral circuit performs analog activation, including ReLU (*54*), current-to-voltage conversions, voltage inversion, and protection. The incorporation of a clamp circuit safeguards the system against overvoltage conditions. The right panel compares the experimental activation and clamped outputs with circuit simulation results.

Figure 3A illustrates the block diagram of the analog memristor array. Each weight of the neural ODE is mapped to the conductance difference between two memristors configured as a differential pair. When the input voltage signals, denoted by the red and green lines, are applied to two adjacent columns with equal amplitude but opposite polarity, this enables the differential pair to encode both positive and negative weights. The neural ODE is physically implemented using 32 × 32 1T1R memristor arrays, as shown in Fig. 3B. The memristors are integrated with CMOS on the 180-nm standard logic platform, positioned between the metal 4 and metal 5 layers. The $\text{TiN}/{\text{TaO}}_{x}/{\text{Ta}}_{2}O_{5}/\text{TiN}$ memristor is fabricated using the back-end-of-line process, as observed by a TEM (see fig. S2 for more information about memristor characteristics). The energy-dispersive X-ray spectroscopy (EDS) mapping results provide a detailed illustration of the elements distribution (Ti, N, Ta, O) across various layers. The unique dual-layer structure of ${\text{Ta}}_{2}O_{5}/{\text{TaO}}_{x}$ is also revealed by the distribution of O (see Methods for further details on fabrication).

Figure 3C showcases the multilevel conductance of a single memristor, achieving high-precision 6-bit resolution, corresponding to more than 64 distinct conductance states. Figure 3D demonstrates stable analog conductance by repeatedly applying 0.2 V to selected memristors in the array for more than 10,000 s. Figure 3E shows three programmed letters on analog memristor arrays, demonstrating a high yield of 97.3% and reasonable programming accuracy at the array level (see figs. S3 and S4 for the programming scheme and numerical conductance of the letters). Figure 3F presents the corresponding histogram depicting the distribution of programming errors, defined as the relative error between the target conductance and the post-programming conductance for responsive memristors (see fig. S5 for the analysis of patterns), showing a variance of 4.36%.

### Digital twin for the HP coupled variable-resistor model

**Fig. 4.**
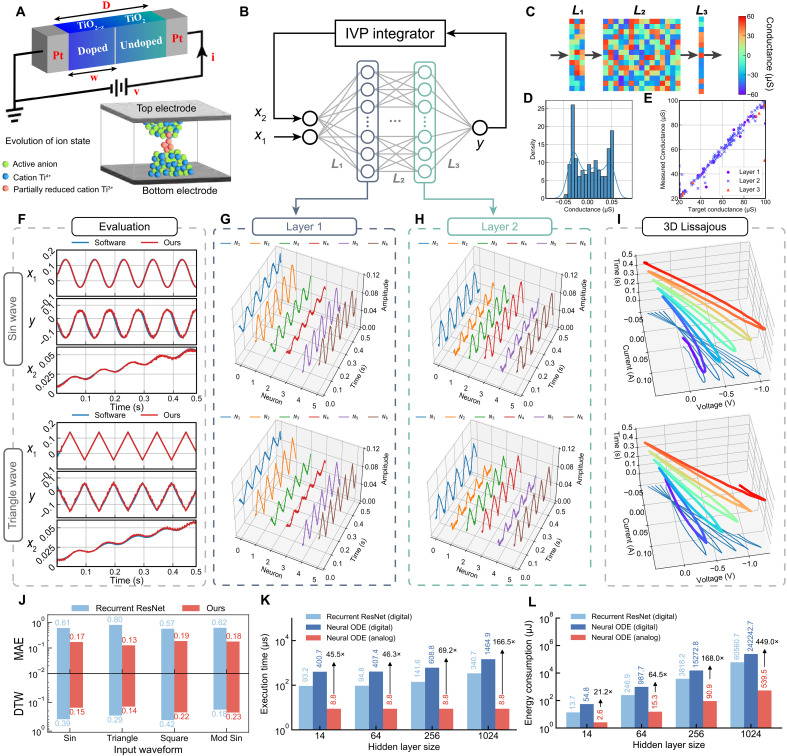
Experimental digital twin of an HP-coupled variable-resistor model. (**A**) Schematic of the HP-coupled variable-resistor model showing how the boundary between doped and undoped regions evolves under applied voltage. (**B**) Framework of our system, with the neural ODE implemented on a three-layer analog memristive neural network. (**C**) Experimentally programmed differential conductance in the three analog memristor arrays. (**D**) Histogram illustrating the distribution of programmed conductance. (**E**) Statistical analysis showing less than 2.2% error within the conductance range of 20 to 100uS. (**F**) Comparison of experimental voltage waveforms from our system and software ground truth upon sinusoidal and triangular input stimulation. (**G** and **H**) Experimental voltage waveforms of selected *L*_1_ and *L*_2_ hidden neurons during inference. (**I**) Three-dimensional, time-dependent Lissajous plot depicting the nonlinear current-voltage (*I*-*V*) relationship, induced by sinusoidal and triangular input simulation and their corresponding states. (**J**) Comparison of modeling errors with recurrent ResNet-based digital twin across four stimulation waveforms. (**K** and **L**) Comparison of speed and energy consumption between recurrent ResNet on digital hardware, neural ODE on digital hardware, and our system across different hidden layer sizes.

To validate our approach, we first construct a digital twin of a coupled variable-resistor model proposed by the HP laboratories (*55*), which is characterized by a chaotic manifold governed by an ODE. Figure 4A illustrates the dynamics of the HP-coupled variable-resistor model. Here, an external bias, *v*(*t*), drives oxygen anions toward the electrode, thereby modifying the electrical conductivity through changes in the valence of metal cations. Assuming ohmic electrical conduction, the resistance of the HP-coupled variable-resistor model follows (*56*)

$$\frac{v}{i}=R_{\text{ON}}\frac{w}{D}+R_{\text{OFF}}\left(1-\frac{w}{D}\right)$$ (2)

where $R_{\text{ON}}$ and $R_{\text{OFF}}$ are resistivities of the doped and undoped region, respectively. *D* is the dimension between two metal terminals. The state variable of a HP coupled variable-resistor model follows

$$\frac{\mathrm{dw}}{\mathrm{dt}}=\mu_{v}\frac{R_{\text{ON}}}{D}i=f\left(w,v,t,\theta \right)$$ (3)

where w is the state variable representing the boundary location between doped and undoped region with average ion mobility $\mu_{v}$. To model the HP-coupled variable-resistoras a black box, we parameterize the right-hand side of the equation using a neural network f, forming a neural ODE.

Figure 4B shows the schematic diagram of our system. The analog input signals, *x*_1_ and *x*_2_, pass through the three-layer neural network physically implemented on three analog memristor arrays (2 × 14, 14 × 14, and 14 × 1) and peripheral circuits. The output voltage *y* from the last layer is integrated by the IVP integrator before serving as the input *x*_2_ to the neural network. This closed-loop circuit represents the integral form of the neural ODE. The weights of the neural networks are optimized offline before deployment on analog memristor arrays (see the Materials and Methods). Figure 4C and D present the experimental conductance map of the differential pairs in three analog memristor arrays, along with the distribution of conductance. Figure 4E illustrates the low statistical programming errors of analog memristor arrays, with an average relative error of 2.2%.

To demonstrate that our digital twin behaves as a HP-coupled variable-resistor model described by Eq. 3, we tested it with four types of input stimulation (sine, triangular, rectangular, and modulated sine waveforms) and simulated the HP-coupled variable-resistor model’s evolution. Figure 4F compares results from our digital twin with software ground truth (see note S1 and table S1 for a detailed summary of the simulation and experimental results). Here, *x*_1_, *y*, and *x*_2_ are monitored using an oscilloscope. The experimental waveforms closely match the software-based ground truth, demonstrating the memristive digital twin’s capacity to model, interpolate, and extrapolate complex HP-coupled variable-resistor model dynamics. Figure 4 (G and H) shows the experimental voltage outputs of six selected neurons in middle layers *L*_1_ and *L*_2_, respectively. Figure 4I presents a three-dimensional time-dependent Lissajous plot of the current-voltage relation (*I*-*V*) characteristic, depicting the nonlinear relationship between the sinusoidal/trigonometric input *x*_1_ and corresponding states *x*_2_ over a continuous time span from 0 to 0.5 s.

Figure 4J compares the modeling errors under different stimulation conditions between our proposed system and a conventional digital twin using a recurrent ResNet on digital hardware. Our approach achieves lower errors, with an mean absolute value (MAE) of 0.17 and a dynamical time wrapping (DTW) score of 0.15 (see Methods for the definition of the DTW loss function). In contrast, recurrent ResNet on digital hardware exhibits higher errors, with a MAE of 0.61 and a dtw score of 0.39 (see fig. S6 for details on the training process of the recurrent ResNet and neural ODE). Next, we evaluate the speed and energy efficiency of our system against advanced graphic processing units (GPUs). As shown in Fig. 4K, a memristive neural ODE digital twin achieves 166.5-fold faster speeds than GPU-based neural ODE at a hidden layer size of 1024. As the network scales up, the benefits of our system’s time-continuous and analog in-memory computing become more pronounced (see note S2 for an execution time and energy consumption comparison with advanced GPUs). Figure 4l presents the energy consumption comparison among three systems at varying hidden layer sizes. The light-blue and dark-blue bars indicate the estimated energy consumption of recurrent ResNet and neural ODE on a state-of-the-art GPU, respectively. Notably, our system (red bars) exhibits significantly lower energy consumption, ~539.5 μJ per forward pass. In comparison, at a hidden layer size of 1024, recurrent ResNet and neural ODE on a GPU consume around 60.6 and 242.2 mJ, respectively. These results highlight a remarkable 112.2-fold and 449.0-fold improvement in energy efficiency compared to recurrent ResNet and neural ODE, respectively, both on digital hardware.

### Digital twin for multivariate time-series extrapolation using Lorenz96 dynamics

**Fig. 5.**
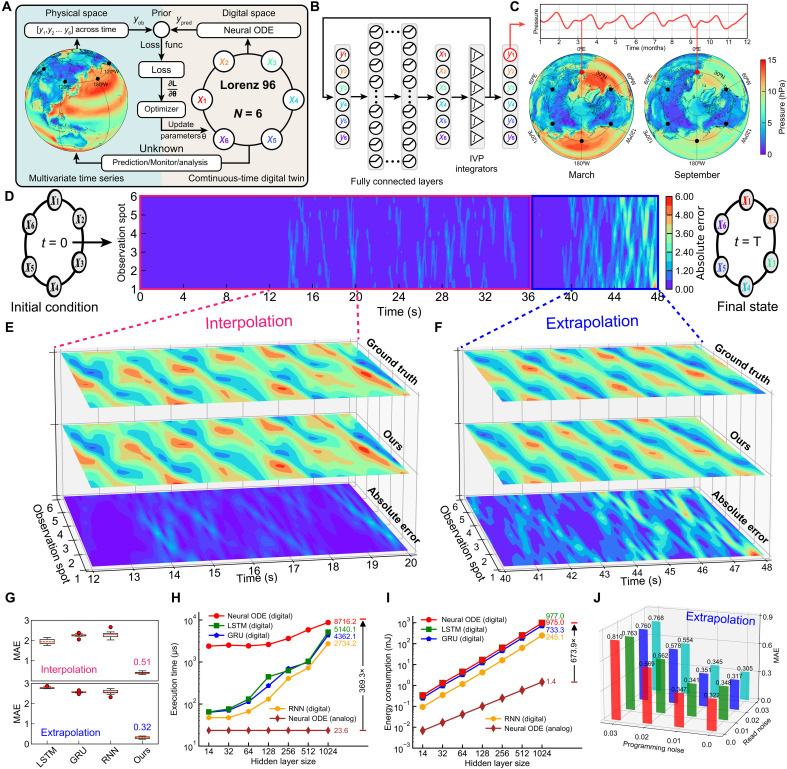
Multivariate time series extrapolation of Lorenz96 dynamics with our digital twin. (**A**) Illustration of training framework for our digital twin. (**B**) Architecture of the digital twin, featuring fully connected layers and a group of six IVP integrators, facilitates the dynamic self-evolution of six-dimensional states ($y_{1}$ to $y_{6}$) without external stimulation. (**C**) Temporal evolution of atmospheric pressure $y_{1}$ at 30°N, 0°E, modeled using Lorenz96 dynamics, depicting annual fluctuations in Earth’s atmospheric pressure with detailed snapshots for March and September. (**D**) Mean absolute error (MAE) between the output of our system and the ground truth, with the interpolation stage (0 to 36 s) highlighted in red and the extrapolation stage (36 to 48 s) marked in blue. (**E** and **F**) Contour plots showcasing the selected interpolation (0 to 8 s) and extrapolation (36 to 44 s) phases, including the ground truth, output results from our system, and the MAE. (**G**) Comparison of interpolation (red) and extrapolation (blue) error for four different models: LSTM, GRU, and RNN on digital hardware and our system. (**H** and **I**) Comparison of execution time and energy consumption for four systems (neural ODE, LSTM, GRU, and RNN on digital hardware and our analog system) executing an interpolation task from 0 s to 36 s, across increasing hidden layer sizes. (**J**) Noise analysis of our system, evaluating the impact of various combinations of read and programming noise during the inference. Our analog system is robust to read noise.

In addition to the HP coupled variable-resistor model, we assess the performance of our digital twin in modeling the Lorenz96 dynamics, a simplified mathematical representation of atmospheric physics, which is widely used in atmospheric variability and climate prediction (*19*, *45*). In Fig. 5A, we show the training process for the digital twin using physical space observations $y_{\text{ob}}$. The loss function quantifies the disparity between observed and predicted outcomes $y_{\text{pred}}$, enabling the digital twin to accurately mirror the state of the physical asset (*57*–*59*).

The Lorenz96 dynamics is described by a series of ODEs

$$\frac{{\mathrm{dx}}_{i}}{\mathrm{dt}}=\left(x_{i+1}-x_{i-2}\right)x_{i-1}-x_{i}+F,\;i=1,\ldots ,n,\;n>3$$ (4)

with a periodic boundary condition denoted as $x_{i+n}=x_{i}$, where n∈ℕ and F∈ℝ are the parameters. It represents atmospheric waves circulating along a latitude circle, with each segment $x_{i}$ representing a distinct meteorological variable such as pressure or precipitation, where each index *i* indexes latitude segments.

Figure 5B illustrates our system, which consists of a four-layer fully connected neural network and six IVP integrators. Unlike the HP-coupled variable-resistor modeling, the evolution of Lorenz96 dynamics is autonomous, without external stimulation. The training and testing processes are similar to those used for the previous digital twin of the HP-coupled variable-resistor model. Despite its autonomous evolution without external input, this digital twin incorporates random noise as a regularizer during training to enhance its stability and robustness. Figure 5D, along with the expanded views in Fig. 5 (E and F), displays the post-training error of our digital twin operating in free running mode (see note S3 for details on the two modes employed in time-series modeling). The time axis is segmented into two phases: the interpolation phase (0 to 36 s) is depicted by the red box, and the extrapolation phase (36 to 48 s) is depicted by the blue box, across six variables (see note S4 and table S2 for further details on scalability in complex dynamic datasets). The color indicates the magnitude of deviation between the digital twin’s outcomes and the ground truth of the Lorenz96 dynamics, showing minimal discrepancy (see fig. S24 for additional error analysis). Furthermore, the digital twin accurately predicts the nonlinear dynamic behavior over a period of 2.17 Lyapunov times (see note S5 and table S3 for an evaluation of Lyapunov time in relation to model performance), while the influence of various hyperparameters is investigated (see note S6 and figs. S28 and S29 for details on the training performance of neural ODE).

To benchmark the effectiveness of our digital twin, we conduct a series of performance evaluations against other multivariate time series (MTS) models, including long short-term memory (LSTM), gated recurrent unit (GRU), and recurrent neural network (RNN), on the state-of-art GPUs. These models are compared on the basis of error, computational speed, and energy efficiency while maintaining consistent parameter settings and training conditions (see note S2 and table S4 for a detailed comparison of speed and energy consumption).

In Fig. 5G, the errors over ten trials are shown, highlighting the mean performance and variation between different models on the interpolation (red) and extrapolation (blue) tasks (see note S7 and table S5 for a detailed accuracy comparison between the standard digital neural ODE and our approach). The average error of our neural ODE digital twin for the interpolation task is ~0.51, while for the extrapolation task, it is ~0.32 (see note S8 for the limitations of analog ODE solvers). In contrast, models such as LSTM, GRU, and RNN exhibit significantly larger errors (see fig. S30 for an analysis of the impact of hidden layer sizes).

Figure 5H presents the analysis of execution time for a single inference sample across five models, each with an increasing hidden layer sizes. The estimated execution time for the neural ODE, LSTM, GRU, and RNN models on state-of-the-art GPU architecture is 8.72, 5.14, 4.36, and 2.73 ms, respectively, with 1024 hidden neurons. In comparison, the execution time of a neural ODE on analog memristors is 23.6, which represents enhancements of approximately 369.3-fold, 218.0-fold, 184.8-fold, and 115.9-fold over the neural ODE, LSTM, GRU, and RNN models on digital hardware, respectively. Figure 5I presents the energy consumption of each model across seven different hidden layer sizes, with 14, 32, 64, 128, 256, 512, and 1024 hidden neurons. The memristive Neural ODE demonstrates projected energy efficiency improvements, surpassing the neural ODE, LSTM, GRU, and RNN models on digital hardware (1024 hidden neurons) by factors of 189.7, 147.2, 100.6, and 37.1, respectively.

Analog circuits often encounter noise issues, which can lead to reduced accuracy and unexpected signal fluctuations. Figure 5J examines the effect of read and programming noise on the performance of our digital twin in extrapolation tasks. Our findings, each averaged more than 10 repetitions, indicate that read noise can even reduce extrapolation errors compared to a noise-free environment. For instance, with read noise at 2% and no programming noise, the model achieves a lower MAE of 0.317, in contrast to 0.322 without read noise. This highlights the resilience of our digital twin to read noise (see note S9 for details on reading and programming noise).

## DISCUSSION

Here, we present a continuous-time and in-memory neural ODE solver for infinite-depth AI-powered digital twins. This approach effectively addresses the data, model and architecture limitations of conventional digital twins. Through validation across two tasks-modeling HP coupled variable-resistor and extrapolating Lorenz96 dynamics, our methodology shows significant improvements. The experimental digital twin for HP-coupled variable-resistor model demonstrates a substantial enhancement over the conventional digital twins, with our system achieving a 166.5-fold increase in speed and a 499.0-fold reduction in energy consumption. Moreover, in extrapolating Lorenz96 dynamics, our system achieves speed improvements of 369.3-fold, accompanied by energy savings of 673.9-fold when benchmarked against neural ODEs on digital hardware. Our system holds great promise for advancing efficient and accelerated digital twin technology, benefiting Industry 4.0 initiatives.

## MATERIALS AND METHODS

### Fabrication of analog memristor arrays

Here, a 1-kb analog memristor array, comprising a 32 × 32 1T1R crossbar, is fabricated using the 180-nm technology node. Following the deposition of the Via4 layer during the backend-of-line process, memristors are stacked on the drain side of the transistors. Initially, a 40-nm TiN layer, serving as the bottom electrode, is formed through physical vapor deposition (PVD). Subsequently, a dual TaO-based dielectric layer with different oxygen compositions is designed and deposited by atomic layer deposition to enhance the analog properties of memristors. The dielectric layer included a 50-nm lower O concentration ${\text{Ta}}_{2}O_{5}$ layer and a 10-nm higher-O-concentration ${\text{TaO}}_{x}$ layer. Then, a 40-nm TiN layer, fabricated by PVD, is used as the top metal. Last, the top metal 5 process is completed to finish the fabrication. The 1T1R crossbar structure is implemented by connecting the gate and source terminals of the transistors as the column-wise word lines (WLs) and source lines (SLs), respectively, and the top electrode (TE) terminals of the individual memristor as the row-wise bit lines (BLs). The device underwent a post-annealing treatment under vacuum conditions at a temperature of 400 for a duration of 30 min. This treatment significantly improved the performance of the array, resulting in devices that exhibit high endurance and reliability.

### The fully analog computing system

The fully analog computing system integrates three 180-nm analog memristor arrays, each equipped with selection transistors, along with switch matrices and peripheral circuits to enable system integration. System control is provided by a personal computer (PC) and an Advanced RISC Machine Microcontroller Unit (ARM MCU) (STM32F407ZGT6) (see fig. S1 for detailed hardware specifications). The analog memristor arrays function in two modes: programming mode for weight mapping and multiplication mode for vector-matrix operations.

#### Programming mode

In programming mode, the target conductance within the analog memristor array is precisely tuned to match the software-defined weight values. The specific memristor selected for programming is controlled by a switch matrix, which activates analog multiplexers [Transmission Multiplexer (TMUX)] for the Word Lines (WLs), Bit Lines (BLs), and Source Lines (SLs). This configuration ensures accurate routing by switching the targeted memristor’s selection transistors to a low-resistance state while maintaining all other memristors in a high-resistance state to prevent interference. Isolating the selected memristor enables direct interfacing with the B1500A Semiconductor Device Analyzer (B1500A), allowing for accurate and efficient programming. The tuning process is executed via a Python script running on a PC, which communicates with the B1500A to precisely adjust memristor parameters. This process ensures that the memristor conductance closely aligns with the neural network weights, achieving the desired analog representation (see fig. S3 for details of the programming scheme).

#### Multiplication mode

In multiplication mode, the switch matrix boards, controlled by MCUs, are directed by a PC to connect with the peripheral circuitry. A waveform generator applies voltages to the system, while an oscilloscope records the outputs. For vector-matrix multiplication, analog voltages from the waveform generator are routed to the BLs of the analog memristor array via a dual-channel analog multiplexer (TMUX1134, Texas Instruments). The resulting currents on the SLs are converted into voltages by trans-impedance amplifiers (TIAs) (OPA4990, Texas Instruments, TI). Voltage activation is achieved by a ReLU module with a dual-diode configuration (1N4148 diodes) within the TIA. These voltages are then inverted and integrated by an inverting amplifier and integrator (OPA4990, Texas Instruments). Last, the output voltages are fed back into the analog neural network block, forming a closed-loop system that emulates the neural ODE.

### Dynamical time wrapping

DTW is a versatile algorithm widely employed to measure the similarity between two temporal sequences that may differ in speed or length (*60*). Let us consider two time series, $X=\left\{x_{1},x_{2},\ldots ,x_{n}\right\}$ and $Y=\left\{y_{1},y_{2},\ldots ,y_{m}\right\}$.To apply DTW, we construct an *n* × *m* matrix where each element $d_{i,j}$ represents the distance between the ith element of *X* and the jth element of *Y*, typically using the Euclidean distance

$$d_{i,j}=\;|x_{i}-y_{j}|$$ (5)

Then, we compute the cumulative distance $D_{i,j}$ for each element of the matrix using dynamic programming, following the recursive relation

$$D_{i,j}=d_{i,j}+\text{min}\left(D_{i-1,j},D_{i,j-1},D_{i-1,j-1}\right)$$ (6)

The objective of DTW is to find a path from (1,1) to (*n*,*m*) that minimizes $D_{i,j}$, representing the total match cost between the two time series. To achieve this, we initialize $D_{0,0}$ to 0 and set $D_{0,j}$ and $D_{i,0}$ to infinity (or a sufficiently large number) to ensure that the matching path always starts at (1,1). The recursive relation allows us to calculate $D_{i,j}$ based on the minimum of the three neighboring elements: $D_{i-1,j-1}$, $D_{i-1,j}$, and $D_{i,j-1}$. This approach enables the elastic transformation of the time axes, allowing for optimal matching between the sequences, even when they vary in speed or length.

### Comparison of ResNet and neural ODE architectures

The ResNet, composed of an infinite number of identical neural network blocks, is defined as follows (*46*)

$$h_{t+1}=h_{t}+f\left(h_{t},\theta \right)$$ (7)

where the gradient *f* is parameterized by a neural network with parameters θ. Skip connections enhance the ResNet’s ability to learn residual features, thereby improving training and performance. These iterative updates can be interpreted as an Euler discretization of a continuous transformation.

On the contrary, neural ODEs describe continuous evolution using an ODE specified by a neural network *f*, formulated as follows (*34*)

$$h_{t_{1}},h_{t_{2}}\ldots ,h_{t_{N}}=\text{ODESolve}\left(h_{t_{0}},f,\theta ,t_{0},\ldots ,t_{N}\right)$$ (8)

Given observation times $t_{0},\ldots ,t_{N}$ and an initial state $h_{t_{0}}$, an ODE solver computes $h_{t_{1}},h_{t_{2}},\ldots ,h_{t_{N}}$, representing the latent state at each observation.

### Training method of continuous-time digital twin

To train the neural ODE model *f*, we use the adjoint state method (*34*). This method computes the gradient of the loss function with respect to the hidden state at each time stamp, known as the adjoint $a\left(t\right)=\frac{\partial L}{\partial h_{t}}$. By defining the state vector carefully, we can compute the necessary integrals to solve for *a*(*t*) and $\frac{\partial L}{\partial \theta }$ in a single call to the ODE solver.

#### HP-coupled variable-resistor model

We create a training set called $y_{\text{true}}$, comprising 500 data points sampled from Eq. 2 with a time interval of $\Delta t=1\times 10^{-3}s$. Our objective is to minimize the MAE between these points and the corresponding trajectories predicted by our digital twin, denoted as $y_{\text{pred}}$, throughout all time steps. Regarding the neural network architecture, we use the ReLU activation function for all layers except the output layer.

#### Multivariate time-series extrapolation

To train our digital twin for extrapolating the MTS data, we generate Lorenz96 dynamics with a sequence length of 2400 using the first 1800 points for training (interpolation) and the remaining 600 for testing (extrapolation). The digital twin, with a dimension of *d* = 6, is implemented as a three-layer neural network with 64 neurons per hidden layer. The initial conditions are set as $\begin{array}{c} \left[y_{1},y_{2},\ldots ,y_{6}\right]=\left[-1.2061,0.0617,1.1632,-1.5008,-1.5944,-0.0187\right] \end{array}$, and training is performed on 0.02-ms Lorenz96 trajectories. DTW is used as the loss function to measure the dissimilarity between predicted and true trajectories ($y_{\text{true}}$), with gradients calculated via the adjoint method. Model parameters θ are optimized using the adaptive moment estimation (Adam) algorithms. A fourth-order Runge-Kutta (RK4) method serves as the ODESolve.

## Supplementary Materials

### This PDF file includes:

Figs. S1 to S34 Tables S1 to S6 Supplementary Notes S1 to S9 References
