# Supplementary material for: Continuous-time digital twin with analog memristive neural ordinary differential equation solver
Source: Sci Adv. 2025 May 28;11(22):eadr7571. doi: 10.1126/sciadv.adr7571 (PMC13109951; doi:10.1126/sciadv.adr7571)
Supplement: Supplementary file 1 — Figs. S1 to S34 Tables S1 to S6 Supplementary Notes S1 to S9 References [file sciadv.adr7571_sm.pdf]

Science Advances
AAAS

Supplementary Materials for

Continuous-time digital twin with analog memristive neural ordinary differential equation solver

Hegan Chen *et al.*

Corresponding author: Zhongrui Wang, zrwang@eee.hku.hk; Jia Chen, jjiachen@ust.hk; Han Wang, hanwang6@hku.hk; Dashan Shang, shangdashan@ime.ac.cn

Sci. Adv. **11**, eadr7571 (2025)
DOI: 10.1126/sciadv.adr7571

This PDF file includes:

Figs. S1 to S34
Tables S1 to S6
Supplementary Notes S1 to S9
References

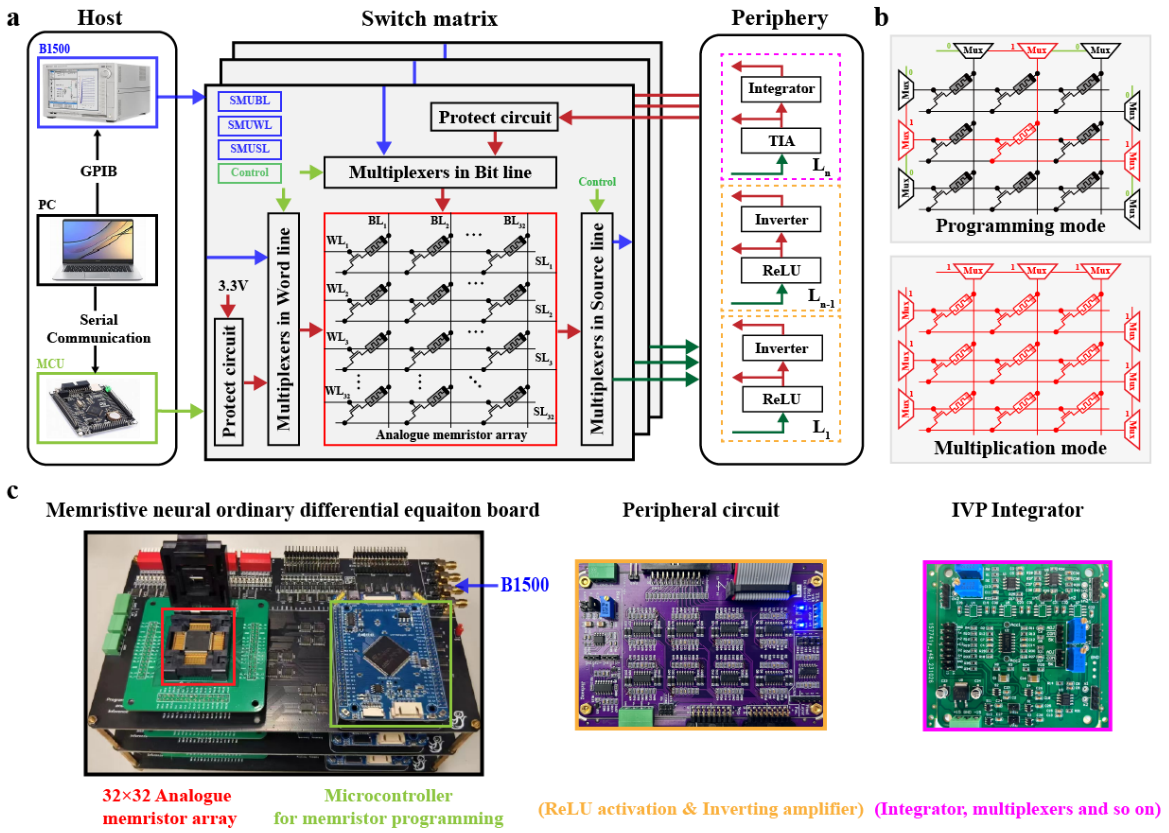

Fig. S1: Experimental setup and printed board details. a, The fully analog computing system comprises three main components: the host, the switch matrix, and the peripheral circuit. The host components consist of a microcontroller (MCU), a personal computer (PC), and a Semiconductor Device Paramester Analyzer (B1500A). The switch matrix, which hosts the analog memristor array, supports various operations such as programming and multiplication via a series of analog multiplexers and incorporates protection circuitry. The peripheral circuit encompasses the analog Rectified Linear Unit (ReLU), Transimpedance Amplifier (TIA), an inverting amplifier, and an integrator. Configuration of the MCU is managed through serial communication from the host, while the PC uses GPIB to communicate with the B1500A for programming the analog memristor arrays, as indicated by the blue line. These arrays store weight matrices and, once programmed, connect to the peripheral circuit within the switch matrix to perform vector-matrix multiplication, as depicted by the red and green lines. **b,** The diagram of the analog memristor arrays illustrates the integration of analog multiplexers for Word Lines (WLs), Bit Lines (BLs), and Source Lines (SLs). These multiplexers are crucial for enabling smooth transitions between programming and multiplication modes, thereby enhancing the flexibility and efficiency of the analog memristor arrays and facilitating their adaptation to diverse computational demands. **c,** Photograph of the fully analog computing system.

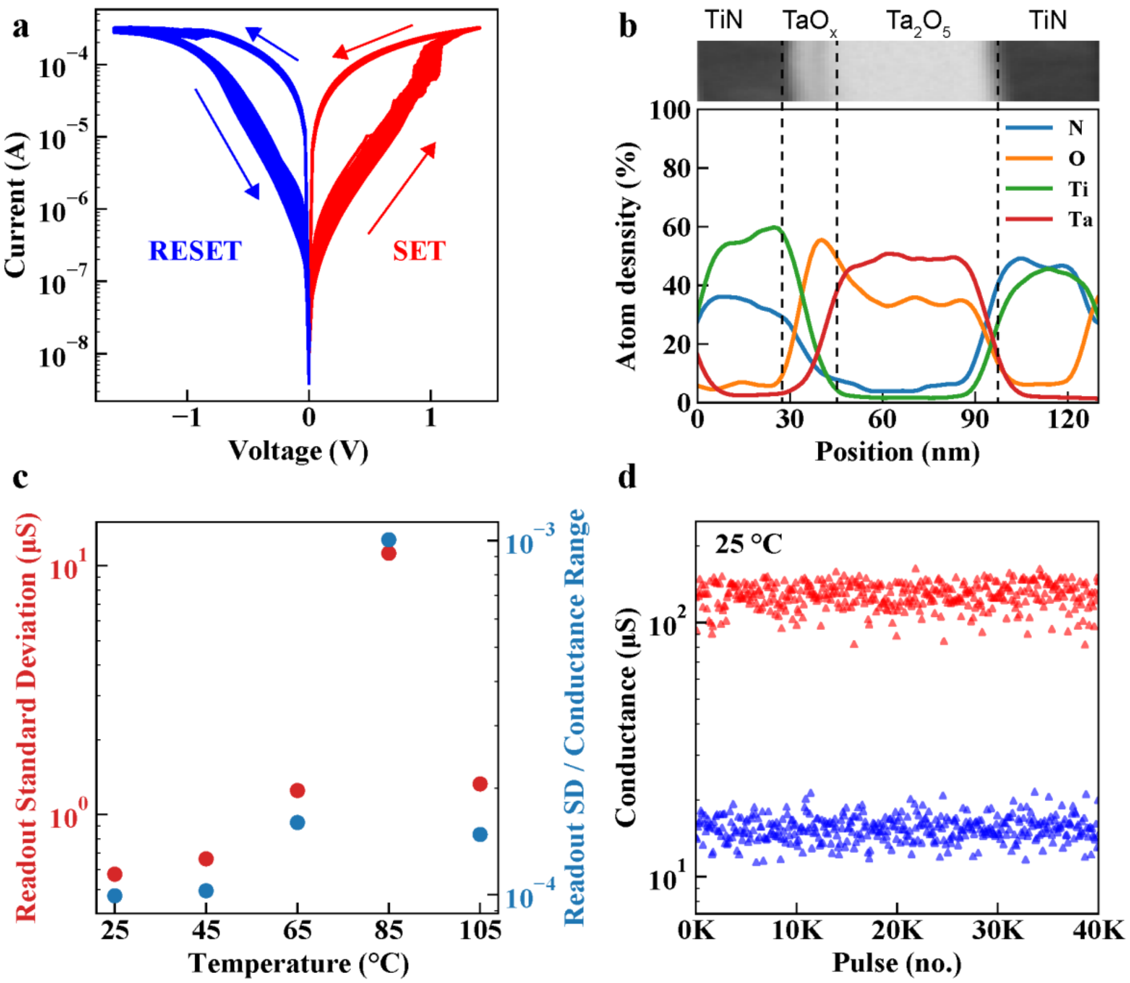

Fig. S2: The electrical characteristics of a memristor in the 180 nm 32×32 analog memristor array. a, The typical current-voltage (IV) curves for a $TiN/TaO_x/Ta_2O_5/TiN$ memristor. The Set/Reset cycle is performed 400 times, with arrows indicating the direction of the voltage scan. **b,** Energy-dispersive X-ray Spectroscopy (EDS) line profile of a memristor. **c,** The conductance stability of the device is evaluated under various temperature conditions, affirming its operational reliability in diverse environmental settings. **d,** The memristor exhibits exceptional endurance, maintaining flawless functionality even after 40,000 Set/Reset cycles.

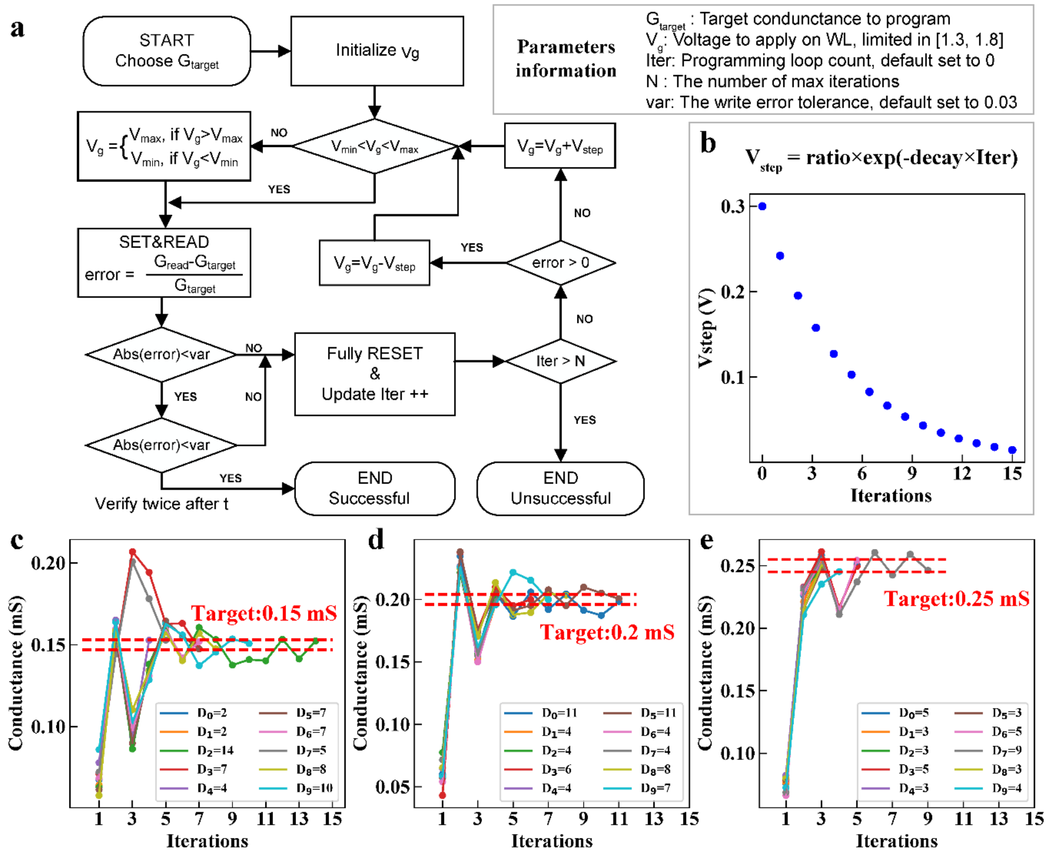

Fig. S3: Programming scheme of the analog memristor array. a, The flowchart illustrates the memristor programming process. To program a specific memristor to achieve a target conductance, denoted as G_{target}, the first step is to initialize the appropriate WLs voltage (V_g) required for programming the memristor. It is important to note that V_g is constrained within the safe operating range defined by V_{min} and V_{max}. Once the appropriate V_g is determined, a Set operation is performed on the memristor using this voltage. The current conductance is then read to evaluate the error. Based on the relative error compared to a defined threshold value (*var*), the system determines the subsequent action. If the relative error exceeds the threshold, a full RESET operation is executed on the memristor. The iteration count and V_g are updated for the next Set operation, as long as the iteration count does not surpass the maximum iteration limit (N) set. If the maximum iteration count is exceeded, the programming process is considered unsuccessful. In contrast, if the relative error falls below the threshold var, the system waits for a specified time period (t_{verify}) before reading the conductance again. If the relative error remains below the threshold during the verification phase, the programming is deemed successful. **b,** The WLs voltage update function determines the appropriate voltage level to be applied to the WLs during the Set operation. **c-e,** Conductance variation during the memristor programming process to achieve target values of 0.15 mS, 0.2 mS, and 0.25 mS.

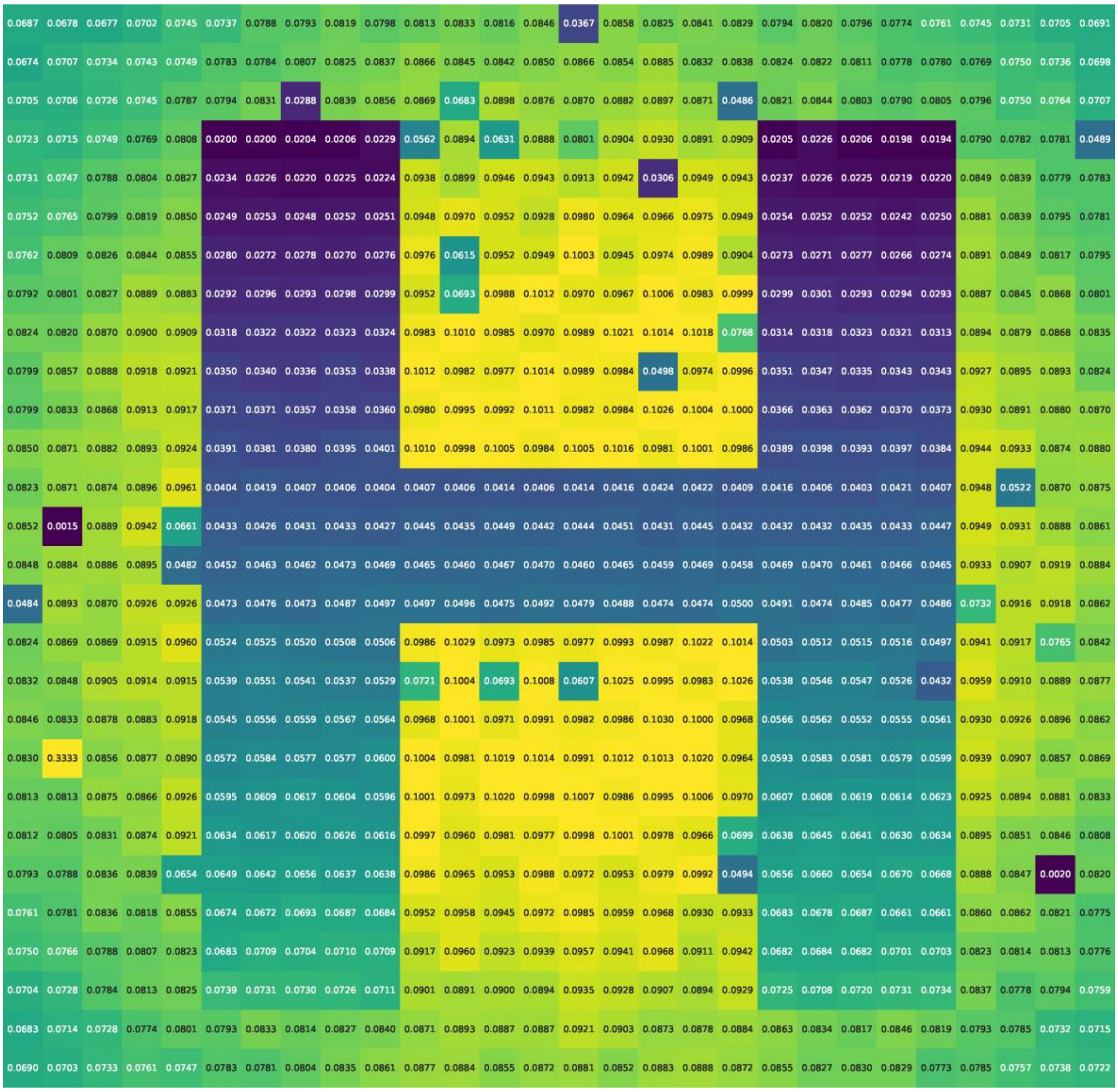

Fig. S4: Numerical results for the conductance of the 'H' tuning in an analog memristor array.

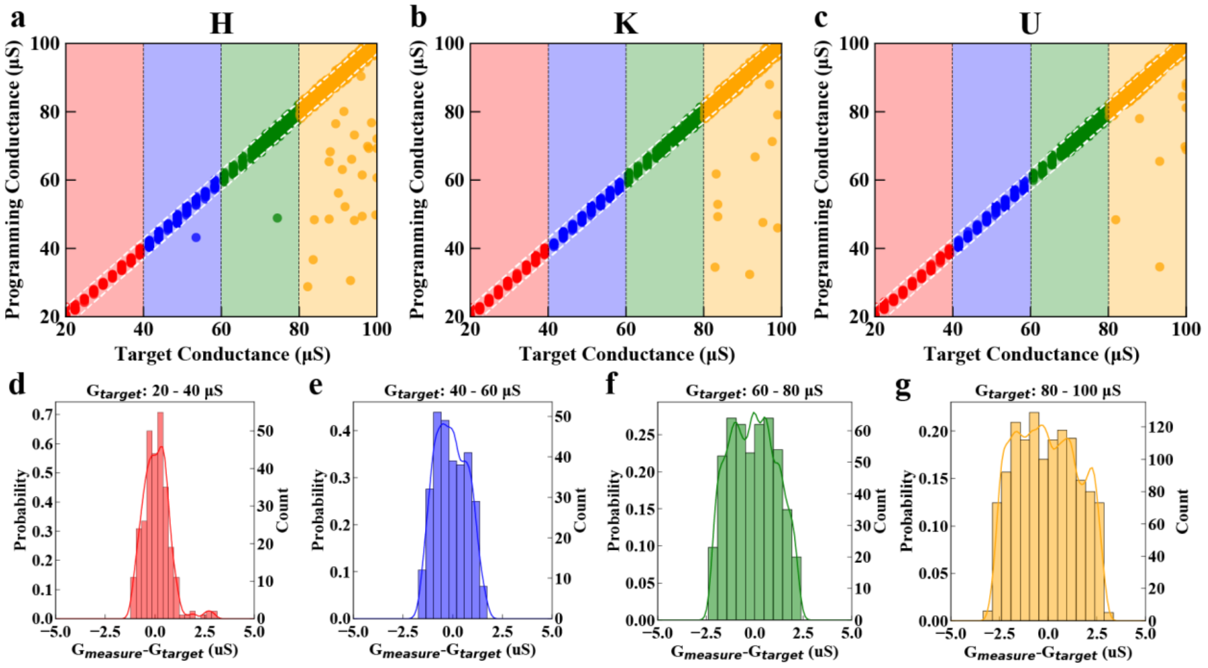

Fig. S5: The comparison between target conductance values and the experimentally programmed values for the a, 'H', **b,** 'K', and **c,** 'U' in the three analog memristor arrays, where the white dashed lines show the tolerance value (3%) during the conductance tuning process. The red, yellow, green, and blue areas represent the various conductance range including **d,** 20 µs - 40 µs, **e,** 40 µs - 60 µs, **f,** 60 µs - 80 µs and **g,** 80 µs - 100 µs, which illustrates the impact of conductance range on the programming error distribution.

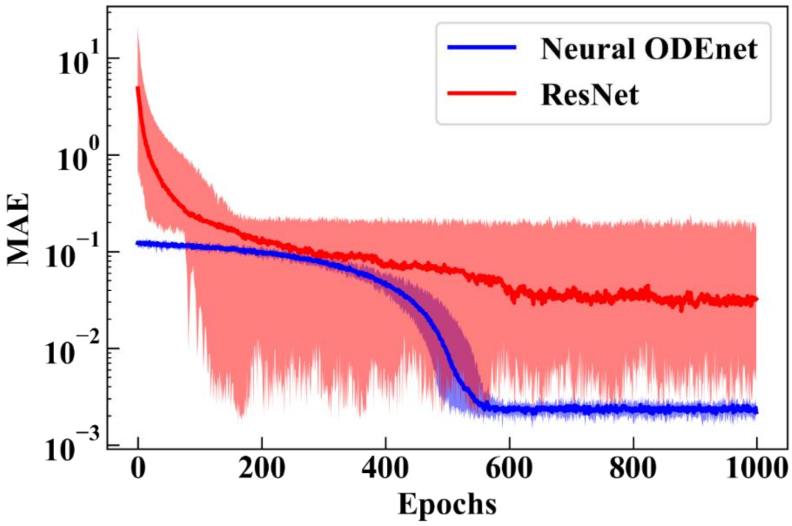

Fig. S6: Comparison between neural ODE and recurrent ResNet models in terms of training performance. The solid line in the plot represents the trajectory, while the surrounding shaded areas depict the error intervals for recurrent ResNet (shown in red) and neural ODE (shown in blue).

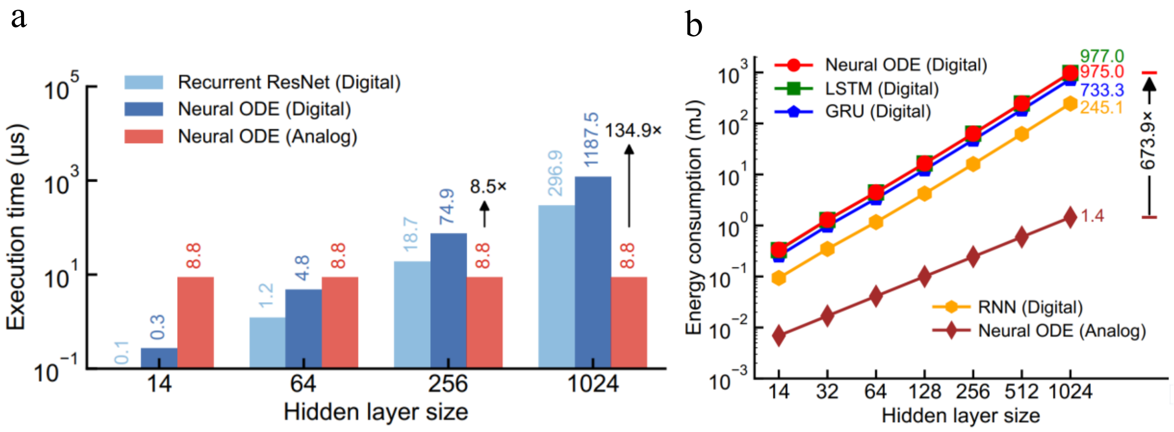

Fig. S7: Comparison of execution time benchmarking against GPU peak throughput for our analog Neural ODE system and other digital systems for two tasks: a, digital twin of the HP coupled variable-resistor model and **b,** the digital twin of Lorenz96 model.

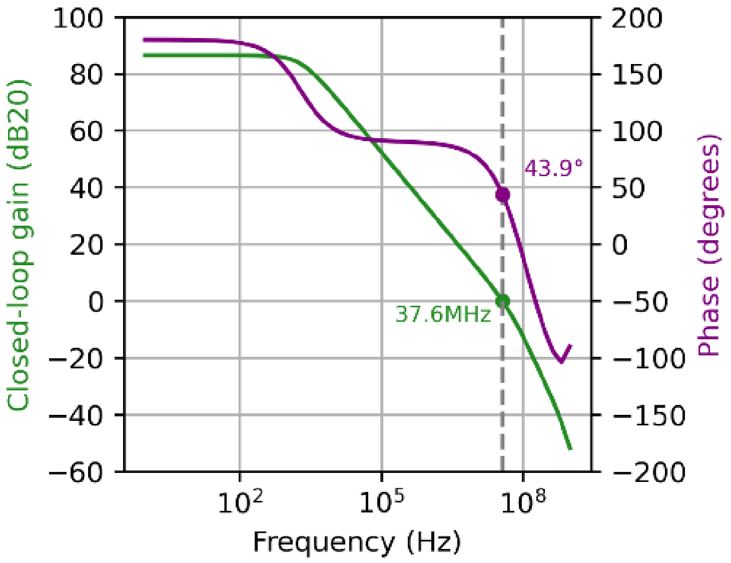

Fig. S8: Operational amplifier (OPA) characteristics, including closed-loop gain and phase margin.

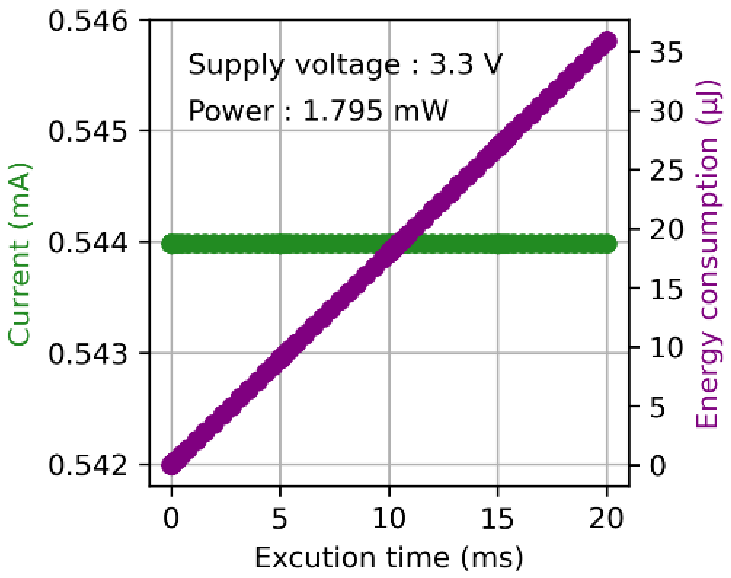

Fig. S9: OPA quiescent current and energy consumption over time.

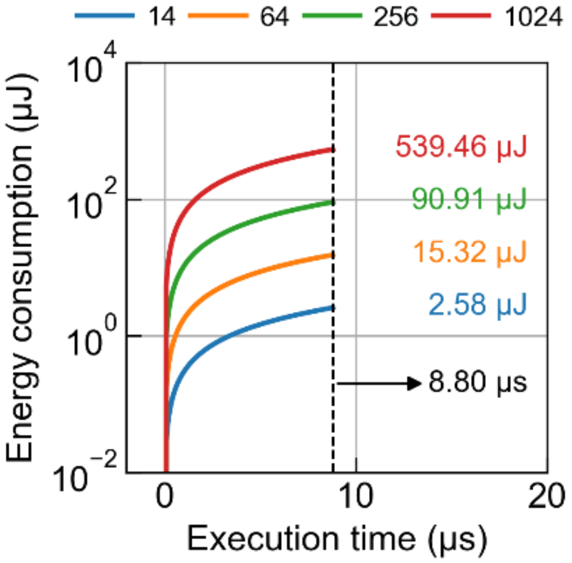

Fig. S10: The energy consumption and execution time of our analog system using 180nm technology node memristor in the Digital Twin of the HP coupled variable-resistor model.

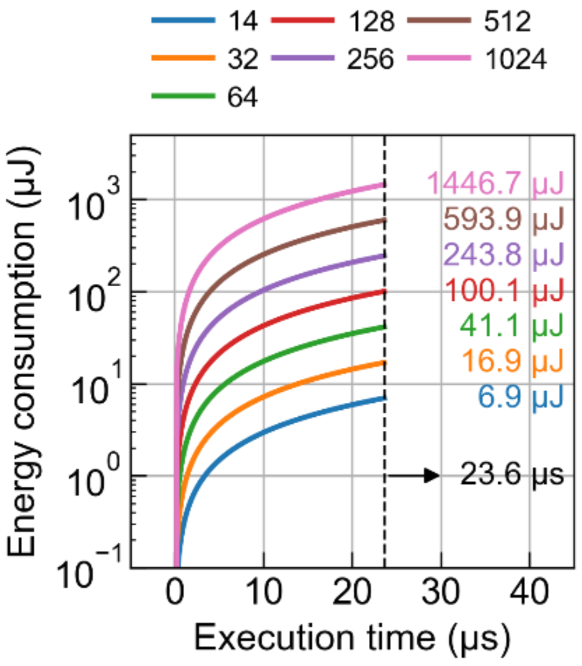

Fig. S11: The energy consumption and execution time of our analog system using 180nm technology node memristor in the Digital Twin of the Lorenz96 model.

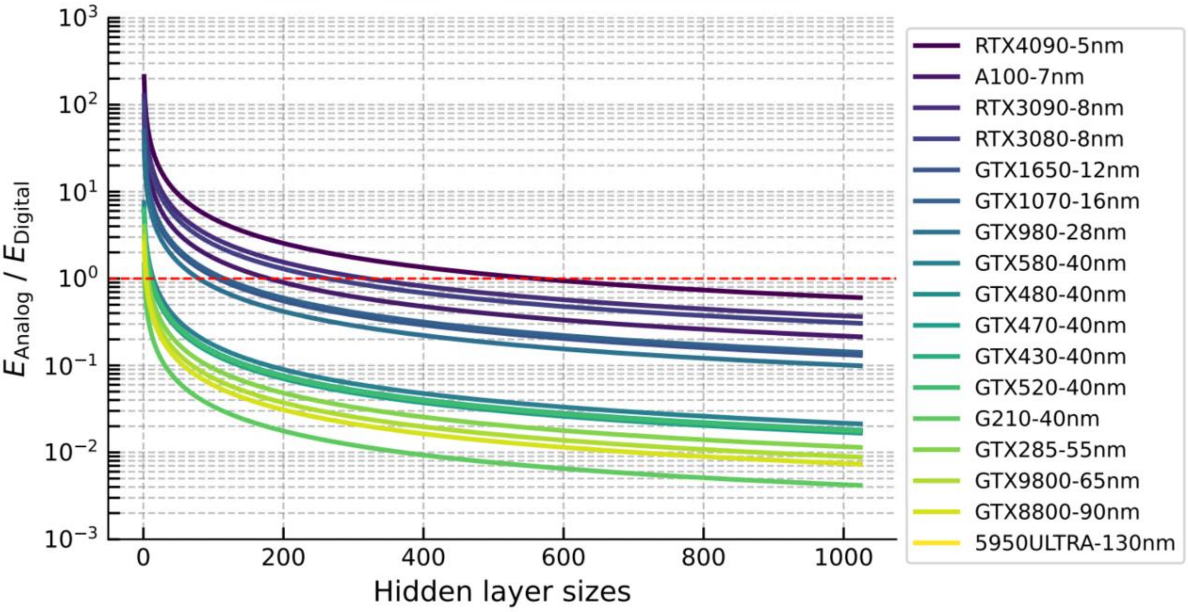

Fig. S12: Energy efficiency ratio of the analog ODE solver and its digital counterparts across varying hidden layer sizes.

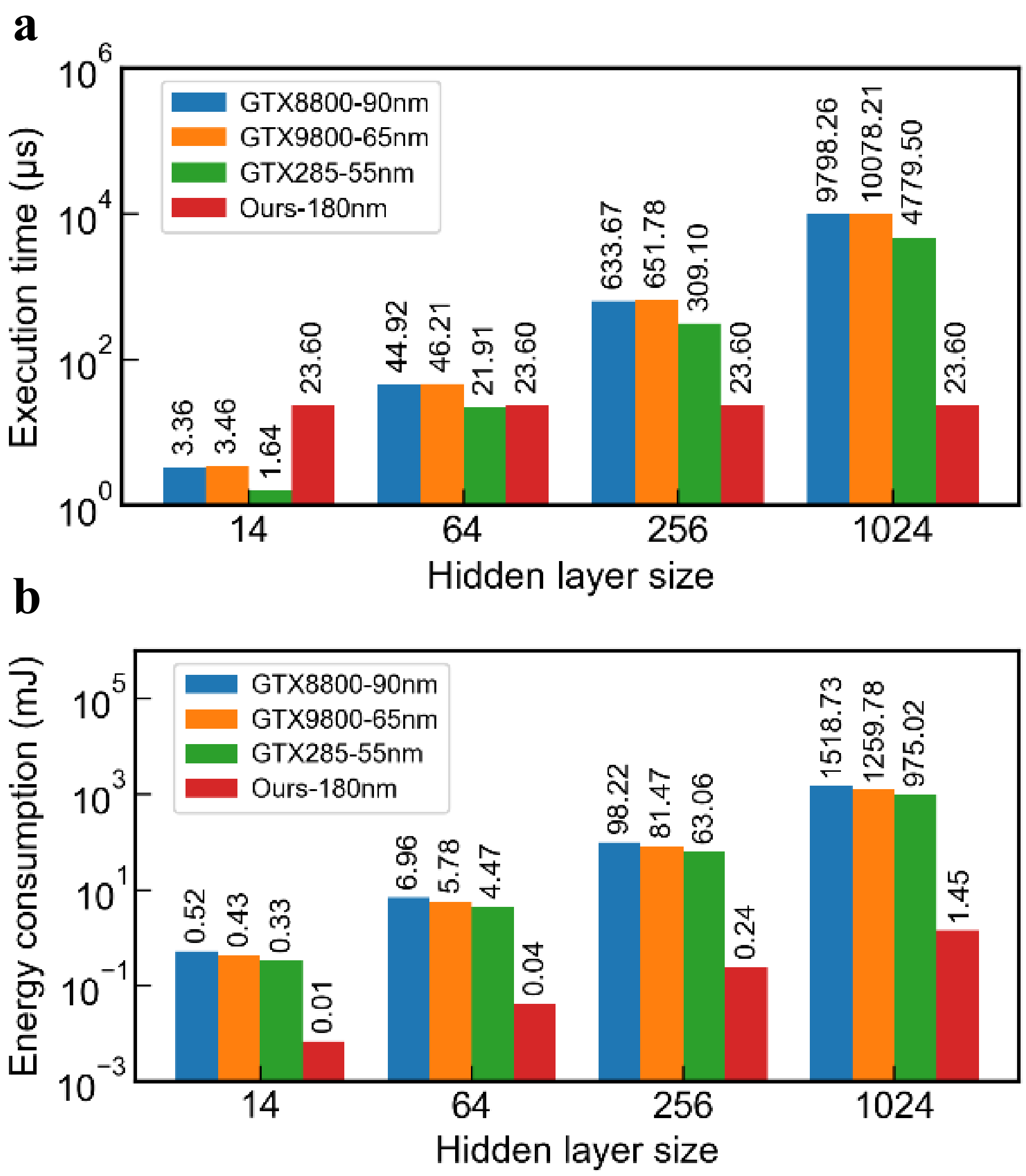

Fig. S13: Neural ODE performance on digital and analog hardware. Comparison of **a,** execution time and **b,** energy consumption for the Neural ODE on different digital hardware platforms, including the GTX 8800, GTX 9800, and GTX 285 with advanced process sizes, as well as the Neural ODE on our analog system with a 180 nm RRAM, executing an extrapolation task across varying hidden layer sizes.

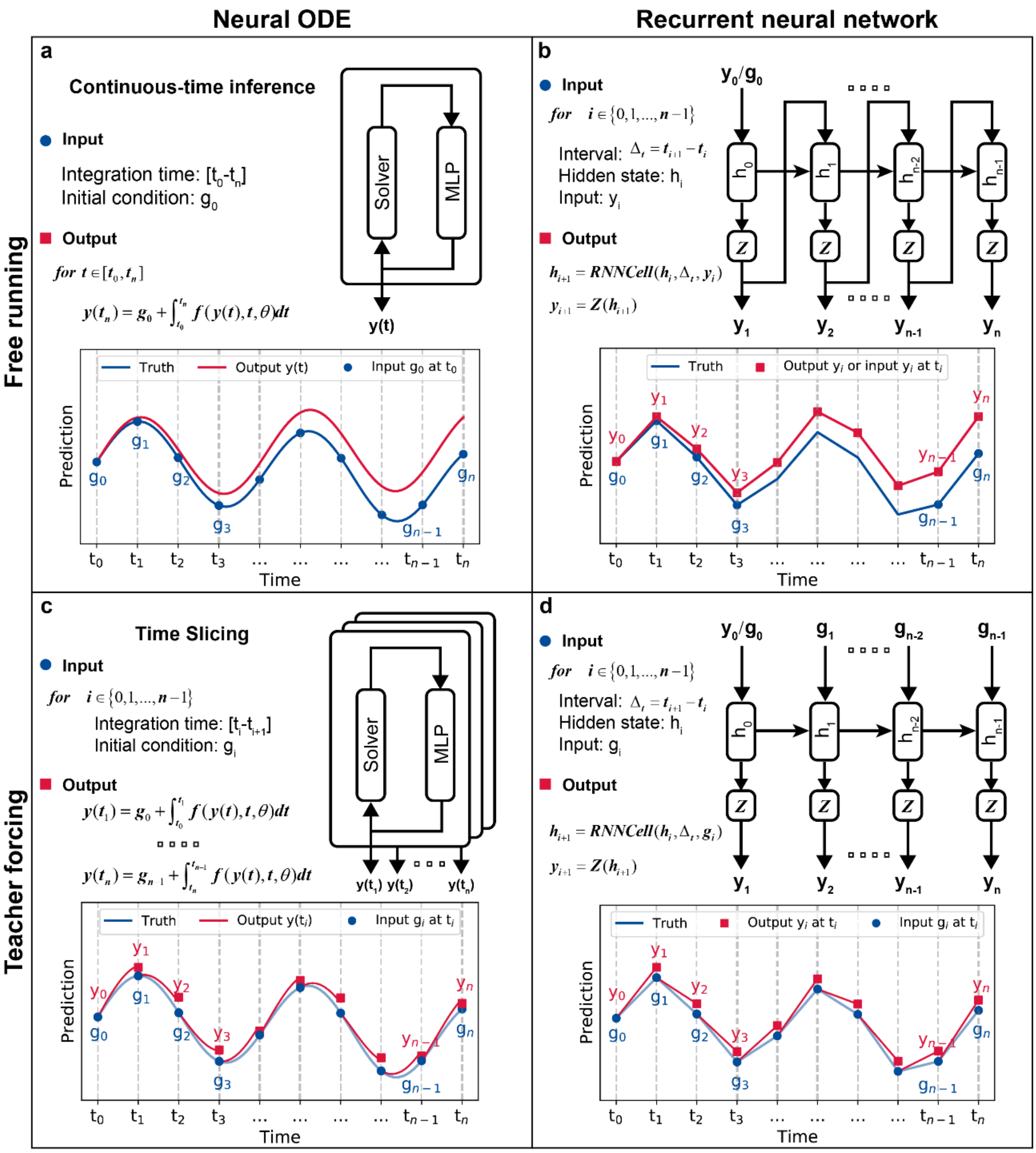

Fig. S14: Times series prediction of Neural ODE and RNN in free running and teacher forcing modes.

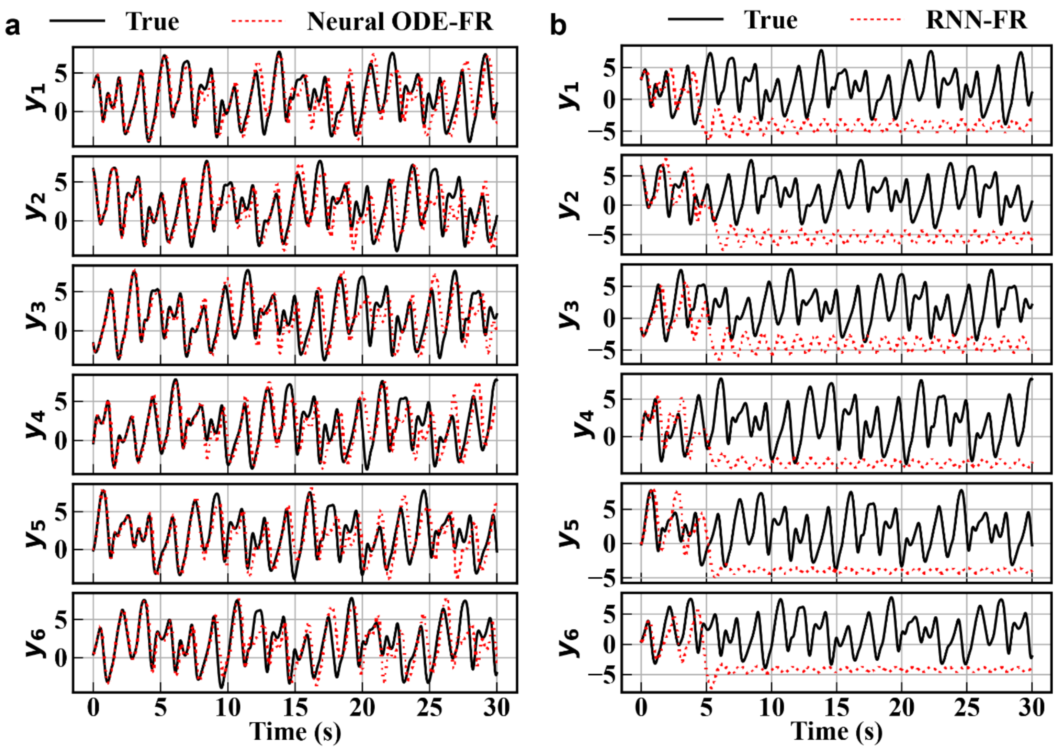

Fig. S15: Predicted trajectories of the Lorenz96. a, Neural ODE and **b,** RNN under free running mode.

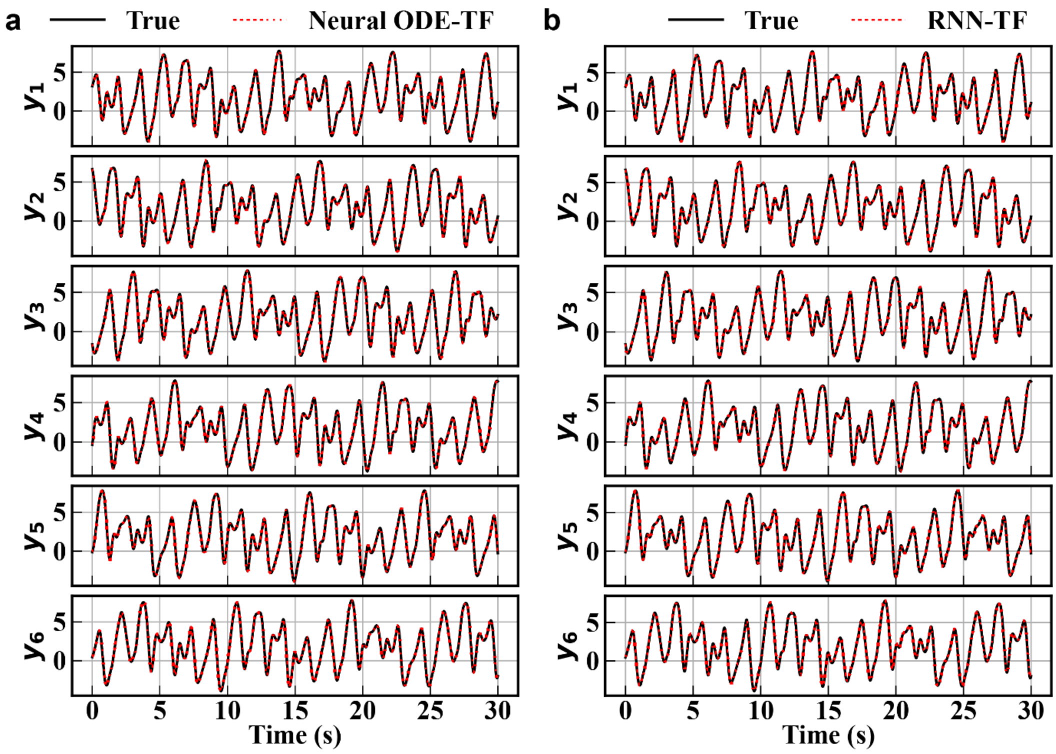

Fig. S16: Predicted trajectories of the Lorenz96. a, Neural ODE and **b,** RNN under teacher forcing mode.

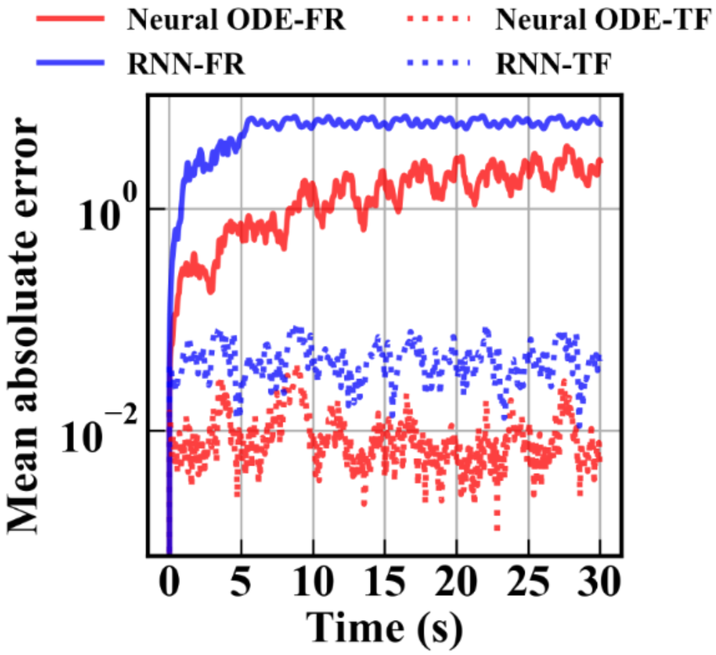

Fig. S17: Instantaneous MAE of Neural ODE and RNN under teacher forcing and free running modes over a 30-second period.

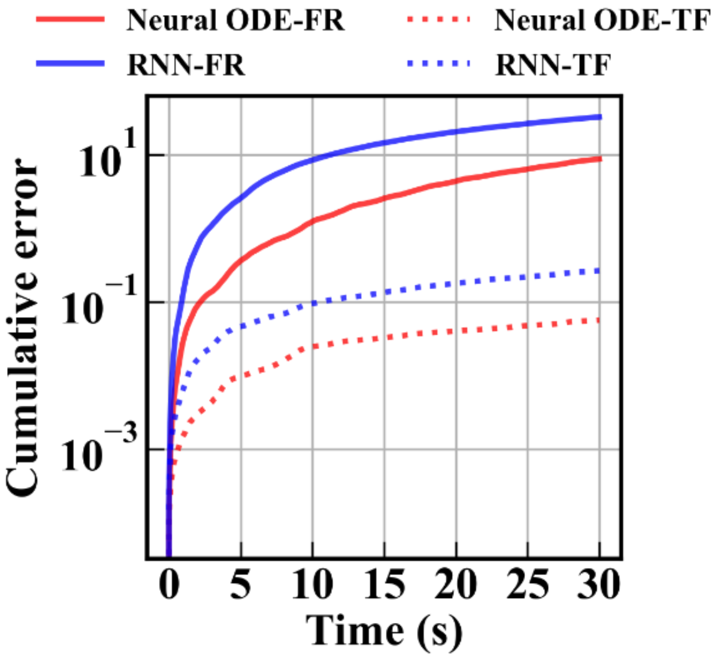

Fig. S18: Comparison of cumulative MAE for Neural ODE and RNN under teacher forcing and free running mode.

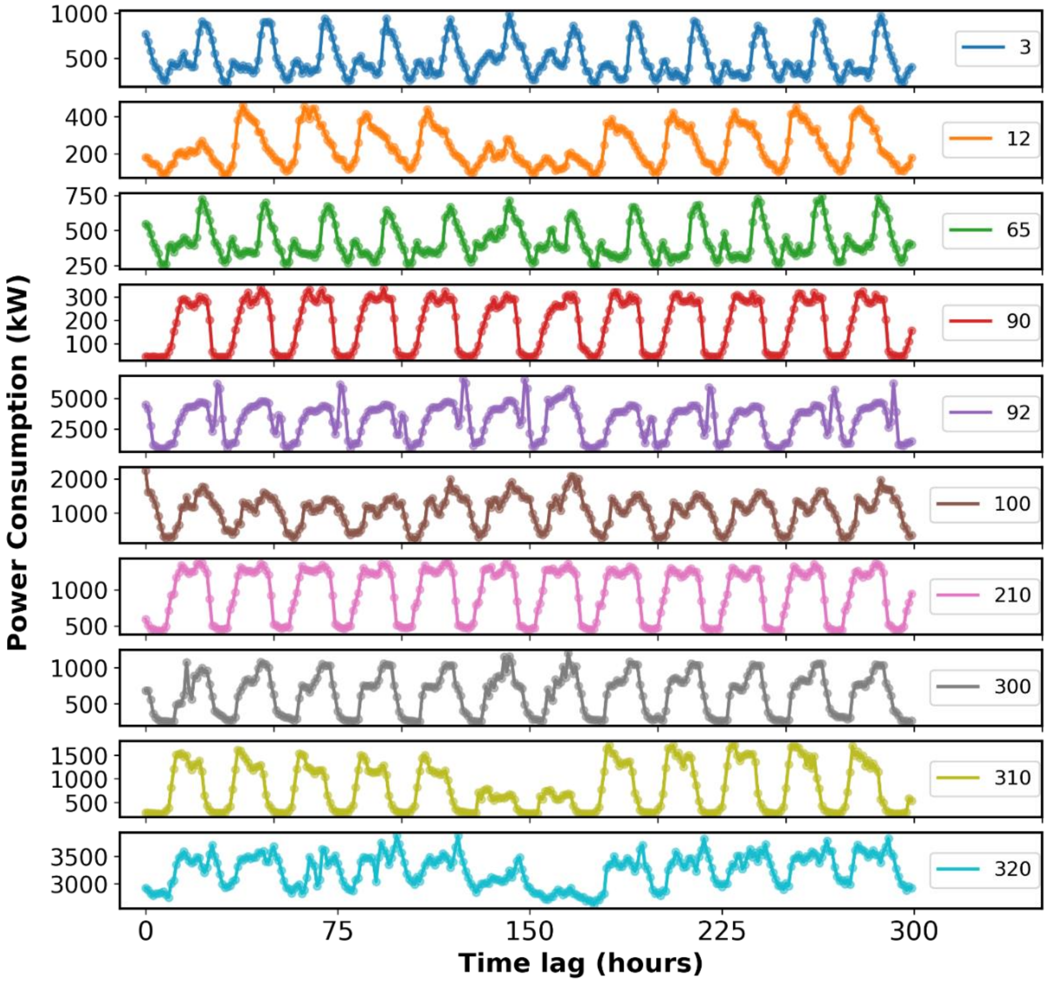

Fig. S19: Visualization of selected dimensions from *Electricity* dataset, representing hourly consumption of a subset of clients over a 300-hour period.

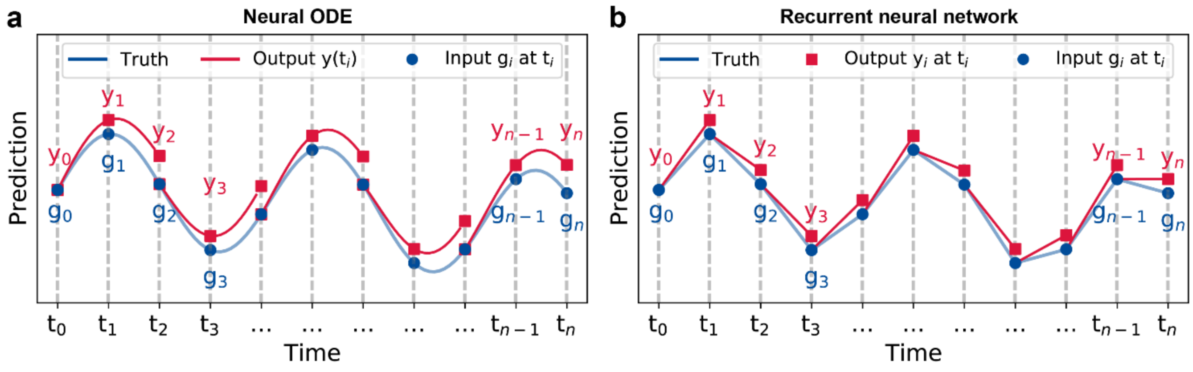

Fig. S20: Contrasting teacher forcing in continuous versus discrete models. a, the Neural ODE employs a segment-to-segment teacher forcing method, while **b,** the RNN utilizes a point-to-point teacher forcing method.

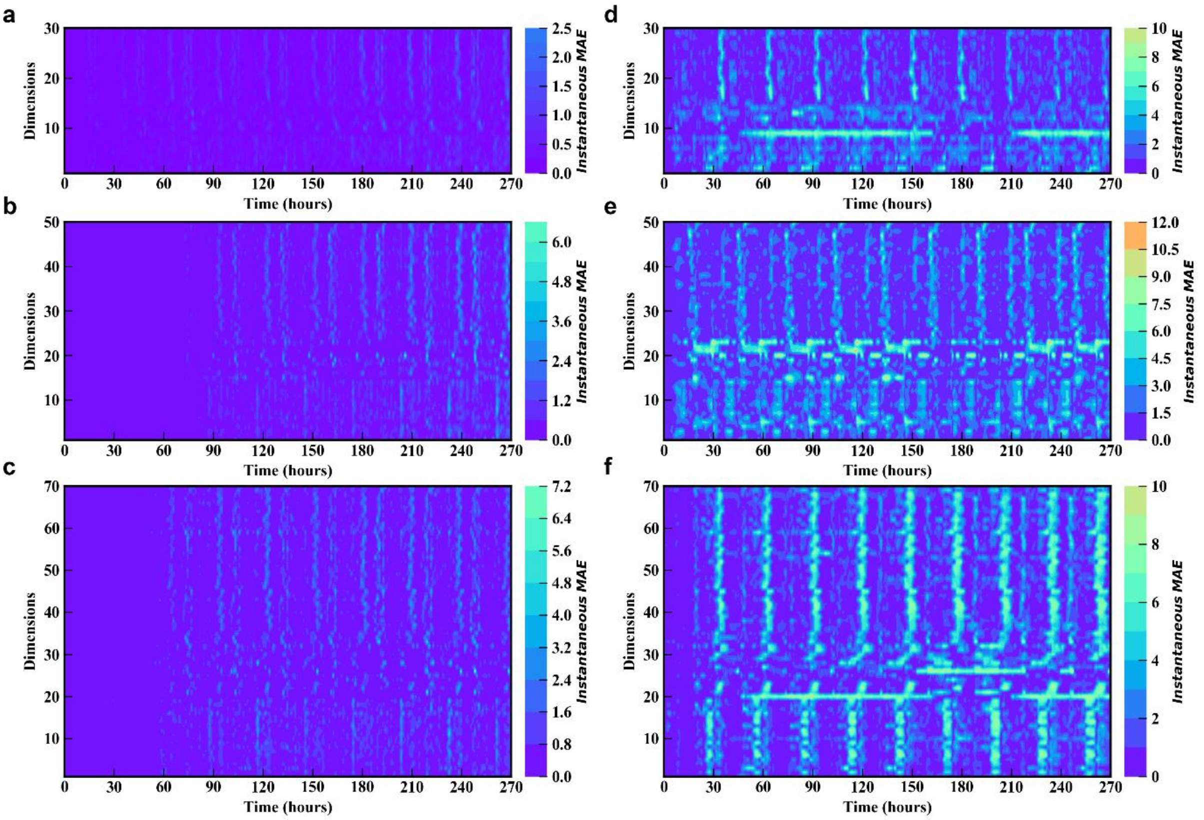

Fig. S21: Interpolation result during training process of **a-c,** Neural ODE and **d-f,** RNN compared to the true trajectory in the real *Electricity* dataset with 30, 50 and 70 dimensions.

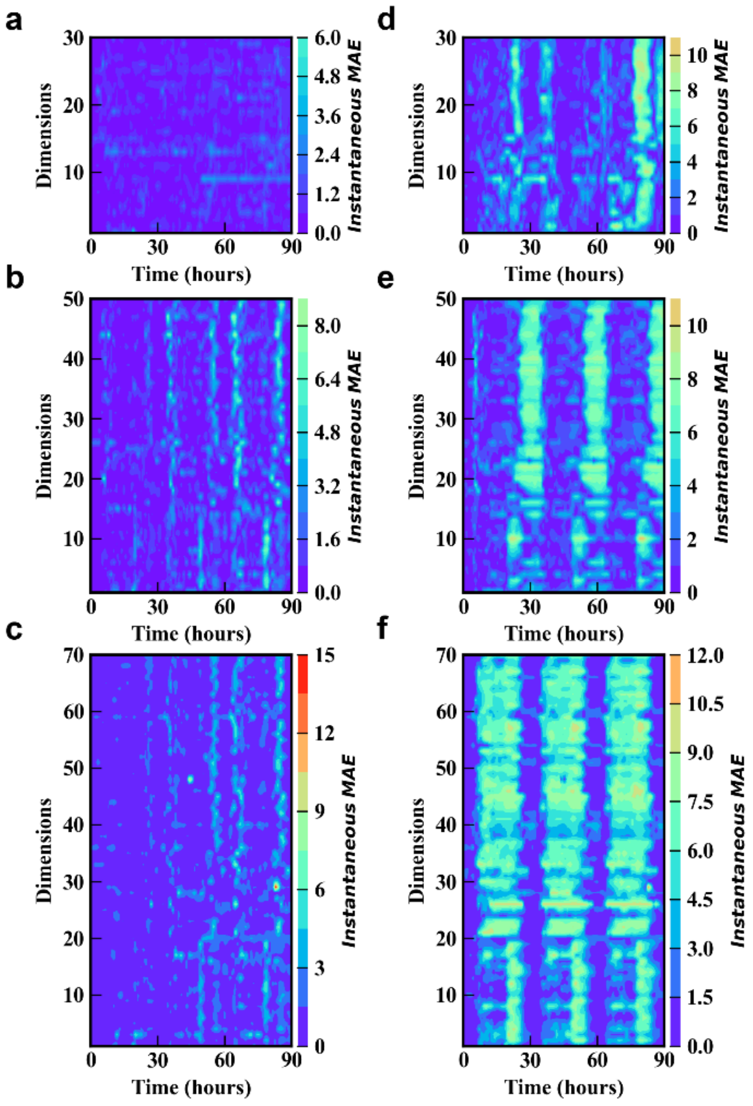

Fig. S22: Extrapolation error during inference process of **a-c,** Neural ODE and **d-f,** RNN for the *Electricity* dataset with 30, 50 and 70 dimensions.

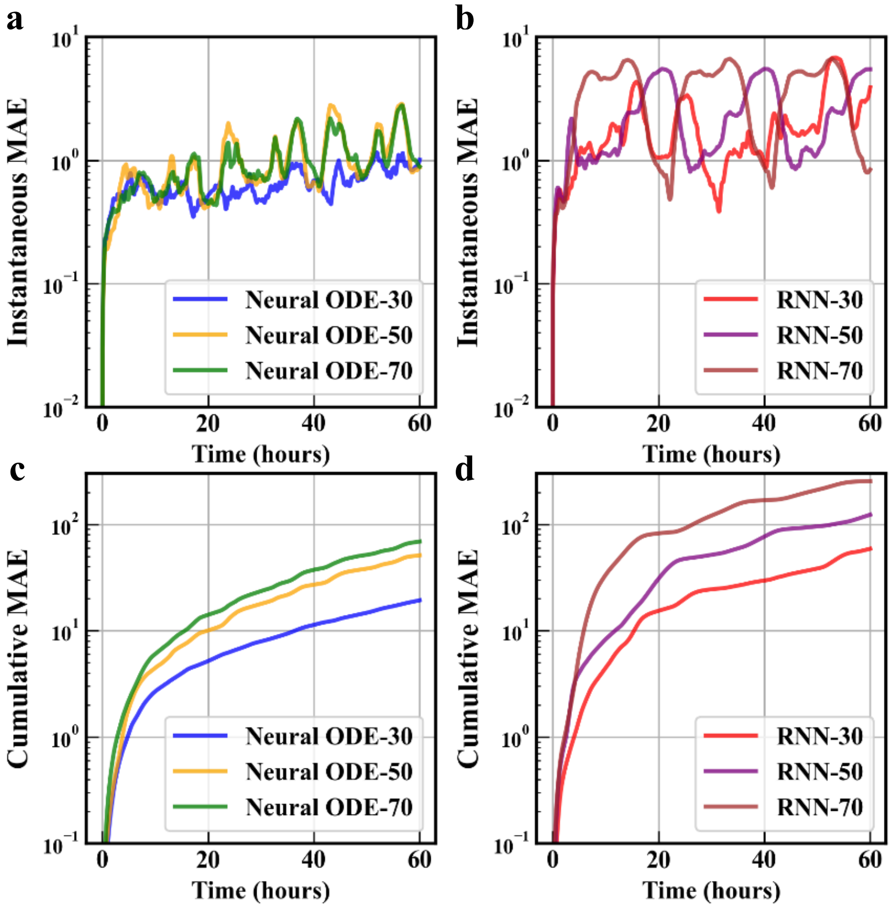

Fig. S23: Result analysis of Neural ODE on the *Electricity* dataset. **a-b,** Instantaneous MAE and **c-d,** cumulative MAE of Neural ODE and RNN for extrapolating 30, 50 and 70 dimensional *Electricity* dataset.

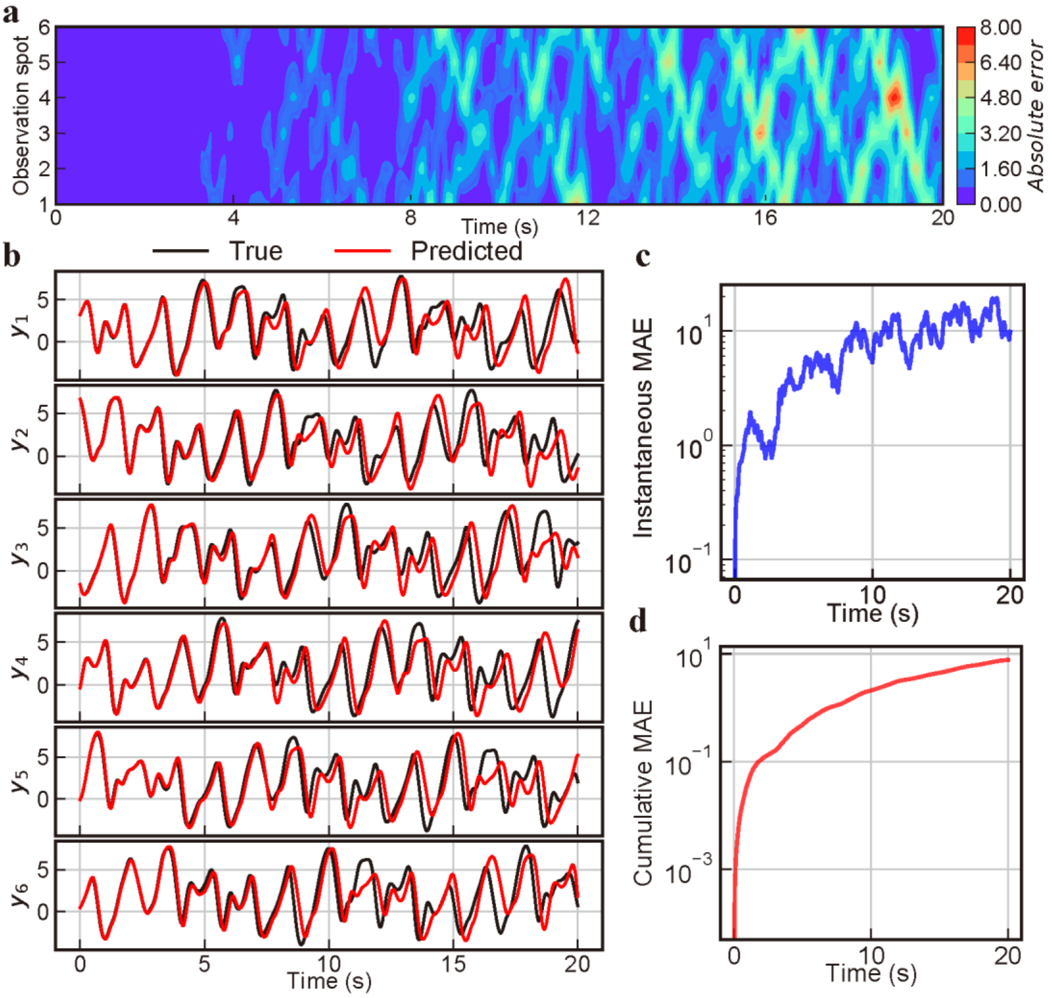

Fig. S24: Error analysis of extrapolation performance for our digital twin. a, Contour plots illustrate the temporal evolution of prediction error across six dimensions with our digital twin over a 20-second sequence. **b**, The comparison between the extrapolated training output generated by our digital twin (red curve) and the ground truth (black curve). **c**, Instantaneous MAE for six channels over a 20-second period. **d**, Cumulative MAE for six channels over a 20-second period.

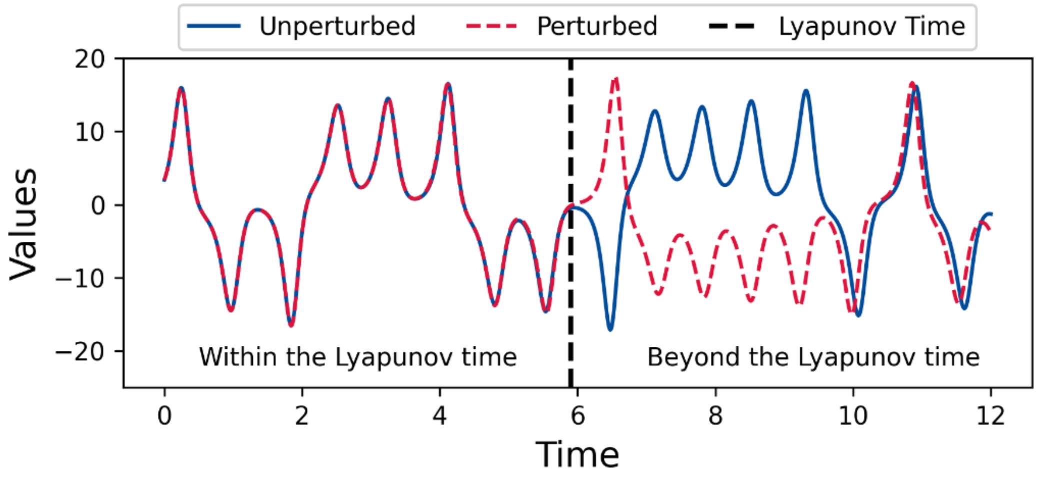

Fig. S25: An example of two trajectories (unperturbed and perturbed) of one of the variables of the Lorenz63 system, with an initial difference of 10^{-8}. Both trajectories remain close within the Lyapunov time (indicated by the dashed vertical line), beyond which they diverge rapidly.

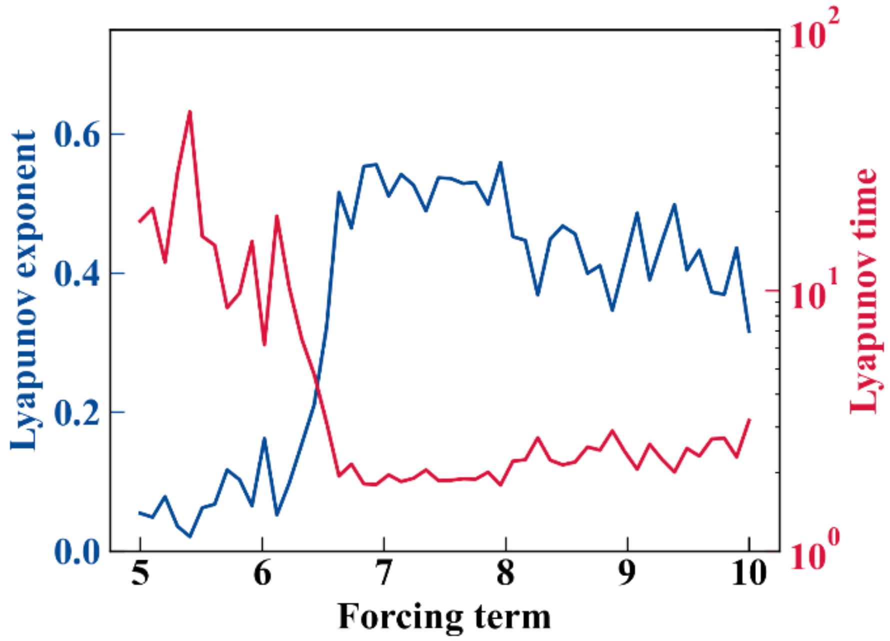

Fig. S26: Lyapunov exponent and lyapunov time of the Lorenz96 dynamics under increasing forcing term *F*.

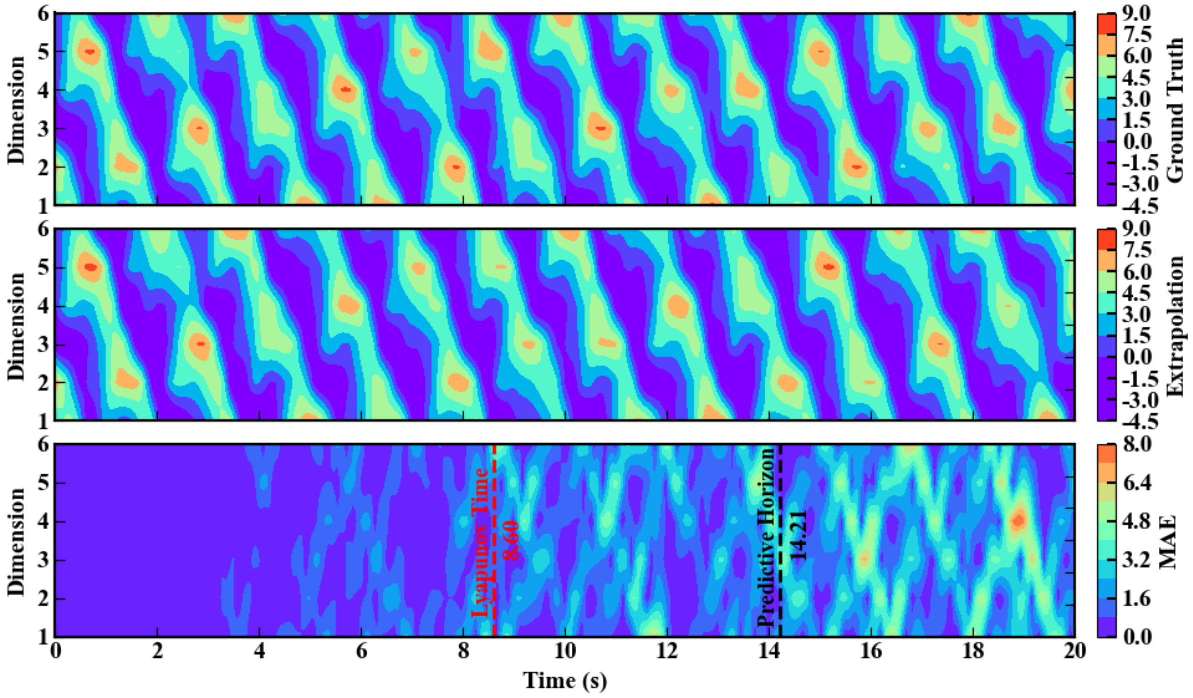

Fig. S27: Extrapolation performance of Neural ODE over time compared with the ground truth. The top panel: the ground truth of the Lorenz96 system with a forcing term $F = 6$ and a dimension $N = 6$. The middle panel shows the extrapolated trajectory generated by the Neural ODE over a 20-second interval. The bottom panel presents the MAE of Neural ODE. The red vertical line represents the Lyapunov time (8.60 s), while the black vertical line indicates the Neural ODE's prediction horizon, extending up to 14.21 seconds.

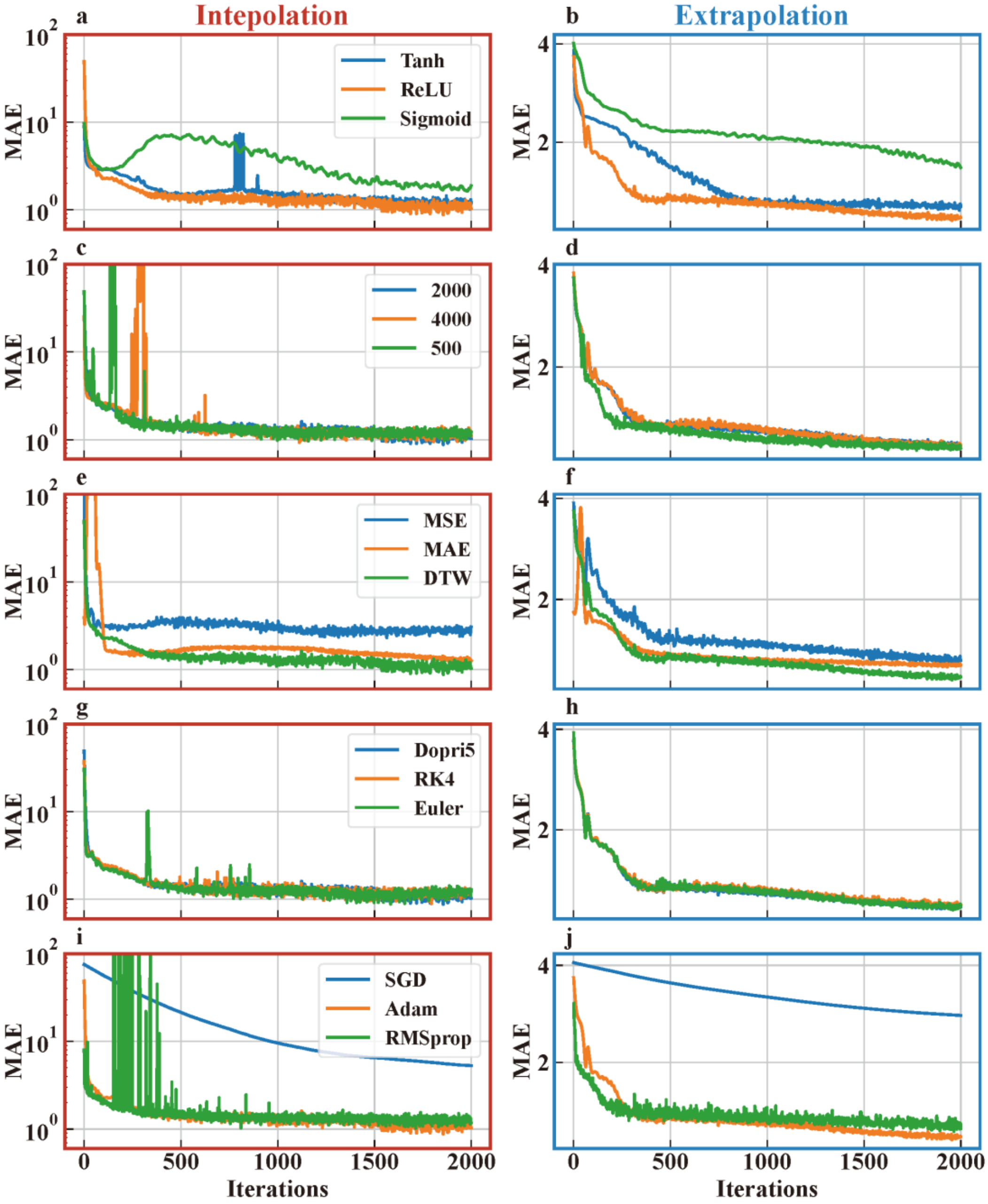

Fig. S28: The training performance of our digital twin for interpolation/extrapolation tasks under different training conditions. The MAE loss function is employed to consistently assess the error across both interpolation (red) and extrapolation (blue) tasks. This evaluation is conducted with respect to **a-b,** various activation functions including Tanh, ReLU and Sigmoid, **c-d,** different sampling points of the training trajectory including 500, 2000, and 4000, **e-f,** various loss functions including MSE, MAE and DTW, **g-h,** different ODE methods including Euler, RK4 and Dopri5, and **i-j,** various optimization algorithms including Adam, SGD and RMSprop.

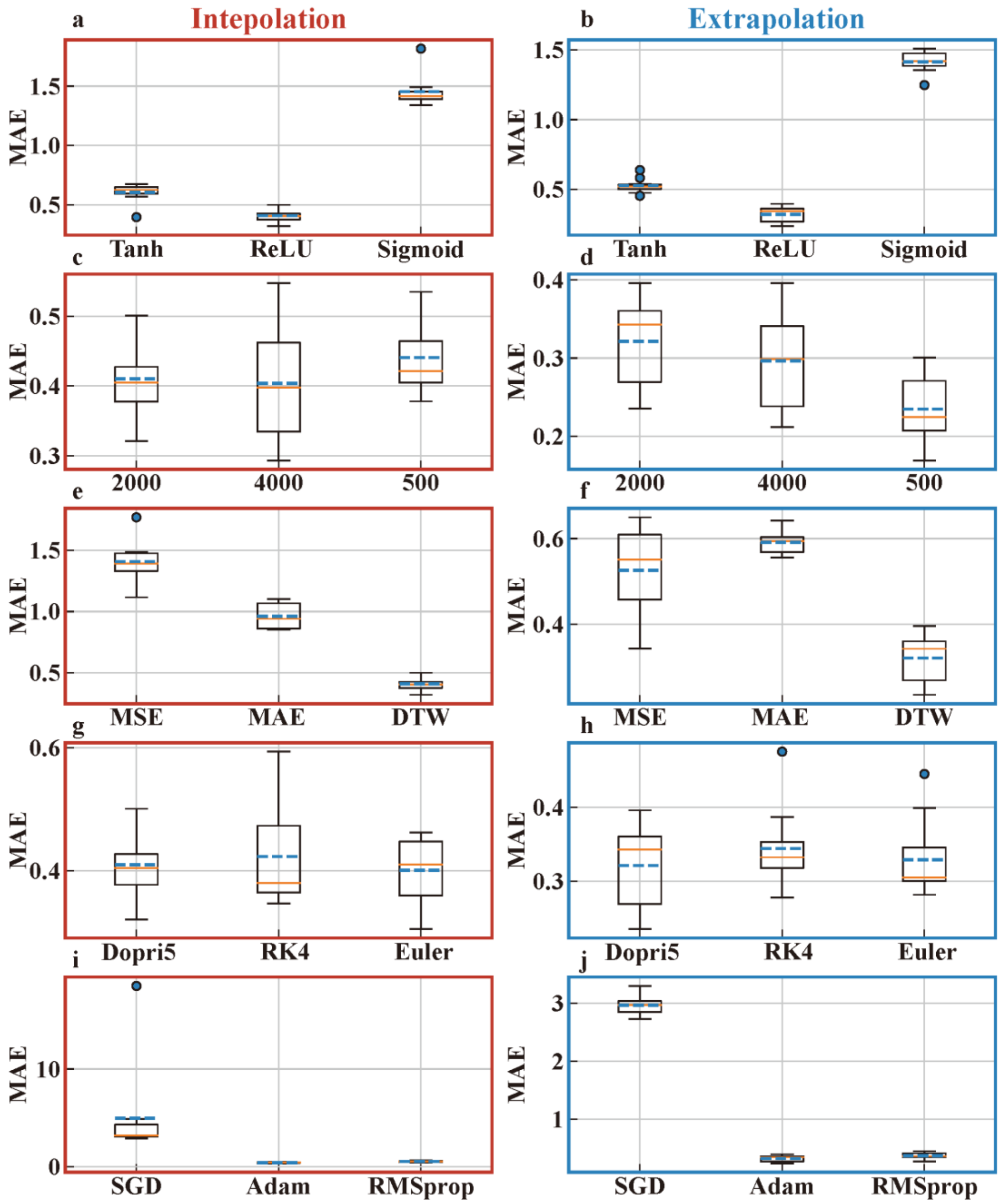

Fig. S29: Error distribution of interpolation/extrapolation error under different training conditions was assessed in this study. We examined the impact of various factors, including **a-b,** activation functions utilized for non-linear transformation, **c-d,** sampling points of the training trajectory used for dataset construction, **e-f,** different loss functions employed for network updates, **g-h,** various ODE methods for solving the ordinary differential equation, and **i-j,** optimization algorithms utilized during the training process. For each depicted box, the data were obtained from a 10-fold cross-validation with 10 trial points. The boxes represent the interquartile range (IQR), where the red line indicates the median and the green triangle represents the mean.

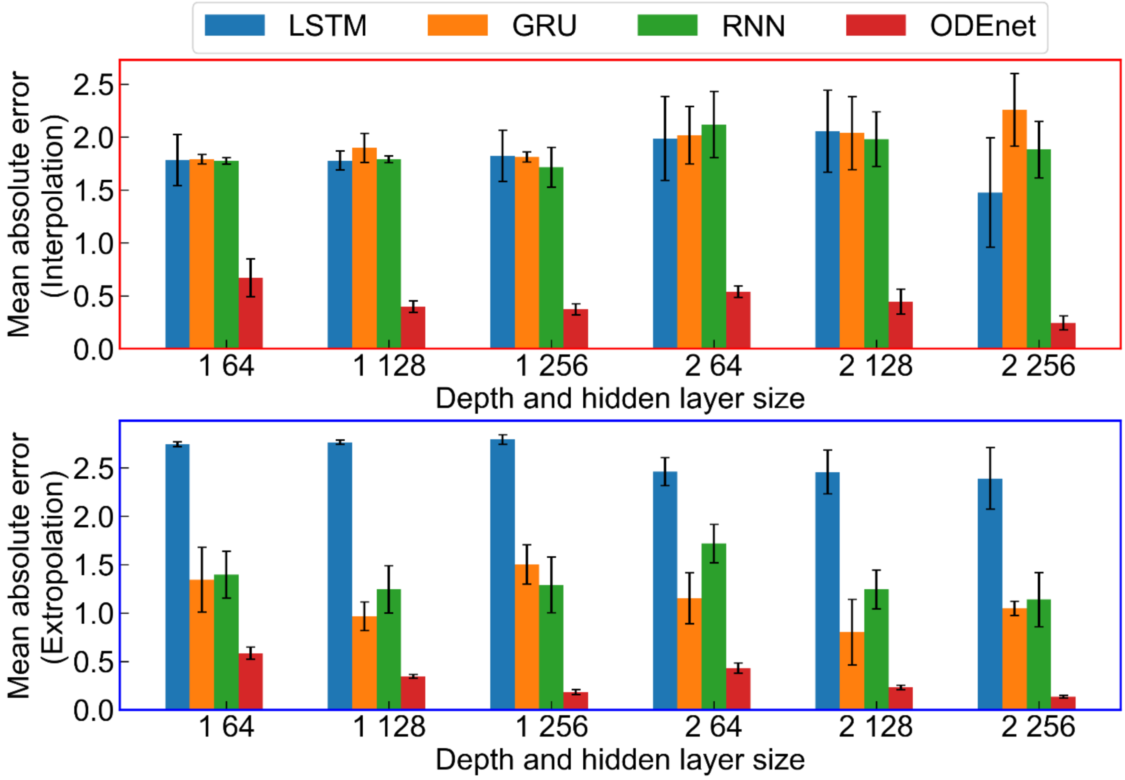

Fig. S30: Performance comparison of traditional models and our digital twin across varying depths and hidden layer sizes. for **a,** interpolation task (red) and **b,** extrapolation task (blue).

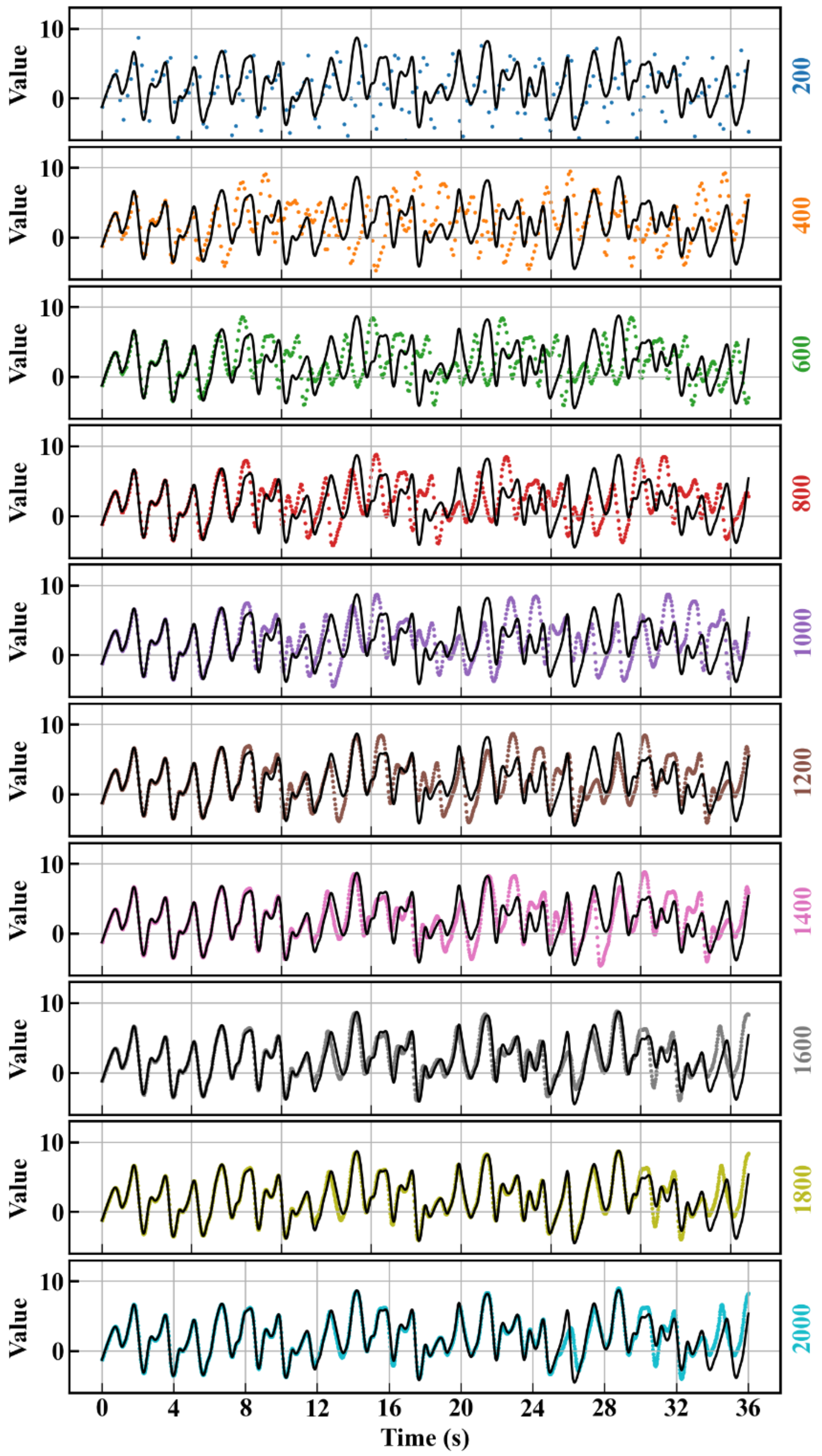

Fig. S31: Interpolation visualization of the first dimension of the Lorenz96 system with varying solution steps applied to the trained Neural ODE using the RK4 solver. The numbers on the right correspond to the respective solution steps.

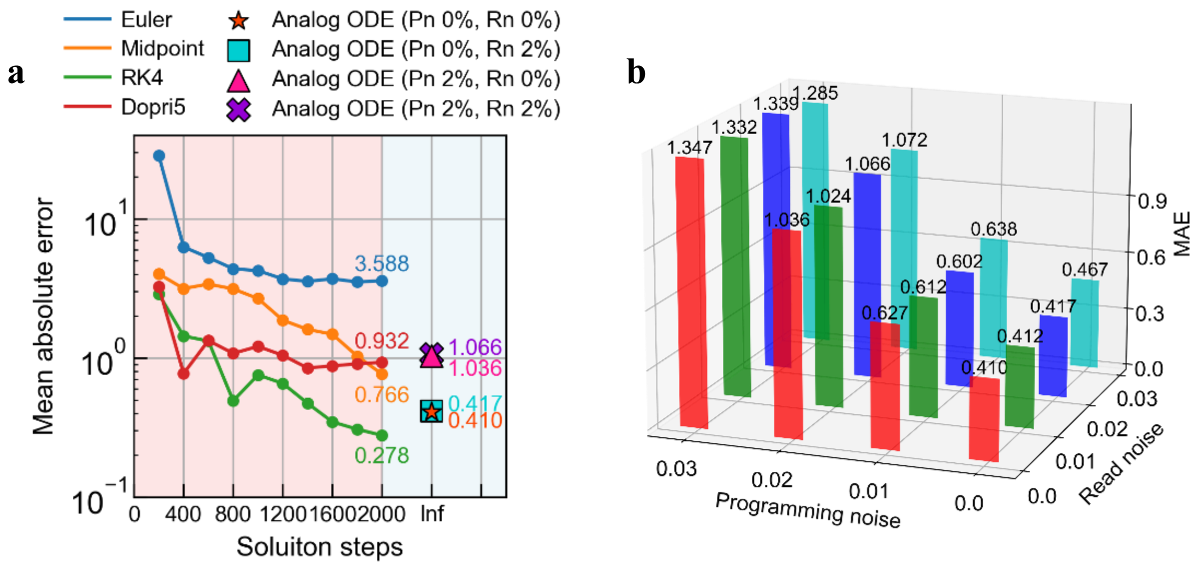

Fig. S32: Accuracy evaluation of analog ODE solver and digital ODE solvers under different conditions. a, Comparison of MAE between digital ODE solvers (Euler, Midpoint, RK4, Dopri5) of different time steps and our analog ODE solver for Lorenze96 dynamics. **b,** Interpolation result of our analog ODE solver with the inclusion of programming noise and read noise.

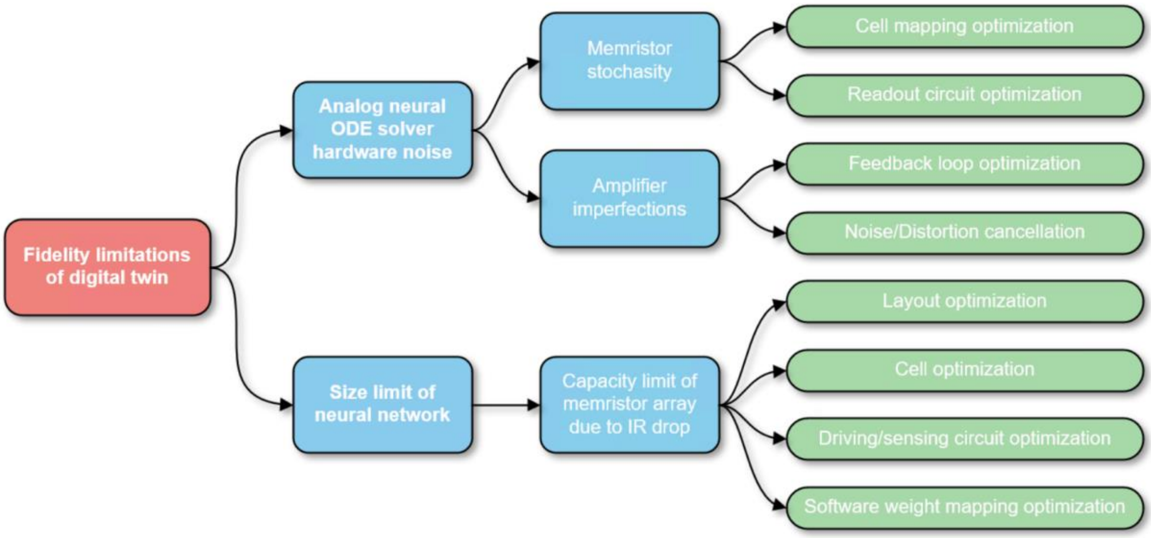

Fig. S33: Fidelity limitations of digital twin using analog neural ODE solver, and prospective strategies for their mitigation.

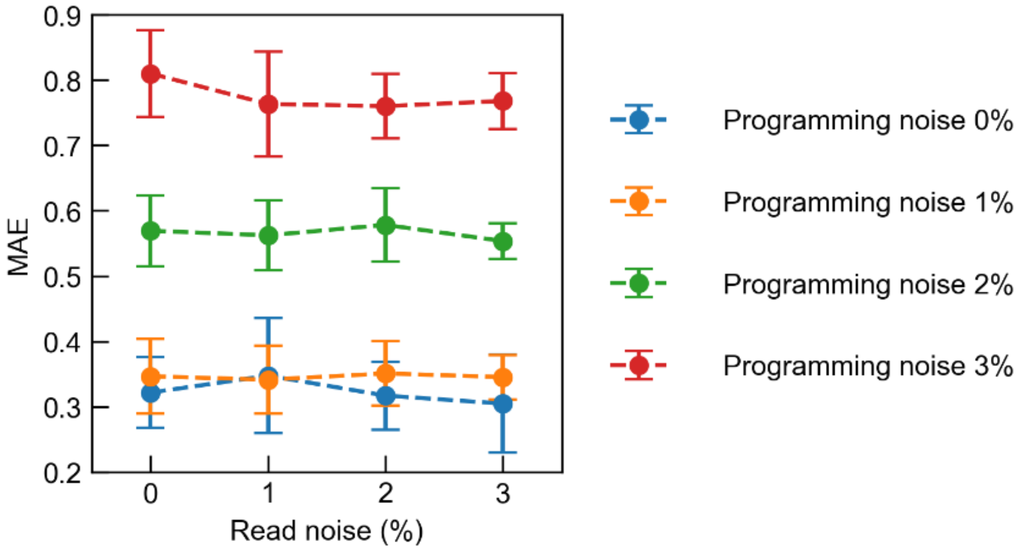

Fig. S34: Error bar plot of MAE for analog Neural ODE solver under varying programming noise and read noises. Each error bar corresponds to 10 measurements.

Supplementary Table 1: Summary of experimental and simulation results.

	Experiments	Simulations
**System**	Fig 2c: IVP integrator[1]	Fig 2c: Simulated IVP integrator's waveform[2]
	Fig 2d: Peripheral circuit[1]	-
	Fig 3c-d: Individual memristors[1]	-
	Fig 3e-f: Memristor array[1]	-
**HP coupled variable-resistor model**	Fig 4c-e: Programmed conductance on memristor arrays[1]	-
	Fig 4f: Experimental input and output waveforms[1] (Hidden layer size = 14)	Fig 4f: Simulated input and output waveforms[2]
	Fig 4g-i: Experimental hidden neuron waveforms[1] (Hidden layer size = 14)	-
	Fig 4j: Error estimation of experimental waveforms[1] (Hidden layer size = 14)	Fig 4j: Error estimation of Recurrent ResNet[2] (Hidden layer size = 14)
	-	Fig 4k-l: Speed and energy estimation of Recurrent ResNet[3], Neural ODE[3] and ours[2] (Hidden layer size = 14, 64, 256 and 1024)
**Lorenz96 dynamics**	-	Fig 5d-f: Interpolation and extrapolation tasks with ours[2,3]
		Fig 5g-i: Error, speed and energy estimation of LSTM[3], GRU[3], RNN[3] and ours[2] (Hidden layer size = 14 32, 64, 128, 256, 512 and 1024)
		Fig 5j: Noise analysis of our approach[2] (Hidden layer size = 512)

[1]Our system, [2]Candance Virtuoso, [3]NVIDIA GeForce GTX 285

Supplementary Table 2: Comparative computational breakdown of energy formulations in analog neural ODE and digital Neural ODE.

	**Analog Neural ODE**		**Digital Neural ODE**
	Memristor energy	OPA energy	MAC (FP32) energy
**First layer**	$2E_{memristor} \times d_{in} \times N$	$2E_{OPA} \times N$	$2E_{MAC} \times d_{in} \times N$
**Middle layers**	$2E_{memristor} \times depth \times N^2$	$2E_{OPA} \times depth \times N$	$2E_{MAC} \times depth \times N^2$
**Last layer**	$2E_{memristor} \times d_{out} \times N$	$2E_{OPA} \times d_{out}$	$2E_{MAC} \times d_{out} \times N$

Supplementary Table 3: Architecture comparison of RNN and Neural ODE models.

	RNN	Neural ODE
**Input Size**	N (30, 50 and 70)	
**Hidden Size**	512	
**Number of Layers**	4	
**Activation**	Tanh	ReLU
**Output Size**	N (30, 50 and 70)	
**Training mode**	Teacher forcing	
**Inference mode**	Free running	
**Solver**	Not applicable	RK4
**Key feature**	Handles temporal sequences	Model continuous dynamics

Supplementary Table 4: Lyapunov time T_L and corresponding prediction horizon t_{PH} of Neural ODE for Lorenz96 extrapolation.

**Lorenz 96 dynamics**		**Neural ODE**	
**F**	**Lyapunov time T_L (s)**	**Prediction horizon t_{PH} (s)**	**Ratio between Lyapunov time and prediction horizon t_{PH}/T_L**
**6**	8.60	14.21	1.65
**6.5**	3.26	9.09	2.79
**7**	1.83	6.8	3.78
**7.5**	2.08	4.3	2.07
**8**	2.22	3.7	1.67

Supplementary Table 5: Comparison between four digital solvers and our analog solver.

Solver		Computational Complexity	Solution Step	Precision
Digital	**Euler**	Low	Finite and fixed step	First-order accuracy
	**Midpoint**	Slightly higher than Euler		Second-order accuracy
	**RK4**	Moderate		Fourth-order accuracy
	**Dopri5**	Higher due to adaptivity	Finite and adaptive step	Fifth-order accuracy
**Analog (CIM)**	**Ours**	Low	Infinite step	Limited by programming error

Supplementary Table 6: Evaluating the model classification performance under multiplicative noise and testing time noise, Table 1 from Ref. [61].

Data	Accuracy@1 — w/o TTN				Accuracy@1 — w/ TTN			
	ODE	Additive	Multiplicative	Dropout	ODE	Additive	Multiplicative	Dropout
CIFAR-10	87.95	88.69	89.06	88.23	–	88.73	**89.77**	88.44
CIFAR-10.1	70.00	70.80	71.50	71.85	–	71.70	72.05	**73.60**
STL-10	58.03	61.23	60.54	61.26	–	62.11	**62.58**	62.13
Tiny-ImageNet	45.19	45.25	46.94	47.04	–	45.39	46.65	**47.81**

Supplementary Note 1 | Summary of simulation and experimental results.

We have included **Supplementary Table 1**, which lists experimental and simulation results in each sub-figure.

1. Hardware system and memristor array: All results related to the circuit blocks (**Figure 2** of the main text) and the memristor array (**Figure 3** of the main text) are experimental. The corresponding Cadence simulation of IVP integrator is based on software.

2. HP-coupled variable-resistor model: In **Figure 4** of the main text, the programmed conductance on the memristor arrays, as well as the input, hidden, and output waveforms of our analog solver, are experimental. The corresponding Cadence simulated waveforms are based on software. Additionally, we analytically estimated the speed and energy consumption on the GTX 285 GPU (Recurrent ResNet, Neural ODE), and simulated those of analog ODE solver using Cadence Virtuoso.

3. Digital twin of Lorenz96 dynamics model: In **Figure 5** of the main text, we simulated the digital twin on the analog and digital hardware, as the model size is much larger than the capacity of the memristor array [62].

Supplementary Note 2 | Energy consumption and execution time compared with latest GPU.

To ensure a fair comparison, we now directly compare the performance of our system with GPUs of similar (but still more advanced) technology nodes, such as the Nvidia GTX 8800, GTX 9800 and GTX 285. No voltage scaling is used in the comparison. The energy consumption and execution time of our analog system was measured on Cadence Virtuoso using UMC 180nm technology node. For the two tasks, the improvements in speedup and energy efficiency compared to the Nvidia GTX 285 are as follows:

- For the Digital Twin of the HP coupled variable-resistor model, we achieve **134.9**× improvement in speeds and **449.0**× energy efficiency improvements.
- For the Digital Twin of Lorenz96 model, we achieve **202.5**× improvement in speed and **673.9**× energy efficiency improvements.

Furthermore, we have conducted additional experiments using *torch.profiler* [63, 64] to directly measure the GPU execution time of baseline models on an NVIDIA GeForce RTX 4090. Our results demonstrate **166.5**× and **369.3**× improvement in speeds for the Digital Twin of the HP coupled variable-resistor model and the Digital Twin of Lorenz96 model, respectively.

This response is organized as follows: ***Part 1*** introduces how energy consumption and speed are calculated in digital systems. ***Part 2*** details how energy consumption and execution time are measured in our analog system. ***Part 3*** compares the power consumption and execution time between our analog system and digital system on the two tasks. ***Part 4*** provides the analysis of the energy advantage between our analog system and digital system.

1. Performance estimation of digital systems: In this paper, we use an Nvidia GTX 285 (TSMC 55 nm) to benchmark the performance of digital computing system in implementing the Recurrent ResNet, Neural ODE, LSTM, GRU and simple RNN.

1.1 Calculating energy consumptions: We use a simple but effective method to estimate energy consumption [65-67], that is to measure the energy efficiency ($E_{efficiency}$, for FP32 data) and number of multiply-and-accumulate (MAC) operations (Ops). Here, Ops represents the total number of operations required in a digital twin task. Therefore, the corresponding energy consumption of the digital system can be expressed as:

$$E_{digital} = Ops * E_{efficiency} \tag{S1}$$

Here energy efficiency is estimated using the peak throughput (708.5 GOPS, FP32 data) and TDP (Thermal Design Power, 204W).

$$E_{efficiency} = TDP/Throughput \tag{S2}$$

1.2 Calculating execution time via GPU peak throughput: We calculate the total execution time by measuring the number of Ops and dividing it by the peak GPU's throughput:

$$T_{digital} = Ops/Throughput \text{ (by tech node and chip area)} \tag{S3}$$

As shown in **Fig. S7**, this estimation represents the most extreme case of execution time, as it does not account for the latency inherent in the sequential nature of time-series prediction tasks.

1.3 Calculating execution time using PyTorch profiling method: PyTorch's built-in profiling tools *torch.profiler* [63, 64] is widely used for precise measurement of CPU and GPU execution times. Here we employ hardware-level profiling via *torch.profiler* (PyTorch v2.1+), as follows:

1) **Multi-stage scheduling configuration**: We used a standard multi-stage scheduling protocol to mitigate transient overheads and attain stable profiling conditions. The first ten iterations were discarded (skip) to eliminate initialization effects, followed by five stabilization iterations (wait). Subsequently, ten warmup iterations primed the GPU for sustained performance, after which performance metrics were collected over 25 active iterations.

2) **Targeted profiler configuration**: We configured our profiler to capture performance metrics strictly associated with GPU computation, thereby excluding CPU-induced latencies and synchronization overhead.

3) **Model loading and execution**: The model was pre-initialized with synthetic (dummy) input data to avoid any confounding effects from initialization overhead. GPU operations were synchronized at each transition (skip → wait → warmup → active).

4) **Computation of average execution time**: Model execution times were aggregated across multiple runs to derive representative kernel execution times. This multi-stage profiling protocol was repeated five times to enhance the robustness and reproducibility of the measurements.

By leveraging the high-resolution measurements obtained via *torch.profiler*, we accurately captured execution times across various model configurations, as shown in **Figure 4k** and **Figure** 5h in the main text, incorporating the results from the built-in GPU timing analysis.

2. Performance estimation of analog system

To estimate the performance of the fully analog system, we performed chip-level simulations using Cadence Virtuoso. In addition, we introduce the core component of the analog system, the IVP integrator in section ***2.1***. We then present the impact of close-loop gain, phase response and quiescent current of IVP OPA on the energy consumption and execution time in section ***2.2***.

2.1 The core hardware component of Neural ODE – IVP integrator: Neural ODEs are formulated as continuous ordinary differential equations, as shown in Eq. (S8). For continuous-time and fully analog circuit implementation using memristors, we designed an initial value problem (IVP) integrator (**Figure 2b** in the main text). Its function is as follows:

$$V_{out} = \begin{cases} 0, & if\ V_{ctrl} < 0.1 \\ -\frac{1}{RC}\int V_{in}dt, & if\ V_{ctrl} \geq 0.1 \end{cases} \tag{S4}$$

where V_{ctrl} is the control voltage used to switch the IVP integrator between the initial conditioning mode and the current integration mode. R and C represent the resistance and capacitance of the integrator, respectively. By adjusting the RC time constant, we can precisely control the execution time of the target task under the constraint of bandwidth.

2.2 Calculating execution time and energy consumptions

The energy consumption for two tasks is measured by monitoring the powers supply and integrating the power over time in Cadence Virtuoso. The execution time of the analog system is obtained directly from the runtime reported by the Cadence Virtuoso.

In terms of energy consumption, the primary source is the static power of the OPA. In addition, OPA's bandwidth directly relates to the execution time. Therefore, we discuss the OPA used in the IVP integrator in terms of closed-loop gain, phase response, and quiescent current.

2.2.1 Closed-loop gain and phase response: The **closed-loop gain** quantifies the amplifier's ability to maintain a stable output relative to its input, expressed as 20 times the logarithm (base 10) of the amplitude ratio (dB). As shown in **Fig. S8**, the amplifier achieves a bandwidth of 37.6 MHz at unity gain, indicating the frequency range over which the gain remains at 1 (0 dB). The phase margin, defined as the difference between the phase angle at unity gain (0 dB) and -180 degrees, is 43.9 degrees. A phase margin between 40 and 60 degrees typically ensures a stable amplifier with a good transient response. Given the gain-bandwidth product (GBW) of 37.6 MHz, this sets a limit on the maximum allowable RC time constant for the system.

2.2.2 Quiescent current: Quiescent current refers to the current consumed by an OPA when it is neither driving a load nor amplifying a signal. This parameter is crucial for evaluating the power efficiency of the amplifier, especially in low-power or battery-operated devices. As shown in **Fig. S9**, the OPA exhibits a quiescent current of 0.13 mA. Using the formula $E = I \times V \times t$, where I is the quiescent current, V is the supply voltage (3.3 V), and t is the system's execution time, the energy consumption of OPA shows a linear increase over time, with a power consumption of 1.795 mW.

3. Energy consumption comparison between analog and digital systems in two digital twin tasks

3.1 Digital twin of the HP coupled variable-resistor model: We employed a network architecture with 2 input neurons and 1 output neuron, varying the hidden layer size across 14, 64, 256, and 1024. For our analog system (**Fig. S10**), the execution time remained constant at 8.8 μs, independent of the hidden layer size, with the system operating at an RC time constant of 1.57×10^{-4}s. Additionally, as the hidden layer size increased, the energy consumption also rose, reaching 2.58 μJ, 15.32 μJ, 90.91 μJ, and 539.46 μJ for 14, 64, 256, and 1024 hidden sizes, respectively.

For the digital system (Nvidia GTX 285), as shown in **Fig. S7a** of the main text, the execution time for the recurrent ResNet and Neural ODE with 100 solution steps increases with the hidden layer size, due to the corresponding increase in the number of FLOPs. When the hidden layer size reaches 256 and 1024, our analog system (8.8 μs) achieves 8.5× and 134.9× improvement in execution time compared to the Neural ODE on the digital system (74.9 μs and 1187.5 μs), respectively.

As illustrated in **Figure 4l** of the main text, both digital and analog systems exhibit increasing power consumption as the size of the hidden layer grows. Notably, the energy consumption ratio between the Neural ODE on analog system and digital system consistently improves, achieving 21.2×, 64.5×, 168.0×, and 449.0× energy efficiency improvements at hidden layer sizes of 14, 64, 256, and 1024, respectively.

3.2 Digital twin of Lorenz96 dynamics: We employed a network architecture with 6 input neurons and 6 output neurons, varying the hidden layer size across 14, 32, 64, 128, 256, 512 and 1024. For our analog system (**Fig. S11**), the execution time remained constant at 23.6 μs, independent of the hidden layer size, with the system operating at an *RC* time constant of 1×10^{-6}s. As the hidden layer size increased, the energy consumption also rose, reaching 6.9 μJ, 16.9 μJ, 41.1 μJ, 100.1 μJ, 243.8 μJ, 593.9 μJ and 1446.7 μJ for 14, 32, 64, 128, 256, 512 and 1024 sizes, respectively.

For the digital system (Nvidia GTX 285), as shown in **Fig. S7b** of the main text, the execution time of the Neural ODE and LSTM with 400 solution steps significantly exceeds that of GRU and RNN. For instance, at a hidden layer size of 1024, the execution times are 4779.5 μs, 4789.2 μs, 3594.8 μs and 1201.3 μs, respectively. When the hidden layer size reaches 1024, our analog system (23.6 μs) achieves a 202.5× improvement in speed compared to the Neural ODE on the digital system (4779.5 μs).

As shown in **Figure 5i** of the main text, the Neural ODE and LSTM also consistently exhibits the highest energy consumption compared to GRU, and simple RNN in the digital system (GTX 285). For example, at a hidden layer size of 1024, the energy consumption values are 975 mJ, 977 mJ, 733 mJ, and 245.1 mJ, respectively. In contrast, our analog system with 180 nm technology node memristor achieves an energy consumption of 1.4 mJ with 673.9× energy efficiency improvements at the same 1024 hidden layer size.

As shown in **Fig. S13**, we also compared our system with GPUs of other technology nodes, such as 90 nm and 65 nm. Although our analog system was implemented using 180 nm technology node, the execution time and energy consumption are smaller than GPUs using more advanced technology node at 1024 hidden size, thanks to the continuous-time and analog in-memory computing.

4. The energy advantage of analog Neural ODE over digital Neural ODE scales up with hidden layer size

The reason that the energy consumption gap between analog and digital systems widens with increasing hidden layer size of the neural network stems from the following: For a smaller (larger) network size, the energy consumption of the analog Neural ODE is more (less) dominated by OPA rather than memristor, leading to a lower (better) energy efficiency and a smaller (larger) gap with the digital counterpart.

4.1 Layer-wise energy consumption: Supplementary Table 2 lists the number of memristors, OPAs and associate energy consumption in a four-layer multilayer (MLP) Neural ODE, where d_{in}, d_{out}, and N are the input, output and hidden dimensions. Here, $E_{memristor}$ represents the average energy consumption of a memristor in handling a given temporal input, while E_{MAC} represents the MAC energy of a single weight in handling a given temporal input on digital hardware, which varies depending on the specific GPU architecture employed. Notably, the memristor energy consumption scales quadratically ($\propto N^2$) while the OPA population increases linearly ($\propto N$) within the hidden layer.

Based on the **Supplementary Table 2**, we compute the total energy consumption of analog Neural ODE and digital Neural ODE in handling a single temporal input, as follows:

$$E_{Analog} = E_{OPA} \times \left((1 + depth) \times N + d_{out}\right) + 4E_{memristor} \times (d_{in} \times N + depth \times N^2 + N \times d_{out}) \qquad (5)$$

$$E_{Digital} = E_{MAC} \times 2 \times (d_{in} \times N + depth \times N^2 + N \times d_{out}) \qquad (S6)$$

4.2 Ratio between analog and digital neural ODE energy: Eq. (S7) calculates the energy consumption ratio between neural ODE on analog solver and digital hardware:

$$\frac{E_{Analog}}{E_{Digital}} = \frac{E_{OPA} \times ((1+depth) \times N + d_{out})}{E_{MAC} \times (d_{in} \times N + depth \times N^2 + N \times d_{out})} + \frac{2 \times E_{memristor}}{E_{MAC}} \qquad (S7)$$

Eq. (S7) comprises two terms. In the first term, the numerator grows linearly with N (through the factor $((1 + depth) \times N)$, while the denominator contains both linear and quadratic terms in N, notably the quadratic term $(depth \times N^2)$. As a result, for large *N*, the quadratic growth in the denominator dominates, causing this term to decrease approximately as $1/N$. The second term is independent of N. Thus, as shown in **Fig. S12**, $E_{Analog}/E_{Digital}$ asymptotically converges to the constant $2E_{memristor}/E_{MAC}$ as N increases. This implies that for larger

(smaller) N, the overall energy efficiency is determined primarily by the energy consumption of memristor (OPA). As $E_{memristor} \ll E_{MAC} \ll E_{OPA}$, the energy performance gap between analog memristor-based Neural ODE and digital Neural ODE widens with increasing N.

Supplementary Note 3 | Illustration of two inference modes in time series prediction.

The following analysis is structured into three parts: ***Part 1*** introduces two distinct training/inference modes—teacher forcing and free running. ***Part 2*** presents simulations with these inference modes using Neural ODE and RNN models. ***Part 3*** offers a detailed error analysis.

1. Teacher Forcing vs. Free Running: In training and inference with temporal models, two common strategies for handling sequential data are **Free Running (FR)** and **Teacher Forcing (TF)**, as shown in **Fig. S14**.

1.1 Free Running allows the model to use its own predictions as inputs for next time steps, rather than relying on any external input over time. This approach evaluates the model's capacity for autonomous sequence generation.

In Neural ODE, free running mode enables the simulation of continuous dynamics by solving the ordinary differential equation: $\frac{dy(t)}{dt} = f(y(t), t, \theta)$. As shown in **Fig. S14a**, an ODE solver integrates from the initial value g_0 over time, iteratively updating its state $y(t)$, as described by the following expressions:

$$y(t_n) = y(t_0) + \int_{t_0}^{t_n} f(y(t), t, \theta)dt, \ where\ y(t_0) = g_0 \quad \text{(S8)}$$

Notably, Neural ODE performs inference in continuous time, contrasting with the discrete-time inference typical of traditional models.

In free running mode, as shown in **Fig. S14b**, the RNN is a discrete-time model that predict the next state based on the current state and input. During a forward pass, the hidden state and output of the system at different time steps are described by the following:

$$h_{i+1} = RNNCell(h_i, \Delta_t, y_i) \quad \text{(S9)}$$

$$y_{i+1} = Z(h_{i+1}) \quad \text{(S10)}$$

Here, $RNNCell$ denotes the RNN's state evolution function, which integrates both the recurrent hidden state h_i and input y_i, updating the next hidden state h_{i+1} with a time step $\Delta_t = t_{i+1} - t_i$. Starting from an initial hidden state h_0, typically initialized to zeros, the hidden state h_i is recursively updated at each time step t_i. The output at each time step y_{i+1}, is then computed as a function of the hidden state h_{i+1}, usually via a simple linear transformation Z.

1.2 In Teacher Forcing, at each time step, the model's input comprises both the hidden state from the previous step and the ground truth output of the last step, rather than relying on the model's own prediction last time step. This approach is particularly effective during training as it mitigates the risk of error propagation-where incorrect predictions could cascade through subsequent time steps-thereby accelerating the learning process and facilitating faster convergence.

However, Neural ODE is a continuous network. In contrast, teacher forcing is a classical training method for discrete networks, such as RNNs, and is not well-suited for Neural ODE. Therefore, to ensure a fair comparison under teacher forcing, we applied time slicing to the sequences in Neural ODE, as shown in **Fig. S14c**. For discrete-time sequence prediction similar to an RNN over n points, we obtain n-1 time intervals with n-1 ground truth-calibrated initial values for neural ODE. In this way, we perform n-1 integrations to generate a prediction consisting of n-1 points.

In teacher forcing mode, as shown in **Fig. S14d**, an RNN uses ground truth inputs as supervision at each time step [68, 69]. The process begins with a hidden state h_0 and input g_0, which together generate the output y_1. Then, the ground truth output at $t = 1$, g_1, is fed into the model along with hidden state h_1 to produce the next output y_2. This iterates for each subsequent time step.

2. Comparison of teacher forcing and free running on Neural ODE and RNN: We trained both the RNN and Neural ODE using the same dataset, ensuring a fair comparison by maintaining consistent model architectures and parameters (6×256, 256×256, 256×6). Both models were tasked with predicting the same unknown segment of the Lorenz96 system over an identical time horizon. To evaluate their performance, we measured the instantaneous MAE and cumulative MAE over time, providing insights into how each model's prediction accuracy evolves as the forecast progresses.

2.1 In Free Running mode, both Neural ODE and RNN models experience a decline in prediction accuracy over time. As shown in **Fig. S15b**, the performance of the RNN deteriorates significantly, with predictions completely diverging from the ground truth by the 16-second mark. This divergence renders the RNN incapable of producing meaningful forecasts beyond this point.

In contrast, as shown in **Fig. S15a,** Neural ODE demonstrates superior temporal stability, maintaining reasonably accurate predictions beyond 16-second mark. This attributed to its continuous-time framework, which provides a more robust mechanism for modeling the underlying dynamics of the system. The observed degradation in the RNN's performance can be attributed to its inherent discrete-time dynamics. This issue is exacerbated in free running mode, where predictions are fed back into the model as inputs.

2.2 In Teacher Forcing mode, both Neural ODE and RNN exhibit highly accurate predictions without clear error accumulation, as shown in **Fig. S16a** and **Fig. S16b**. The predicted waveforms closely match with the ground truth, indicating that both models effectively capture the Lorenz96 system's dynamics when true waveforms are provided at each time step to predict that of the next time step.

3. Quantitative evaluation of prediction errors

We benchmark the performance of RNN and neural ODE in free running and teacher forcing modes using both instantaneous MAE analysis and cumulative MAE analysis.

3.1 Instantaneous MAE analysis: To assess the temporal prediction accuracy of Neural ODE and RNN in both teacher forcing and free running modes, we evaluated the Instantaneous MAE over time. The Instantaneous MAE, averaged across the *d* dimensions, is defined as follows:

$$MAE(t) = \frac{1}{d}\sum_{i=1}^{d}\left|y_t^i - \hat{y}_t^i\right| \quad (S11)$$

where y_t^i denotes the model's output at the time step *t* for dimension *i*, and $\hat{y}_t^i$ represents the corresponding ground truth. As shown in **Fig. S17** in free running mode, the error increases over time for both models, with the RNN exhibiting faster error growth compared to Neural ODE. This is due to the RNN's nature as a discrete-time model with truncation error.

Conversely, in teacher forcing mode, both models maintain very low MAE throughout the prediction period, with Neural ODE demonstrating slightly better accuracy than the RNN. This improved performance can be attributed to Neural ODE's continuous-time framework, which reduces the truncation error inherently present in RNNs.

3.2 Cumulative MAE analysis: We also computed the cumulative MAE by integrating the instantaneous MAE over time. While the instantaneous MAE captures the error at each time step, the cumulative MAE reveals how errors cumulate over time, which is as follows:

$$Cumulative\ MAE(t) = \sum_{j=1}^{t} MAE(j) = \sum_{j=1}^{t} \frac{1}{d} \sum_{i=1}^{d} \left| y_t^i - \hat{y}_t^i \right| \tag{S12}$$

As illustrated in **Fig. S18**, the cumulative MAE(t) for the RNN in free-running mode **(RNN-FR)** increases significantly more rapidly than for **Neural ODE-FR**. Notably, after the 16-second mark, a pronounced divergence occurs, with the gap between the two models widening considerably. This difference can be attributed to the discrete-time nature of RNNs, which can easily lead to truncation error, compared with Neural ODE with continuous-time dynamics. In teacher forcing mode, **Neural ODE-TF** consistently maintains a lower cumulative error than **RNN-TF**, where both models exhibit low cumulative MAEs**.**

3.3 Clarification on Instantaneous and Cumulative MAE

Temporal models that predicting indefinitely into the future is infeasible; the reliability of predictions diminishes as the prediction horizon extends. So instantaneous MAE of both RNN and neural ODE always increases in free running mode but stay relatively constant in teacher forcing mode. In addition, cumulative MAE always increases as it's the temporal integration of instantaneous MAE.

3.3.1 Consistent increase of instantaneous MAE in free running mode, relatively stable instantaneous MAE in teacher forcing mode: In free running mode, instantaneous MAE increases in both Neural ODE and RNN. This rise is attributed to the accumulation of errors, as the model's predictions progressively diverge from the ground truth in the absence of supervision. However, Neural ODE features a slower MAE growing than RNN due to its continuous-time framework, as shown in **Fig. S24**. In contrast, in teacher forcing mode, both networks maintain relatively stable instantaneous MAE, benefiting from corrective feedback at each time step [70, 71].

3.3.2 Consistent increase of cumulative MAE in both teacher forcing and free running modes: The cumulative MAE sums the instantaneous MAE over time as shown in Eq. (S12). Since instantaneous MAE is calculated by taking the absolute value of the error at each time

step, it is always non-negative. As a result, the cumulative MAE necessarily increases over time, as it represents the cumulation of these non-negative errors, as shown in **Fig. S24d.**

Supplementary Note 4 | Scalability of analog ODE with complex dynamic datasets.

We have now applied our approach to the more complex *Electricity* dataset (***part 1*** and ***2***), with 30, 50 and 70 state variables. Additionally, we have included a comparison between our method and traditional simple RNNs (***part 3***), which shows that our approach achieves significantly lower errors when addressing higher-dimensional problems.

1. *Electricity* dataset: We use *Electricity* dataset, which is available at UCI Machine Learning Repository, containing records of residential electricity consumption [72, 73]. Due to the presence of missing or zero values in certain dimensions for the year 2011, we excluded the records from that year. The dataset now encompasses electricity consumption data for 321 clients spanning the years 2012 to 2014. Additionally, we aggregated the data from 15-minute intervals to hourly consumption. Several dimensions (i.e. clients) were randomly selected for visualization, as shown in **Fig. S19**.

2. Data Processing

2.1 Dynamic dimensionality: The dataset consists of 321 users. To create an N-dimensional dataset for training (where N=30, 50 and 70), we randomly select N users from the dataset.

2.2 Segment selection: Over a timespan of more than two years, we extracted a 300-hour subset of the data for training. To prepare the dataset for continuous-time modeling, we applied the interp1d function from the SciPy library, along with a smoothing technique, to interpolate the data.

2.3 Min-max scaling: We applied min-max scaling to normalize the data to a fixed range, specifically [-5, 5], ensuring that all features were brought to a comparable scale.

3. Modeling and prediction of *Electricity* dataset

3.1 Experiment set up: The dataset is represented as a multi-dimensional trajectory consisting of 3,000 time points, with dimensions N = 30, 50, 70. For model training, we split the dataset into two subsets: the first 2,700 time points (90% of the total) were used for training, while the remaining 300 time points (10%) were reserved for validation.

3.2 Model specification: To ensure a fair comparison, we included a traditional simple RNN as a baseline. As shown in **Supplementary Table 3**, we aligned the model specifications of the Neural ODE and the RNN for fair evaluation of their performance under comparable model sizes.

3.2.1 Training process: During training, both Neural ODE and RNN employ the teacher forcing mode; however, there is a slight difference in how it is applied, as shown in **Fig. S20**. For Neural ODE, we adopt a segment-to-segment approach with teacher forcing [74]. During training, we randomly sample 20-time-point segments from a total of 1800 time points of the dataset. Each batch consisted of 64 such segments, and the model was trained over 2,000 epochs. For clarity, we illustrate the process using a simplified three-point segment, as shown in **Fig. S20a**. We consider three points, g_0, g_1 and g_2, as a short segment from the training dataset. Here g_0 and $t = (t_0, t_1, t_2)$ are the model input, while $g_1$and g_2 are the ground truth. During a forward pass, the system evolves according to the following equation:

$$y_1, y_2 = ODESolver\big(y_0, f_\theta, (t_0, t_1, t_2)\big) \tag{S13}$$

where $y_0 = g_0$ represents the initial state of the system at time t_0, and f_θ is the trainable neural network. The time points t_1 *and* t_2 correspond to the moments at which the network's state is computed. In this way, the states y_1 and y_2 are predicated, and the loss is computed by comparing these predictions to the ground truth g_1 and g_2.

As shown in **Fig. S20b**, the RNN is trained using the point-to-point teacher forcing method. Different from neural ODE, the RNN is a discrete-time model that predicts the next output based on the current state and input. During a forward pass, the system updates the hidden state according to Eq. (S14) and produces its output according to Eq. (S15):

$$h_{i+1} = RNNCell(h_i, \Delta_t, y_i) \tag{S14}$$

$$y_{i+1} = Z(h_{i+1}) \tag{S15}$$

Here, $RNNCell$ denotes the RNN's state evolution function, which receives both the recurrent hidden state h_i and input y_i as well as updates the next hidden state h_{i+1} with a time step $\Delta_t = t_{i+1} - t_i$. Starting from an initial hidden state h_0, typically initialized to zero, the hidden state h_i is recursively updated at each time step t_i. The output at each time step y_{i+1}, is then computed as a function of the hidden state h_{i+1}, usually via a simple linear transformation *Z*.

It’s worthy to note that the number of ground truth labels used in the Neural ODE is significantly smaller than that in the RNN. In the Neural ODE, each segment comprises 20 time points, but only one ground truth label (the initial state) is assigned per segment. In contrast, the RNN receives a ground truth label for each individual time point. As a result, the error in the Neural ODE is calculated once for every 20 time points, whereas in the RNN, the error is computed at every time point, providing more frequent supervision. Despite the reduced supervision in the Neural ODE, it exhibits superior performance, as will be discussed in the following section.

3.2.2 Validation and inference process: During the validation and inference phases, both models operate in free-running mode, as used in real-world applications. This approach eliminates the use of ground truth labels during prediction, where the models generate sequential outputs based solely on their initial conditions (see Supplementary Note 3 for details on the two modes employed in time series modeling).

3.3 Comparison of interpolation abilities between our approach and RNN during training: As shown in **Fig. S21**, shows the instantaneous MAE between the training results and the ground truth over 270-hour period for both Neural ODE and RNN, applied to *Electricity* dataset with 30, 50 and 70 dimensions. Each dimension corresponds to the electricity consumption of a distinct client. Contour plots visualize instantaneous MAE, where purple regions are of lower error, indicating high accuracy, while cyan and orange regions correspond to higher error levels. In **Fig. S21a-c**, our approach demonstrates better interpolation performance, closely aligning with the ground truth over time and maintaining a larger region of purple, indicative of lower error. In contrast, **Fig. S21d-f** shows that the RNN exhibits a suboptimal interpolation performance, with a higher prevalence of cyan regions. This diminished accuracy is primarily attributed to the limitations of the discrete-time model and truncation errors, which result in reduced precision during the training process.

3.4 Comparison of extrapolation abilities between our approach and RNN during inference

3.4.1 Simulation results: To evaluate the long-term predictive capabilities and scalability of our approach, we extended the predictive time range to 90 hours across 30, 50 and 70 dimensions, as shown in **Fig. S22**.

In **Fig. S22a-c,** the purple regions show low deviations between Neural ODE's predictions and the ground truth for the *Electricity* dataset, indicating minimal discrepancies at shorter predictive times, particularly during the first 60 hours. In contrast, **Fig. S22d-f** shows a significant increase in the instantaneous MAE for the RNN over time, which can be attributed to the limitations of discrete-time models and their sensitivity to truncation errors.

Both the Neural ODE and RNN exhibit a rising trend in instantaneous MAE as dimensionality increases. However, the Neural ODE consistently outperforms the RNN, especially in maintaining a slower rate of MAE escalation over extended time periods and across higher dimensions. This underscores the superior long-term predictive accuracy and scalability of the Neural ODE compared to the RNN.

3.4.2 Result analysis: In the free-running mode, the Neural ODE outperforms the RNN, exhibiting a lower instantaneous and cumulative MAE.

As shown in **Fig. S23a-b,** the instantaneous MAE of both the Neural ODE and RNN across 30, 50, and 70 dimensions follows a similar upward trend over time. However, compared to the RNN, the Neural ODE maintains a consistently lower instantaneous MAE while avoiding significant fluctuations, indicating its superior scalability and robustness when applied to datasets with higher dimensions.

In **Fig. S23c-d**, shows the cumulative MAE over a 90-hour period for both the Neural ODE and RNN, with predictions made across 30, 50, and 70 dimensions. Compared to the RNN, Neural ODE features a lower cumulative MAE and a slower rate of increase in cumulative MAE.

Supplementary Note 5 | Impact of Lyapunov times.

In ***Part 1***, we provide a comprehensive introduction to the concept of Lyapunov time. In ***Part 2***, we detail the methodology used to compute Lyapunov times in the context of the Lorenz96 system. Finally, in ***Part 3***, we use Lyapunov times to benchmark our Neural ODE in modeling the Lorenz96 dynamics.

1. Definition of Lyapunov times

In chaotic systems, the point at which trajectories with slightly different initial conditions begin to significantly diverge is typically defined by the **Lyapunov time** [75, 76]. This metric is crucial for measuring the predictability of chaotic dynamics models, as it defines the time horizon over which a model's predictions are likely to remain accurate.

- **Within the Lyapunov time**: As shown in **Fig. S25**, for timescales shorter than the Lyapunov time, the model can produce reasonably accurate predictions. During this period, the divergence between trajectories due to errors in the initial conditions is minimal, allowing the model's forecasts to remain reliable. Small uncertainties in the input do not significantly impact the outcome, maintaining predictive accuracy.
- **Beyond the Lyapunov time**: For predictions extending beyond the Lyapunov time, the system's sensitivity to initial conditions becomes pronounced. Even minor discrepancies in the initial state grow exponentially, leading to substantial divergence between predicted and actual outcomes. At this point, the model's predictive power rapidly deteriorates, rendering forecasts unreliable.

2. Lyapunov time calculation for the Lorenz96 dynamics

2.1 Setting up the Lorenz96 dynamics: The Lorenz96 dynamics with dimension $n \in \mathbb{N}$ is governed by the equation for the i-th variable, given by:

$$\frac{d\boldsymbol{X}_i}{dt} = (\boldsymbol{X}_{i+1} - \boldsymbol{X}_{i-2})\boldsymbol{X}_{i-1} - \boldsymbol{X}_i + \boldsymbol{F} \tag{S16}$$

where $\mathbf{X}_i$ is an N-point time series, $\{x_{i,1}, x_{i,2}, \dots, x_{i,N}\}$. The indices are taken modulo n by applying the following boundary conditions: $\boldsymbol{X}_{i-\mathrm{n}} = \boldsymbol{X}_{i+\mathrm{n}} = \boldsymbol{X}_i$, resulting in a model with circulant symmetry. Note that both the dimension $n \in \mathbb{N}$ and the forcing parameter $F \in \mathbb{R}$ are free parameters.

2.2 Initial conditions and perturbation: To begin, select an initial state $\boldsymbol{X}_{n,0} = [-1.2061, 0.0617, 1.1632, -1.5008, -1.5944, -0.0187]^{\mathrm{T}}$. To generate a second, nearby trajectory, we introduce a small perturbation $\widehat{\boldsymbol{X}}_{n,0} = \boldsymbol{X}_{n,0} + \boldsymbol{\delta}(\boldsymbol{t_0})$, where $\boldsymbol{\delta}(\boldsymbol{t_0}) = [\varepsilon, 0, 0, 0, 0, 0]^T$ and $\varepsilon=10^{-8}$.

2.3 Numerical integration of trajectories: Both the unperturbed and perturbed systems are integrated over time using a suitable numerical method, such as the fourth-order Runge-Kutta method. Initially, the separation between the trajectories equals $\boldsymbol{\delta_0} = \boldsymbol{\delta}(\boldsymbol{t_0})$. The integration is carried out over a sufficiently long period to capture the chaotic behavior of the system. As the system evolves, the separation $\delta(t)$ between the two trajectories increases exponentially, illustrating the sensitivity to initial conditions — a hallmark of chaotic systems.

2.4 Measuring divergence between trajectories: When the Lorenz96 dynamics is chaotic, the trajectories diverge, on average, at an exponential rate characterized by the largest Lyapunov exponent. To calculate the largest Lyapunov exponent, we monitor the evolution of the separation between the two trajectories over time [77, 78]. Let the initial separation be $\boldsymbol{\delta_0}$. At any subsequent time t, the separation between the trajectories, $\delta(t)$, is measured. In a chaotic system, this separation grows exponentially and can be approximated by [79]:

$$\delta(t) \approx \delta_0 e^{\lambda_{max} t} \tag{S17}$$

where λ_{max} is the largest Lyapunov exponent. This exponential divergence continues until the perturbation grows large enough for nonlinear effects to dominate.

2.5 Logarithmic growth and linear fitting: To estimate the largest Lyapunov exponent λ_{max}, we analyze the logarithmic growth of the separation:

$$ln(\frac{\delta(t)}{\delta_0}) \approx \boldsymbol{\lambda_{max} t} \tag{S18}$$

By plotting $\mathbf{ln}(\frac{\delta(t)}{\delta_0})$ as a function of time $\boldsymbol{t}$, a linear relationship is obtained. The slope of the best-fit line corresponds to λ_{max}.

2.6 Calculation of Lyapunov Time: Once λ_{max} is determined, the Lyapunov time T_L, which represents the timescale over which predictability is lost, is simply the inverse of the largest Lyapunov exponent λ_{max}:

$$\boldsymbol{T_L = \frac{1}{\lambda_{max}}} \tag{S19}$$

For example, in the case of the Lorenz96 system with parameters *F=6* and *N=6*, the largest Lyapunov exponent is found to be approximately $\lambda_{max} \approx 1.68$. Hence, the Lyapunov time is approximately $T_L \approx 0.595$ s (with units consistent with the system's time units), indicating that initial uncertainties in the system's state will grow significantly beyond this period.

3. Impact of Lyapunov time on the prediction of Lorenz96

3.1 Lyapunov time at different forcing term (*F*): In the Lorenz96 system, the forcing term *F* plays a crucial role in determining the chaotic system's behavior. As shown in **Fig. S26**, increasing *F* causes the system to transition from stable to chaotic dynamics. At lower values of *F*, the system primarily exhibits periodic or quasi-periodic solutions. However, as *F* increases, the system becomes more sensitive to initial conditions, leading to the onset of chaotic dynamics. This transition is marked by the appearance of a positive Lyapunov exponent, signifying a reduction in predictability. The Lyapunov time, which decreases as *F* increases, provides a measure of how quickly predictability is lost in the system

3.3 Extrapolation performance at $\boldsymbol{F = 6}$: As shown in **Fig. S27**, it shows that the extrapolation performance of Neural ODE over time compared with the ground truth. Top panel shows the ground truth of the Lorenz96 system with dimension *N=6*, where the middle panel presents the Neural ODE's extrapolation results over 20-second period. We calculated the MAE between the prediction result and the ground truth, as shown in bottom panel. At the forcing term *F=6*, its corresponding Lyapunov time T_L is 8.60 seconds. Notably, our model is capable of accurately predicting the Lorenz96 dynamics up to 14.21 seconds (see section 3.4

for the definition of the prediction horizon). This result indicates that the model achieves a prediction horizon approximately 1.65 times of the Lyapunov time, highlighting its robust extrapolation ability even in the face of chaotic dynamics.

3.4 Lyapunov time under different forcing terms *F*: We conducted a series of simulations to assess the predictive abilities of Neural ODE across increasing forcing term F, which directly correlates with the chaotic intensity of the Lorenz96 dynamics. As previously discussed, we calculated the Lyapunov time T_L for the different value of F. We then assessed the prediction horizon of Neural ODE and compared it to the corresponding Lyapunov time T_L.

Prediction Horizon is defined as the temporal limit within which a model's forecasts remain accurate before the error exceeds a specified threshold. Here, we define the **Prediction Horizon** of Neural ODE as the earliest time point at which the instantaneous MAE exceeds the predefined threshold (ϵ), which is derived from the 95% confidence intervals of the prediction errors (see section 3.5 and 3.6 for the information of the confidence interval).

3.5 Confidence intervals for time series prediction: CIs provide a statistical framework for quantifying uncertainty in time series forecasting [80, 81]. In our study, CIs function as predefined thresholds (ϵ) to define the prediction horizon (t_{PH}). When prediction results fall within this prediction horizon, the model accurately captures the underlying dynamics of the data. Conversely, exceeding this prediction horizon may indicate model inadequacies or unaccounted-for stochastic fluctuations. Thus, CIs serve as an index for distinguishing genuine systematic trends from random noise.

3.6 Estimation of the 95% confidence interval and prediction horizon: The 95% CI is a widely used benchmark in time-series prediction, balancing between statistical stringency and interpretability [82]. The associated prediction horizon (t_{PH}) is defined as the period during which model prediction results remain within the 95% CIs, beyond which predictions are statistically unreliable.

Formally, we define the prediction error at each time step by:

$$e(t) = y_{true}(t) - y_{pred}(t), t = 1, \dots, N \quad \text{(S20)}$$

Assuming $e(t)$ follows a normal distribution, the upper and lower bounds of the 95% CI can be estimated using the sample mean $\bar{e}$ and sample standard deviation s, as follows:

$$\epsilon_{upper} = \bar{e} + z_{0.95} \times s \quad \text{(S21)}$$

$$\epsilon_{lower} = \bar{e} - z_{0.95} \times s \quad \text{(S22)}$$

Under the assumption of a symmetric error distribution centered around zero, the sample mean is typically approximated as zero $\bar{e} \approx 0$, simplifying the CIs to $\epsilon = z_{0.95} \times s$, where $z_{0.95} \approx 1.96$. The prediction horizon t_{PH} is the earliest time point at which the model's prediction error surpasses this threshold ϵ in all dimensions [81], as follows:

$$t_{PH} = min\{t \mid |e_i(t)| > \epsilon, \forall i \in \{1,2,\ldots,d\}\} \quad \text{(S23)}$$

Thus, t_{PH} marks the threshold beyond which forecasts lack statistical reliability under the 95% confidence criterion. As shown in **Supplementary Table 4**, we summarize the Lyapunov time T_L and corresponding prediction horizon t_{PH} of Neural ODE across different forcing parameter F in the Lorenz96 dynamics. Additionally, **Supplementary Table 4** also includes the ratio between the prediction horizon t_{PH} and the Lyapunov time T_L. This ratio provides a quantitative measure of how long the Neural ODE's predictions remain reliable in relation to the Lorenz96 system's inherent chaotic timescale.

Supplementary Note 6 | Training performance for our digital twin.

This note offers a comprehensive analysis of the training (interpolation) and predictive (extrapolation) performance of our digital twin, which is based on the Lorenz96 dynamics. Our objective is to provide a thorough understanding of the effectiveness and efficiency of our methodology.

1. Extrapolation performance analysis. The network's predictive capability is rigorously evaluated using time series data, including tasks with unseen data that were not part of the training process. **Fig. S24a** showcases a contour plot that provides a visual representation of the error landscape for our network's predictions over a 20-second period. This plot enables us to identify patterns and regions where prediction errors are concentrated, shedding light on the temporal dynamics of the network's performance.

To further assess the network's predictive accuracy, **Fig. S24b** presents a side-by-side comparison of the predictive output (depicted by the red line) and the ground truth (represented by the black line). Discrepancies between these lines indicate prediction errors, while close alignment demonstrates the network's ability to accurately track the ground truth over time. **Fig. S24c-d** present cumulative error curves for all six channels, illustrating the total accumulated error over time. Although the network's cumulative error increases over time, indicating a decrease in predictive accuracy, the error remains relatively low. Notably, our network shows satisfactory precision in predictions up to a 10-second timespan.

2. Performance analysis of training process. This section examines the impact of various hyperparameters and training conditions on the performance of our digital twin model. Through an extensive search, we aimed to identify optimal network configurations by considering factors such as activation functions, the number of sampling points, loss functions, ODE solver methods, and optimization algorithms. **Fig. S28** illustrates the effects of these factors on the training process as a function of iterations, including:

- **a** compare the training loss across epochs for different activation functions. We observed that the ReLU activation function enables faster convergence of the training loss, indicating its efficiency for our network architecture.
- **b** illustrates the impact of varying the number of sampling points for a 40-second task duration. The training process appears to be unstable at the extremes, with either too

few (500 points) or too many (4000 points) sampling points. Based on these findings, we opted for an intermediate value of 2000 sampling points in our experiments to balance stability and performance.

- **c** compares different loss functions, including Mean Squared Error (MSE), Mean Absolute Error (MAE), and Dynamical Time Wrapping (DTW). The DTW function demonstrated more stable convergence and yielded lower MAE, serving as a standard for uniform evaluation across conditions. DTW's ability to capture temporal relationships makes it particularly suitable for training time series models.
- **d** showcases the performance of different ODE solver methods (Dopri5, RK4, Euler). Both RK4 and Dopri5 exhibited more stable convergence. However, with sufficient epochs, the final error did not significantly differ between the solver methods, indicating that given enough time, each method can achieve comparable accuracy.
- **e** highlights the impact of different optimization algorithms on the training process. The Adam optimizer is found to facilitate quick convergence of the loss function, indicating its effectiveness for our network's learning dynamics.

3. Performance analysis of interpolation and extrapolation error. According to the simulations, we repeated each training condition ten times to capture the variability in interpolation (left side) and extrapolation errors (right side), as shown in **Fig. S29**. These boxplots encapsulate the range of outcomes from the repeated experiments, highlighting the median, interquartile range (IQR), and any potential outliers in the error distributions.

4. Performance analysis of different hidden layer size. Additionally, we conducted a comparative analysis of our proposed model with three established time-series forecasting models: Long Short-Term Memory (LSTM), Gated Recurrent Unit (GRU), and Recurrent Neural Network (RNN). This comparison evaluates performance across diverse network architectures at multiple scales. We assessed these models using six distinct network configurations, determined by varying the number of intermediate layers (either one or two) and the number of neurons per layer (64, 128, or 256). This approach facilitated a thorough evaluation across a spectrum of model complexities. Each model was tested in both interpolation and extrapolation tasks to ascertain its effectiveness in managing time-series forecasting both within and outside the scope of the training dataset. **Fig. S30** demonstrates

that our model not only matches the performance of traditional forecasting models but also shows enhanced stability in both interpolation (red) and extrapolation tasks (blue). This increased stability is particularly notable in scenarios involving larger network scales, where conventional models often face challenges in maintaining performance due to the growth in parameters and the risk of overfitting.

Supplementary Note 7 | Accuracy comparison between digital and analog ODE solver.

In this note, we have conducted additional experiments to compare the inference accuracy of standard digital Neural ODE solvers and our analog solver, specifically in the presence of programming noise and read noise.

In ***part 1*** of the following response, we introduce the four digital ODE solvers evaluated alongside our analog solver. In ***part 2***, we outline the simulation setup used for solving the ODEs, ensuring that all solvers are compared under consistent and fair conditions. In ***part 3***, we compare the accuracy of both digital (of different solution steps) and our analog solvers. Notably, our analog solver exhibited robustness to read noise and demonstrated a degree of resilience to programming noise within a certain range.

1. Introduction: In this section, we provide an overview of standard digital Neural ODE solvers, as summarized in **Supplementary Table 5**, and compare them with our analog memristive Neural ODE solver.

1.1 Digital ODE solvers

1.1.1 Euler method is a simple and widely used numerical solver that estimates the next point by using the current slope. Although it has low computational complexity, its first-order accuracy often leads to significant error accumulation, especially in stiff or complex systems.

1.1.2 Midpoint method improves on the Euler approach by calculating the slope at the midpoint of the integration interval, resulting in second-order accuracy. This enhancement provides better precision, but at the cost of slightly increased computational complexity due to the additional slope calculation.

1.1.3 RK4 (Fourth-order Runge-Kutta) method is a commonly used fixed-step solver that computes a weighted average from four slope evaluations per step. It achieves fourth-order accuracy, offering a good balance between computational efficiency and precision, making it suitable for a wide range of problems.

1.1.4 Dopri5 (Dormand-Prince 5) method is an adaptive solver that dynamically adjusts the step size based on error estimation. It provides fifth-order accuracy and is highly efficient for

complex systems. However, its adaptive nature requires greater computational resources, especially in systems where frequent step size adjustments are needed.

1.2 Analog memristive Neural ODE solver achieves effectively continuous-time (infinite-step) computation in analog domain, leveraging a combination of in-memory computing and a closed-loop integrator. This analog solver provides high energy efficiency and rapid computation speed. However, the inherent limitations of memristor, such as programming noise and variability, present challenges in achieving high precision.

2. Simulation set up

2.1 Training process: The dataset comprising 2000-time steps is generated from the Lorenz96 system with the dimension $N = 6$ and the forcing term $F = 6$, with the initial condition $[-1.2061, 0.0617, 1.1632, -1.5008, -1.5944, -0.0187]$. The first 90% of the data is used for training, while the remaining 10% is reserved for testing. The Neural ODE consists of a three-layer neural network with dimensions 6×512, 512×512, 512×6, where each hidden layer contains 512 neurons. We adopted a segment-to-segment teacher forcing training (see Supplementary Note 3 for details on the two modes employed in time series modeling). During training, 20-time-point segments were randomly sampled from a total of 2000 time points in the dataset. Dynamic Time Warping (DTW) was employed as the loss function to quantify dissimilarity between the Neural ODE's output and the ground truth. The Neural ODE was trained using the Adam optimizer, which adaptively adjusts the learning rate. Model weights were updated based on the gradients of the DTW loss.

2.2 Inference process: After the training process, the parameters of the Neural ODE are frozen. To ensure a fair evaluation across different solvers, inference is conducted in free-running mode using the same trained Neural ODE parameters, but with different solvers. This enables a direct comparison of the performance between four digital solvers (such as Euler, Midpoint, RK4, and Dopri5) and the analog memristive solver under identical conditions.

3 Performance evaluation

3.1 Interpolation performance under varying solution steps: The objective of this analysis was to evaluate the impact of solution step sizes of digital solvers on its accuracy. As shown in **Fig. S31**, we evaluated the interpolation performance of the trained Neural ODE using the RK4

method under varying solution steps. For clearer visualization, we selected the first dimension of the Lorenz96 system (with $D = 6$) for comparison (black lines).

When 200 solution steps were used, the RK4 solver failed to capture the key characteristics of the Lorenz96 dynamics over the 0 to 36-second interval, because numerical truncation errors lead to inaccurate interpolation. As the number of solution steps increased, the performance of the solver improved. For example, with 1200 solution points, the solver achieved accurate interpolation over 15 seconds. Moreover, with 1800 or 2000 solution steps, the solver demonstrated accurate prediction over even longer intervals, such as 36 seconds, in free-running mode.

Trade-off: The number of solution steps determines both the accuracy and stability of digital solvers. While increasing the number of steps enhances accuracy, reducing them introduces significant truncation errors. However, a higher number of steps also incurs greater computational costs, leading to slower solving speeds and increased energy consumption. Therefore, a balance must be achieved between accuracy and computational efficiency.

3.2 Evaluation of accuracy

In the following, we compare the accuracy between digital solvers (of different solution step sizes) and our analog ODE solver.

3.2.1 Digital Neural ODE solver: In this case, we utilize the instantaneous MAE to evaluate the discrepancy between the output of the digital ODE solvers and the ground truth. As shown in **Fig. S32a**, the accuracy of all four digital solver improves as the number of solution steps increases. Among these, the RK4 method achieves the lowest error at 2000 solution steps, followed by the Midpoint and Dopri5 methods, with the Euler method exhibiting the highest error. Specifically, the MAE for inference using the four digital solvers (Euler, Dopri5, Midpoint, and RK4) is 3.59, 0.93, 0.77, and 0.28, respectively.

3.2.2 Analog Neural ODE solver: As shown in **Fig. S32a**, we present the MAE of our analog ODE solver under various noise conditions. The noise-free analog solver is of an MAE 0.410, which is slightly higher than the 0.278 MAE of the high-precision but power-intensive RK4 method with 2000 solution steps while lower than the Midpoint (MAE = 0.766), Dopri5 (MAE=0.932), and Euler methods (MAE = 3.588). In addition, our analog ODE solver is robust to read noise of memristors. With 1% read noise injection, its MAE increases only slightly to 0.417, and it still outperforms all digital solvers except for RK4. Even with the injection of programming noise and read noise—such as (2% programming noise, 0% read noise) or (2%

programming noise, 2% read noise)—our analog solver continues to achieve an acceptable MAE relative to the Midpoint and Dopri5 methods.

As shown in **Fig. S32b**, our analog solver demonstrates notable robustness against read noise. When programming noise is fixed, increases in read noise causes only slight fluctuations in interpolation MAE (see **Supplementary Note 3** for details on robustness). Similarly, programming noise within a certain range does not significantly affect interpolation MAE. For instance, the memristor of our analog ODE solver can maintain programming noise within 2% using a write-and-verify programming scheme (see **Fig. S3** for details on the programming scheme of the analog memristor array), ensuring the solver maintains acceptable performance even in the presence of both noises.

Supplementary Note 8 | Limitations of the analog ODE solver with potential solutions.

This note systematically examines the limitations of analog ODE solvers and potential solutions. The discussion is organized into two parts. Specifically, we discuss the fidelity limitations of digital twin due to analog neural ODE hardware noise (***part 1***) and size limit of neural network (***part 2***).

1. Analog neural ODE solver hardware noise

As shown in **Fig. S33**, hardware noise arises from the intrinsic stochasticity of memristors and imperfections in operational amplifier (OPA)-based circuits, which introduce perturbations to model weights, nonlinear activations, and integration, thereby compromising computational accuracy.

1.1 Programming and reading stochasticity of memristors

First, fidelity limitations of our analog neural ODE solver arise from programming and reading stochasticity of memristors. Programming stochasticity introduces inevitable errors to memristor conductance, while read stochasticity causes fluctuations in the readout current. Although these two types of stochasticity are minor, they accumulate and are amplified over time, ultimately degrading modeling precision.

To mitigate programming and reading stochasticity, high-precision programming protocols and readout circuit optimization have been proposed, respectively.

1) **High-precision programming protocol**: Various programming protocols have been developed to address the stochastic nature of memristor programming. For example, Prof. J. Joshua Yang's team proposed a protocol where a numerical value is represented as a weighted sum of multiple memristors; errors from earlier programming steps are compensated by subsequently programmed devices. This approach enables low-precision analog memristors to collectively achieve high-precision computations [83].
2) **Readout circuit optimization**: Multiple techniques have been developed to mitigate sensing current fluctuation. Examples include a parallel-series reference cell (PSRC) for current-mode sensing circuits to suppress reference current variation [84], a voltage-swing remapping voltage sense amplifier (VSR-VSA) for voltage-mode sensing circuits [85], and even a current-voltage-hybrid analog-to-digital converter (CVH-ADC) to enhance signal margins [86].

1.2 Imperfections of operational amplifier

Second, the fidelity of our analog ODE solver is constrained by imperfections inherent to OPA-based circuits. Key functional blocks of the analog neural ODE solver—including ReLU, inverting amplifiers, and integrators—are all implemented using OPAs. As a result, these imperfections can collectively compromise both the accuracy and stability of the system [87]. To address this issue, feedback loop optimization and noise/distortion cancellation have been proposed to enhance OPA stability and reliability.

1) **Feedback loop optimization**: Paralleling the feedback resistor with a capacitor introduces a zero in the transfer function, improving phase margin and circuit stability. This configuration also reduces high-frequency impedance, effectively suppressing noise and parasitic oscillations, thereby enhancing signal integrity in precision applications [88].
2) **Noise/distortion cancellation**: By introducing a phase-inverted signal path, noise and harmonic distortion can be suppressed through destructive interference, while preserving the in-phase desired signal. This technique improves the signal-to-noise ratio and enhances amplification fidelity, making it well-suited for high-precision analog computing [87].

2. Size limit of neural network

Neural network size is constrained by memristor array physical size. IR drops across large memristor arrays hinder voltage uniformity, thereby restricting the maximum feasible array size.

2.1 IR drop in memristor arrays

As shown in **Fig. S33**, the neural network size of our analog ODE solver is constrained by IR drop within memristor arrays. As the array size increases, voltage degradation along the WLs and BLs becomes increasingly pronounced due to the intrinsic resistance of metal interconnects. This effect arises from two compounding factors. First, the extension of interconnect length introduces greater voltage drops. Second, a larger number of active memristors increases the total current drawn through the array, further amplifying voltage drops. As a result, memristors farther from the voltage source receive diminished operating voltages introducing computational errors.

To mitigate this issue, optimizations of layout, cell design, driving circuit and multi-bank have proposed.

1) **Layout optimization**: Layout optimization has been reported to minimize IR-drop. For instance, Prof. Ming Liu's team proposed a layout optimization strategy to mitigate IR-drop by balancing voltage variations between near and far cells [89]. By placing the BL

drivers at the top and the SL drivers at the bottom of the array, the IR-drop variation is better balanced. Additionally, TSMC adopted a common SL architecture in memristor arrays to reduce the wire resistance along the read-out SLs. By sharing SLs across multiple columns, the number of tracks is reduced, enabling wider metal tracks and lower resistance [90].

2) **Cell optimization**: Memristive array cell optimization has been reported to mitigate IR-drop. For example, Prof. Huaqiang Wu's team introduced the sign-weighted 2T2R (SW-2T2R) cell design [91], where a differential pair of memristors represents a signed weight. The currents through the memristor differential pairs are locally cancelled out, reducing the cumulative current and thereby alleviating IR-drop.
3) **Driving/sensing circuit optimization**: Driving/sensing circuit optimization has been reported to minimize IR-drop. For example, dividing a large 4MB memristor-based CIM macro into 32 sub-banks (each with a 512 ×1024 memristor sub-array with complete driving/sensing circuit) has been used to minimize IR drop, which implements both MobileNet V3 and ResNet50 [86]. Additionally, Prof. Arijit Raychowdhury's team developed a novel read logic that accounts for expected IR-drop effects [92]. By setting different write configurations that consider cumulative resistance as the WL count increases, read-out errors due to IR-drop were experimentally mitigated.
4) **Software weight mapping optimization**: Software weight mapping optimization has been proposed to reduce IR-drop impact on the network performance. For instance, Prof. Ielmini's team proposed to compensates for IR-drop by duplicating weight matrices and map each replications to a memristor array with a different spatial mapping method [93].

Supplementary Note 9 | Impact of reading and programming noise.

This note examines the effects of reading and programming noise on the analog ODE system, as illustrated in Figure 5j of the main text. The discussion is organized into four parts. In ***part 1***, we introduce the read and programming noises and mechanisms. In ***part 2***, we summarize the literature (Liu et al. [61]) on impact of read noise to Neural ODE. In ***part 3*** we discuss the impact of programming noise to Neural ODE. In ***part 4***, we present the numerical results of read and programming noise influence on our analog ODE solver.

1. Reading and programming noise of memristors

1.1 Reading noise is the temporal fluctuation of the memristor conductance when the memristor is probed by small read voltage (below programming threshold). This read noise arises from conductance fluctuations in nanoscale memristors, which are driven by atomic structure fluctuations (e.g. structural instability of nanoscale conductive filament) and/or electronic trapping/de-trapping (e.g. random telegraphic noise) [94].

1.2 Programming noise is the error in memristor programming, where the actual programmed conductance typically deviates from the target conductance. This deviation varies from device-to-device and cycle-to-cycle. This form of noise is primarily caused by the intrinsic randomness associated with the formation and rupture of conductive filaments due to ionic motion within the memristors [95].

2. Literature on read noise and neural ODE performance

Introducing read noise may not impact on Neural ODE performance. This phenomenon is supported by theoretical analyses in the context of neural stochastic differential equations (Neural SDEs). Liu et al. [61] explored this concept in their paper titled *"How Does Noise Help Robustness? Explanation and Exploration under the Neural SDE Framework"*. According to Liu et al. [61], injecting stochastic read noise during inference can improve generalization and robustness of neural networks [96, 97].

2.1 Theoretical insights

- **Read noise as a regularizer**: Noise injection during inference acts similarly to a regularization technique. It prevents the network from relying too heavily on specific features of the input data, thus reducing overfitting and improving generalization to unseen data [61].

- **Stabilization of learning dynamics**: In the Neural SDE framework, adding noise transforms a deterministic ODE into a stochastic system. This can stabilize the trajectory of hidden states, making the network's output less sensitive to small perturbations in the input [98].
- **Enhanced exploration of the solution space**: Stochasticity allows the network to explore a broader region of the function space during inference, potentially leading to better performance by avoiding poor local minima.
- **Improved robustness**: Read noise can bolster the network's robustness against both adversarial and non-adversarial perturbations, as it makes the decision boundary less sharp and more resilient to input variations [99].

2.2 Numerical results from literature

In Section 4.1 of their study, Liu et al. [61] present numerical evidence demonstrating that the introduction of read noise enhances the robustness of Neural ODEs. Specifically, they compare the performance of standard Neural ODEs with and without the application of read noise during both the training and testing phases.

In their experiments, the function, $f(\cdot)$ is implemented as a neural network composed of convolutional blocks (see [61] for detailed experimental configurations). For the diffusion term $G(\cdot)$, the following definitions are explored:

- **Neural ODE without diffusion**: The diffusion term is omitted by setting $G(h_t, t; v) = 0$.
- **Additive noise**: The diffusion term is independent of h_t and is modeled as $G(h_t, t; v) = \sigma_t I$, where σ_t denotes the noise scale and I is the identity matrix.
- **Multiplicative noise**: The diffusion term is proportional to h_t, and is expressed as $G(h_t, t; v) = \sigma_t h_t$. Read noise of memristor is treated as a specific case of multiplicative noise.
- **Testing time noise (TNN)**: Testing time noise is introduced during inference to evaluate the robustness of the model under noisy conditions.

The experiments are conducted on three benchmark image classification datasets: CIFAR-10, STL-10, and Tiny-ImageNet. The experimental results, summarized in **Supplementary Table 6**, reveal that Neural ODEs incorporating read noise during the testing phase (referred to as TNN) consistently outperform their noiseless counterparts across all datasets. For example, on the CIFAR-10 dataset, the accuracy of Neural ODEs with multiplicative noise but without TNN

is 89.06%, whereas adding TNN increases accuracy to 89.77%, representing a modest improvement. However, the injection of testing time noise can occasionally result in minor accuracy reductions. For instance, on the Tiny-ImageNet dataset, accuracy slightly decreases from 46.94% to 46.65%. Notably, the addition of moderate testing time noise acts as a regularizer, thereby improving test-time accuracy. Furthermore, when testing time noise is retained during inference and the outputs are ensembled, performance is enhanced even further in most cases.

3. Impact of programming noise in Neural ODEs

Programming noise, on the other hand, introduces errors (with a non-zero mean over time) to the weights of Neural ODE. In contrast, read errors are with a zero mean over time. Programming noise has a more significant impact on network performance because:

- **Error accumulation**: In contrast to reading noise, which typically exhibits a zero-mean and can average out over multiple inferences, programming noise induces systematic errors that may accumulate over time.
- **Diminished predictive capability**: Corrupted weights impair the network's capacity to generalize from training data to unseen inference data.

4. Experiments on the influence of read noise and programming noise to analog ODE solver

During inference, various combinations of programming noise and read noise were simulated to assess the robustness of our analog ODE system. As shown in **Fig. S34**, under a constant level of programming noise, the introduction of read noise does not markedly impact performance, even in the free-running mode (refer to **Supplementary Note 3** for details on the two modes employed in time series modeling). For instance, with programming noise fixed at 3%, adding 1%, 2%, and 3% read noise yields mean absolute errors (MAEs) of 0.77, 0.78, and 0.79, respectively, compared to 0.81 in the absence of read noise. This observation is consistent with the literature summary in ***part 2*** regarding the influence of read noise on model performance.

Conversely, as programming noise escalates, the network's inference performance linearly decreases. For example, an increase in programming noise from 0% to 1% results in a minor degradation in performance, yet the impact on model accuracy remains minimal. However,

when programming noise rises to 3%, the MAE of the analog ODE system approaches 0.8. These findings are consistent with the analysis presented in ***part 3*** concerning the impact of programming noise on model performance.

Experimentally, our analog memristor programming noise is below 2% through repeated programming cycles (see **Fig. S3** for a detailed programming scheme). Therefore, the influence of programming noise remains relatively minor.

REFERENCES AND NOTES

1. X. Lin, L. Kundu, C. Dick, E. Obiodu, T. Mostak, M. Flaxman, 6G digital twin networks: From theory to practice. *IEEE Commun. Mag.* **61**, 72–78 (2023).

2. X. Pan, N. Charron, Y. Yang, S. Peters, T. Whelan, C. Kong, O. Parkhi, R. Newcombe, Y. C. Ren, Aria digital twin: A new benchmark dataset for egocentric 3d machine perception, in *Proceedings of the IEEE/CVF International Conference on Computer Vision* (IEEE, 2023), pp. 20133–20143.

3. NVIDIA Omniverse, *Digital twins overview* (2023); https://docs.omniverse.nvidia.com/digital-twins/latest/index.html.

4. L. Chang, Z. Zhang, P. Li, S. Xi, W. Guo, Y. Shen, Z. Xiong, J. Kang, D. Niyato, X. Qiao, Y. Wu, 6G-enabled edge ai for metaverse: Challenges, methods, and future research directions. *J. Commun. Inf. Netw.* **7**, 107–121 (2022).

5. A. Tzachor, O. Hendel, C. E. Richards, Digital twins: A stepping stone to achieve ocean sustainability? *NPJ Ocean Sustain.* **2**, 16 (2023).

6. S. Kim, S. Heo, An agricultural digital twin for mandarins demonstrates the potential for individualized agriculture. *Nat. Commun.* **15**, 1561 (2024).

7. Z. Lei, H. Zhou, X. Dai, W. Hu, G.-P. Liu, Digital twin based monitoring and control for DC-DC converters. *Nat. Commun.* **14**, 5604 (2023).

8. X. Li, M. Feng, Y. Ran, Y. Su, F. Liu, C. Huang, H. Shen, Q. Xiao, J. Su, S. Yuan, H. Guo, Big data in earth system science and progress towards a digital twin. *Nat. Rev. Earth Environ.* **4**, 319–332 (2023).

9. S. A. Niederer, M. S. Sacks, M. Girolami, K. Willcox, Scaling digital twins from the artisanal to the industrial. *Nat. Comput. Sci.* **1**, 313–320 (2021).

10. F. Tao, H. Zhang, A. Liu, A. Y. C. Nee, Digital twin in industry: State-of-the-art. *IEEE Trans. Industr. Inform.* **15**, 2405–2415 (2019).

11. R. Laubenbacher, A. Niarakis, T. Helikar, G. An, B. Shapiro, R. S. Malik-Sheriff, T. J. Sego, A. Knapp, P. Macklin, J. A. Glazier, Building digital twins of the human immune system: toward a roadmap. *NPJ Digit. Med.* **5**, 64 (2022).

12. Y. Hui, X. Ma, Z. Su, N. Cheng, Z. Yin, T. H. Luan, Y. Chen, Collaboration as a service: Digital-twin-enabled collaborative and distributed autonomous driving. *IEEE Internet Things J.* **9**, 18607–18619 (2022).

13. J. Argota Sánchez-Vaquerizo, Getting real: The challenge of building and validating a large-scale digital twin of barcelona's traffic with empirical data. *ISPRS Int. J. Geo Inf.* **11**, 24 (2022).

14. M. G. Kapteyn, J. V. R. Pretorius, K. E. Willcox, A probabilistic graphical model foundation for enabling predictive digital twins at scale. *Nat. Comput. Sci.* **1**, 337–347 (2021).

15. K. Prantikos, L. H. Tsoukalas, A. Heifetz, Physics-informed neural network solution of point kinetics equations for a nuclear reactor digital twin. *Energies* **15**, 7697 (2022).

16. G. Caldarelli, E. Arcaute, M. Barthelemy, M. Batty, C. Gershenson, D. Helbing, S. Mancuso, Y. Moreno, J. J. Ramasco, C. Rozenblat, A. Sánchez, J. L. Fernández-Villacañas, The role of complexity for digital twins of cities. *Nat. Comput. Sci.* **3**, 374–381 (2023).

17. R. Ganguli, S. Adhikari, The digital twin of discrete dynamic systems: Initial approaches and future challenges. *App. Math. Model.* **77**, 1110–1128 (2020).

18. K. Agalianos, S. T. Ponis, E. Aretoulaki, G. Plakas, O. Efthymiou, Discrete event simulation and digital twins: Review and challenges for logistics. *Procedia Manuf.* **51**, 1636–1641 (2020).

19. P. R. Vlachas, W. Byeon, Z. Y. Wan, T. P. Sapsis, P. Koumoutsakos, Data-driven forecasting of high-dimensional chaotic systems with long short-term memory networks. *Proc. R. Soc. A* **474**, 20170844 (2018).

20. P. Barbiero, R. Viñas Torné, P. Lió, Graph representation forecasting of patient's medical conditions: Toward a digital twin. *Front. Genet.* **12**, 652907 (2021).

21. S. Bouzid, P. Viarouge, J. Cros, Real-time digital twin of a wound rotor induction machine based on finite element method. *Energies* **13**, 5413 (2020).

22. X. Xie, A. K. Parlikad, R. S. Puri, A neural ordinary differential equations based approach for demand forecasting within power grid digital twins, in 2019 *IEEE International Conference on Communications, Control, and Computing Technologies for Smart Grids (SmartGridComm)* (IEEE, 2019), pp. 1–6.

23. M. A. Zidan, Y. Jeong, J. Lee, B. Chen, S. Huang, M. J. Kushner, W. D. Lu, A general memristor-based partial differential equation solver. *Nat. Electron.* **1**, 411–420 (2018).

24. E. Linn, R. Rosezin, S. Tappertzhofen, U. Böttger, R. Waser, Beyond von neumann–Logic operations in passive crossbar arrays alongside memory operations. *Nanotechnology* **23**, 305205 (2012).

25. A. Sebastian, M. Le Gallo, R. Khaddam-Aljameh, E. Eleftheriou, Memory devices and applications for in-memory computing. *Nat. Nanotechnol.* **15**, 529–544 (2020).

26. M. A. Zidan, J. P. Strachan, W. D. Lu, The future of electronics based on memristive systems. *Nat. Electron.* **1**, 22–29 (2018).

27. J. Shalf, The future of computing beyond Moore's Law. *Philos. Trans. R. Soc. A* **378**, 20190061 (2020).

28. R. K. Cavin, P. Lugli, V. V. Zhirnov, Science and engineering beyond Moore's Law. *Proc. IEEE* **100**, 1720–1749 (2012).

29. T. P. Lillicrap, J. J. Hunt, A. Pritzel, N. Heess, T. Erez, Y. Tassa, D. Silver, D. Wierstra, Continuous control with deep reinforcement learning. arXiv:1509.02971 [cs.LG] (2015).

30. V. Iakovlev, M. Heinonen, H. Lähdesmäki, Learning continuous-time pdes from sparse data with graph neural networks. arXiv:2006.08956 [cs.LG] (2020).

31. R. Hasani, M. Lechner, A. Amini, D. Rus, R. Grosu, Liquid time-constant networks, in *Proceedings of the AAAI Conference on Artificial Intelligence* (Association for the Advancement of Artificial Intelligence, 2021), vol. 35, pp. 7657–7666.

32. S. Wang, S. Suo, W.-C. Ma, A. Pokrovsky, R. Urtasun, Deep parametric continuous convolutional neural networks, in *Proceedings of the IEEE Conference on Computer Vision and Pattern Recognition* (IEEE, 2018), vol. 2589–2597.

33. J. Ho, A. Jain, P. Abbeel, Denoising diffusion probabilistic models. *Adv. Neural Inf. Process. Syst.* **33**, 6840–6851 (2020).

34. R. T. Q. Chen, Y. Rubanova, J. Bettencourt, D. K. Duvenaud, Neural ordinary differential equations. *Adv. Neural Inf. Process. Syst.* **31**, 6571–6583 (2018).

35. R. Hasani, M. Lechner, A. Amini, L. Liebenwein, A. Ray, M. Tschaikowski, G. Teschl, D. Rus, Closed-form continuous-time neural networks. *Nat. Mach. Intell.* **4**, 992–1003 (2022).

36. A. F. Queiruga, N. B. Erichson, D. Taylor, M. W. Mahoney, Continuous-in-depth neural networks. arXiv:2008.02389 [cs.LG] (2020).

37. S. Bai, J. Z. Kolter, V. Koltun, Deep equilibrium models. *Adv. Neural Inf. Process. Syst.* **32**, 690–701 (2019).

38. N. Guo, Y. Huang, T. Mai, S. Patil, C. Cao, M. Seok, S. Sethumadhavan, Y. Tsividis, Energy-efficient hybrid analog/digital approximate computation in continuous time. *IEEE J. Solid-State Circuits* **51**, 1514–1524 (2016).

39. Z. Wang, H. Wu, G. W. Burr, C. S. Hwang, K. L. Wang, Q. Xia, J. J. Yang, Resistive switching materials for information processing. *Nat. Rev. Mater.* **5**, 173–195 (2020).

40. M. Rao, H. Tang, J. Wu, W. Song, M. Zhang, W. Yin, Y. Zhuo, F. Kiani, B. Chen, X. Jiang, H. Liu, H.-Y. Chen, R. Midya, F. Ye, H. Jiang, Z. Wang, M. Wu, M. Hu, H. Wang, Q. Xia, N. Ge, J. Li, J. J. Yang, Thousands of conductance levels in memristors integrated on CMOS. *Nature* **615**, 823–829 (2023).

41. M. Le Gallo, A. Sebastian, R. Mathis, M. Manica, H. Giefers, T. Tuma, C. Bekas, A. Curioni, E. Eleftheriou, Mixed-precision in-memory computing. *Nat. Electron.* **1**, 246–253 (2018).

42. C. Li, Y. L. Hu, H. Jiang, N. Ge, E. Montgomery, J. Zhang, W. Song, N. Dávila, C. E. Graves, Z. Li, J. P. Strachan, P. Lin, Z. Wang, M. Barnell, Q. Wu, R. S. Williams, J. J. Yang, Q. Xia, Analogue signal and image processing with large memristor crossbars. *Nat. Electron.* **1**, 52–59 (2018).

43. W. Wan, R. Kubendran, C. Schaefer, S. B. Eryilmaz, W. Zhang, D. Wu, S. Deiss, P. Raina, H. Qian, B. Gao, S. Joshi, H. Wu, H.-S. P. Wong, G. Cauwenberghs, A compute-in-memory chip based on resistive random-access memory. *Nature* **608**, 504–512 (2022).

44. F. Cai, S. Kumar, T. Van Vaerenbergh, X. Sheng, R. Liu, C. Li, Z. Liu, M. Foltin, S. Yu, Q. Xia, J. J. Yang, R. Beausoleil, W. D. Lu, J. P. Strachan, Power-efficient combinatorial optimization using intrinsic noise in memristor Hopfield neural networks. *Nat. Electron.* **3**, 409–418 (2020).

45. D. van Kekem, "Dynamics of the Lorenz-96 model: Bifurcations, symmetries and waves," thesis, University of Groningen (2018).

46. K. He, X. Zhang, S. Ren, J. Sun, Deep residual learning for image recognition, in *Proceedings of the IEEE conference on computer vision and pattern recognition* (IEEE, 2016), pp. 770–778.

47. W. Lee, K. Kim, W. Jeong, L. A. Zotti, F. Pauly, J. C. Cuevas, P. Reddy, Heat dissipation in atomic-scale junctions. *Nature* **498**, 209–212 (2013).

48. H. H. Radamson, H. Zhu, Z. Wu, X. He, H. Lin, J. Liu, J. Xiang, Z. Kong, W. Xiong, J. Li, H. Cui, J. Gao, H. Yang, Y. Du, B. Xu, B. Li, X. Zhao, J. Yu, Y. Dong, G. Wang, State of the art and future perspectives in advanced CMOS technology. *Nanomaterials* **10**, 1555 (2020).

49. T. N. Theis, H.-S. P. Wong, The end of Moore's Law: A new beginning for information technology. *Comput. Sci. Eng.* **19**, 41–50 (2017).

50. R. R. Schaller, Moore's Law: Past, present and future. *IEEE Spectrum* **34**, 52–59 (1997).

51. J. Fan, F. Han, H. Liu, Challenges of big data analysis. *Natl. Sci. Rev.* **1**, 293–314 (2014).

52. G. Karunaratne, M. Le Gallo, G. Cherubini, L. Benini, A. Rahimi, A. Sebastian, In-memory hyperdimensional computing. *Nat. Electron.* **3**, 327–337 (2020).

53. S. Woźniak, A. Pantazi, T. Bohnstingl, E. Eleftheriou, Deep learning incorporating biologically inspired neural dynamics and in-memory computing. *Nat. Mach. Intell.* **2**, 325–336 (2020).

54. F. Kiani, J. Yin, Z. Wang, J. J. Yang, Q. Xia, A fully hardware-based memristive multilayer neural network. *Sci. Adv.* **7**, eabj4801 (2021).

55. D. B. Strukov, G. S. Snider, D. R. Stewart, R. S. Williams, The missing memristor found. *Nature* **453**, 80–83 (2008).

56. A. G. Radwan, M. A. Zidan, K. Salama, HP memristor mathematical model for periodic signals and DC, in *2010 53rd IEEE International Midwest Symposium on Circuits and Systems* (IEEE, 2010), pp. 861–864.

57. Y. Ham, J. Kim, Participatory sensing and digital twin city: Updating virtual city models for enhanced risk-informed decision-making. *J. Manage. Eng.* **36**, 04020005 (2020).

58. T. Goodwin, J. Xu, N. Celik, C.-H. Chen, Real-time digital twin-based optimization with predictive simulation learning. *J. Simul.* **18**, 47–64 (2024).

59. P. Jiang, C. Chen, X. Liu, Time series prediction for evolutions of complex systems: A deep learning approach, in *2016 IEEE International Conference on Control and Robotics Engineering (ICCRE)* (IEEE, 2016), pp. 1–6.

60. M. Cuturi, M. Blondel, Soft-dtw: A differentiable loss function for time-series. In *International Conference on Machine Learning* (PMLR, 2017), pp. 894–903.

61. X. Liu, T. Xiao, S. Si, Q. Cao, S. Kumar, C.-J. Hsieh, How does noise help robustness? explanation and exploration under the neural sde framework, in *Proceedings of the IEEE/CVF Conference on Computer Vision and Pattern Recognition* (IEEE, 2020), pp. 282–290.

62. S. Yin, Y. Kim, X. Han, H. Barnaby, S. Yu, Y. Luo, W. He, X. Sun, J.-J. Kim, J.-s. Seo, Monolithically integrated RRAM-and CMOS-based in-memory computing optimizations for efficient deep learning. *IEEE Micro* **39**, 54–63 (2019).

63. A. Paszke, S. Gross, F. Massa, A. Lerer, J. Bradbury, G. Chanan, T. Killeen, Z. Lin, N. Gimelshein, L. Antiga, A. Desmaison, A. Köpf, E. Yang, Z. De Vito, M. Raison, A. Tejani, S. Chilamkurthy, B. Steiner, L. Fang, J. Bai, S. Chintala, Pytorch: An imperative style, high-performance deep learning library. arXiv:1912.01703 [cs.LG] (2019).

64. P. Contributors, *Pytorch profiler–pytorch tutorials 2.6.0+cu124 documentation* (2024). Version 2.6.0+cu124. https://pytorch.org/tutorials/recipes/recipes/profiler_recipe.html.

65. S. Wang, Y. Li, D. Wang, W. Zhang, X. Chen, D. Dong, S. Wang, X. Zhang, P. Lin, C. Gallicchio, X. Xu, Q. Liu, K.-T. Cheng, Z. Wang, D. Shang, M. Liu, Echo state graph neural networks with analogue random resistive memory arrays. *Nat. Mach. Intell.* **5**, 104–113 (2023).

66. S. Ambrogio, P. Narayanan, H. Tsai, R. M. Shelby, I. Boybat, C. di Nolfo, S. Sidler, M. Giordano, M. Bodini, N. C. P. Farinha, B. Killeen, C. Cheng, Y. Jaoudi, G. W. Burr, Equivalent-accuracy accelerated neural-network training using analogue memory. *Nature* **558**, 60–67 (2018).

67. P. Yao, H. Wu, B. Gao, J. Tang, Q. Zhang, W. Zhang, J. J. Yang, H. Qian, Fully hardware-implemented memristor convolutional neural network. *Nature* **577**, 641–646 (2020).

68. J. W. Chen, X. K. Sigalingging, J.-S. Leu, J.-I. Takada, Applying a hybrid sequential model to chinese sentence correction. *Symmetry* **12**, 1939 (2020).

69. G. Chillcce, O. el Moctar, Data-driven system identification of hydrodynamic maneuvering coefficients from free-running tests. *Phys. Fluids* **35**, 057122 (2023).

70. A. Peussa, E.-P. Damskägg, T. Sherson, S. I. Mimilakis, L. Juvela, A. Gotsopoulos, V. Välimäki, Exposure bias and state matching in recurrent neural network virtual analog

models, in *2021 24th International Conference on Digital Audio Effects (DAFx)* (IEEE, 2021), pp. 284–291.

71. H. Fan, Y. Yang, Pointrnn: Point recurrent neural network for moving point cloud processing. arXiv:1910.08287 [cs.LG] (2019).

72. G. Lai, W.-C. Chang, Y. Yang, H. Liu, Modeling long-and short-term temporal patterns with deep neural networks, in *The 41st International ACM SIGIR Conference on Research & Development in Information Retrieval* (2018), pp. 95–104.

73. D. Cao, Y. Wang, J. Duan, C. Zhang, X. Zhu, C. Huang, Y. Tong, B. Xu, J. Bai, J. Tong, Q. Zhang, Spectral temporal graph neural network for multivariate time-series forecasting. *Adv. Neural Inf Process. Syst.* **33**, 17766–17778 (2020).

74. X. Chen, F. A. Araujo, M. Riou, J. Torrejon, D. Ravelosona, W. Kang, W. Zhao, J. Grollier, D. Querlioz, Forecasting the outcome of spintronic experiments with neural ordinary differential equations. *Nat. Commun.* **13**, 1016 (2022).

75. C. H. Skokos, G. A. Gottwald, J. Laskar, *Chaos detection and predictability* (Springer, 2016), vol. 1.

76. J. Pathak, Z. Lu, B. R. Hunt, M. Girvan, E. Ott, Using machine learning to replicate chaotic attractors and calculate Lyapunov exponents from data. *Chaos* **27**, 121102 (2017).

77. A. Wolf, J. B. Swift, H. L. Swinney, J. A. Vastano, Determining Lyapunov exponents from a time series. *Physica D* **16**, 285–317 (1985).

78. U. Parlitz, Estimating lyapunov exponents from time series. *Chaos detection and predictability* (2016), pp. 1–34.

79. M. T. Rosenstein, J. J. Collins, C. J. De Luca, A practical method for calculating largest lyapunov exponents from small data sets. *Physica D* **65**, 117–134 (1993).

80. R. J. Hyndman, G. Athanasopoulos, *Forecasting: principles and practice* (OTexts, 2018).

81. J. Korpela, K. Puolamäki, A. Gionis, Confidence bands for time series data. *Data Min and Knowl. Disc.* **28**, 1530–1553 (2014).

82. G. Wang, The 95% confidence interval for GNSS-derived site velocities. *J. Surv. Eng.* **148**, 04021030 (2022).

83. W. Song, M. Rao, Y. Li, C. Li, Y. Zhuo, F. Cai, M. Wu, W. Yin, Z. Li, Q. Wei, S. Lee, H. Zhu, L. Gong, M. Barnell, Q. Wu, P. A. Beerel, M. S.-W. Chen, N. Ge, M. Hu, Q. Xia, J. J. Yang, Programming memristor arrays with arbitrarily high precision for analog computing. *Science* **383**, 903–910 (2024).

84. S.-S. Sheu, M.-F. Chang, K.-F. Lin, C.-W. Wu, Y.-S. Chen, P.-F. Chiu, C.-C. Kuo, Y.-S. Yang, P.-C. Chiang, W.-P. Lin, C.-H. Lin, H.-Y. Lee, P.-Y. Gu, S.-M. Wang, F. T. Chen, K.-L. Su, C.-H. Lien, K.-H. Cheng, H.-T. Wu, T.-K. Ku, M.-J. Kao, M.-J. Tsai, A 4Mb embedded SLC resistive-RAM macro with 7.2 ns read-write random-access time and 160ns MLC-access capability, in 2011 IEEE International Solid-State Circuits Conference (IEEE, 2011), pp. 200–202.

85. W.-S. Khwa, Y.-C. Chiu, C.-J. Jhang, S.-P. Huang, C.-Y. Lee, T.-H. Wen, F.-C. Chang, S.-M. Yu, T.-Y. Lee, M.-F. Chang, A 40-nm, 2M-cell, 8b-precision, hybrid SLC-MLC PCM computing-in-memory macro with 20.5-65.0 TOPS/W for tiny-Al edge devices, in *2022 IEEE International Solid-State Circuits Conference (ISSCC)* (IEEE, 2022), vol. 65, 1–3.

86. W.-H. Huang, T.-H. Wen, J.-M. Hung, W.-S. Khwa, Y.-C. Lo, C.-J. Jhang, H.-H. Hsu, Y.-H. Chin, Y.-C. Chen, C.-C. Lo, R.-S. Liu, K.-T. Tang, C.-C. Hsieh, Y.-D. Chih, T.-Y. Chang, M.-F. Chang, A nonvolatile al-edge processor with 4mb slc-mlc hybrid-mode reram compute-in-memory macro and 51.4-251TOPS/W, in *2023 IEEE International Solid-State Circuits Conference (ISSCC)* (IEEE, 2023), pp. 15–17.

87. V. Aparin, L. E. Larson, Linearization of monolithic lnas using low-frequency low-impedance input termination, in *ESSCIRC 2004-29th European Solid-State Circuits Conference (IEEE Cat. No. 03EX705)* (IEEE, 2003), pp. 137–140.

88. H. Zhang, E. Sánchez-Sinencio, Linearization techniques for CMOS low noise amplifiers: A tutorial. *IEEE Trans. Circuits Syst.* **58**, 22–36 (2010).

89. J. Yang, X. Xue, X. Xu, Q. Wang, H. Jiang, J. Yu, D. Dong, F. Zhang, H. Lv, M. Liu, 24.2 A 14nm-FinFET 1Mb embedded 1T1R RRAM with a 0.022 μm^2 cell size using self-adaptive delayed termination and multi-cell reference, in *2021 IEEE International Solid-State Circuits Conference (ISSCC)* (IEEE, 2021), vol. 64, pp. 336–338.

90. C.-C. Chou, Z.-J. Lin, P.-L. Tseng, C.-F. Li, C.-Y. Chang, W.-C. Chen, Y.-D. Chih, T.-Y. J. Chang, An N40 256K×44 embedded RRAM macro with SL-precharge SA and low-voltage current limiter to improve read and write performance, in *2018 IEEE International Solid-State Circuits Conference-(ISSCC)* (IEEE, 2018), pp. 478–480.

91. Q. Liu, B. Gao, P. Yao, D. Wu, J. Chen, Y. Pang, W. Zhang, Y. Liao, C.-X. Xue, W.-H. Chen, J. Tang, Y. Wang, M.-F. Chang, H. Qian, H. Wu, 33.2 a fully integrated analog ReRAM based 78.4 TOPS/W compute-in-memory chip with fully parallel MAC computing, in *2020 IEEE International Solid-State Circuits Conference-(ISSCC)* (IEEE, 2020), pp. 500–502.

92. B. Crafton, C. Talley, S. Spetalnick, J.-H. Yoon, A. Raychowdhury, Characterization and mitigation of IR-drop in RRAM-based compute in-memory, in *2022 IEEE International Symposium on Circuits and Systems (ISCAS)* (IEEE, 2022), pp. 70–74.

93. N. Lepri, A. Glukhov, D. Ielmini, Mitigating read-program variation and IR drop by circuit architecture in RRAM-based neural network accelerators, in *2022 IEEE International Reliability Physics Symposium (IRPS)* (IEEE, 2022), pp. 3C–2.

94. D. Ielmini, F. Nardi, C. Cagli, Resistance-dependent amplitude of random telegraph-signal noise in resistive switching memories. *Appl. Phys. Lett.* **96**, 053503 (2010).

95. Y. Li, Z. Wang, R. Midya, Q. Xia, J. J. Yang, Review of memristor devices in neuromorphic computing: Materials sciences and device challenges. *J. Phys. D Appl. Phys.* **51**, 503002 (2018).

96. N. Carlini, D. Wagner, Towards evaluating the robustness of neural networks, in *2017 IEEE Symposium On Security And Privacy (sp)* (IEEE, 2017), pp. 39–57.

97. D. Hendrycks, T. Dietterich, Benchmarking neural network robustness to common corruptions and perturbations. arXiv:1903.12261 [cs.LG] (2019).

98. Z. You, J. Ye, K. Li, Z. Xu, P. Wang, Adversarial noise layer: Regularize neural network by adding noise, in *2019 IEEE International Conference on Image Processing (ICIP)* (IEEE, 2019), pp. 909–913.

99. A. Liu, X. Liu, H. Yu, C. Zhang, Q. Liu, D. Tao, Training robust deep neural networks via adversarial noise propagation. *IEEE Trans. Image Process.* **30**, 5769–5781 (2021).
